# Stepping toward implementation using co-design: development of hospital protocols and resources for using wearable activity trackers in a hospital service

**DOI:** 10.3389/fdgth.2025.1520991

**Published:** 2025-03-18

**Authors:** Kimberley Szeto, Aaron Davis, John Arnold, Ian Gwilt, Aislin Forrest, Isaac Heyne, Anthony Hewitt, Peter Diestel-Feddersen, Dominique Edwards, Ben Singh, Carol Maher

**Affiliations:** ^1^ Alliance for Research in Exercise, Nutrition and Activity, University of South Australia, Adelaide, SA, Australia; ^2^ Australian Research Centre for Interactive and Virtual Environments (IVE), University of South Australia, Adelaide, SA, Australia; ^3^ Division of Rehabilitation, Aged and Palliative Care, Southern Adelaide Local Health Network, Adelaide, SA, Australia

**Keywords:** co-design, wearable activity tracker, hospital, healthcare, rehabilitation, physical activity, digital health

## Abstract

**Introduction:**

Low levels of patient physical activity during a hospital stay are linked to a variety of poor outcomes. Wearable activity trackers can help to boost patient activity and improve other outcomes during a hospitalisation, but a range of implementation barriers exist. Co-design research methodologies provide opportunities to bridge evidence-practice gaps, such as the implementation of wearable activity trackers to promote patient activity, by developing solutions and strategies in collaboration with key stakeholders. This co-design study aimed to develop a protocol and resources to support the implementation of wearable activity trackers into a rehabilitation service at a South Australian hospital.

**Methods:**

Three co-design workshops that employed an involvement partnership with 26 rehabilitation clinicians were conducted. User journey storyboards, empathy maps, and world café activities were used to understand processes of using technology with patients in the hospital, identify protocol components for using WATs, and create resources to support its implementation.

**Results:**

Using a co-design approach, this study developed a protocol for using WATs in a hospital rehabilitation services, identified key themes underpinning its implementation, and created a set of resources to support its delivery.

**Discussion:**

This study identified key elements to support implementation of WATs in hospital rehabilitation, and expands the evidence base for using co-design approaches in health research, and may support WAT implementation in other settings.

## Introduction

Each year, there is an estimated $170 billion of research waste from projects that have limited relevance for target stakeholders or settings (1). One way that health researchers can address the needs of stakeholders and develop context-specific solutions is with co-design research. Co-design is a participatory approach that uses the knowledge and experience of stakeholders to develop solutions or strategies to address a problem. Co-design research methodologies differ from other participatory research methodologies (e.g., focus groups), in that they involve stakeholders as “co-researchers” or “co-designers” who work *with* researchers to develop solutions, rather than involving stakeholders as subjects that researchers perform research “on” or about (2). Partnering with stakeholders to develop solutions means that co-design research can help bridge evidence-practice gaps, leading to outcomes that are specific to the context of their application.

As co-design research can support the development of solutions for evidence-practice gaps in healthcare, applications of co-design in health research, and digital health research specifically, are growing (3, 4). However, the evidence base describing what co-design research entails in terms of methods and activities, and engagement strategies that lead to successful partnerships between researchers and stakeholders remains scant (3), with limited published examples of co-design research undertaken during study planning phases (4). One area where there is an evidence-practice gap that co-design can help address is in using wearable activity trackers (WATs) in hospital and healthcare settings for physical activity promotion.

Physical activity (PA) is critical for health (5), particularly for patients in hospital and healthcare settings (6). Yet, very low levels of PA are typical during a hospitalisation, with most patients being sedentary for 87%–100% of their day (7, 8). Excessive inactivity during a hospitalisation is linked to higher mortality rates, functional deterioration, increased frailty, and disability (9, 10). Conversely, patients who maintain a higher level of PA during a hospitalisation tend to have shorter stays and are less likely to be readmitted (11, 12). Even very small amounts of PA are linked to improved patient outcomes. Walking between just 250 and 500 steps per day is associated with a reduced risk for prolonged length of stay, 30 day readmission, and discharge to non-home locations (13), and 900 steps daily reduces loss of function during a hospital stay (14). This highlights a need to prioritise PA promotion during hospital admissions to mitigate these risks.

There are growing efforts to boost patient PA in hospitals, including the implementation of mobility-focused policies (15), the development of PA guidelines for hospitalised older adults (16), and various behavioural interventions that target patient PA (17). One approach to PA promotion that is being applied more frequently in hospitals is the use of WATs. Modern WATs use accelerometers to measure PA (often metrics such as step counts, or minutes of PA) (18). There is a large and growing market for consumer-oriented WATs (such as Fitbits or Apple watches) (19), which include additional sensors (e.g., heart rate) and allow users to track multiple health metrics via the device and associated smartphone applications. In healthcare, WATs can enable clinicians to monitor and encourage patient activity, and they can motivate patients to move more by employing behaviour change strategies like self-monitoring, goal setting, and feedback (20). Indeed, interventions that use WATs in hospital settings have demonstrated their effectiveness for increasing PA, reducing sedentary behaviours, and improving physical function in a variety of hospitalised populations (21).

While WATs show promise in improving PA and other patient outcomes, their integration into hospital and healthcare broadly is so far limited. Despite interest and small-scale efforts to use WATs in healthcare, various barriers impact their uptake and integration, including practical issues (e.g., charging, wearing), knowledge gaps amongst patients, skill gaps and competing demands of clinicians, unclear protocols, and poor co-ordination across teams (22–24). To move toward WAT integration in healthcare, addressing the needs of end-users and developing strategies that consider these barriers and the healthcare contexts that they exist within are necessary (25, 26).

Implementation science plays an important role in addressing barriers to using WATs in healthcare. It focusses on putting research into practice, and can provide an approach for introducing and sustaining new innovations in healthcare by accounting for the innovation itself, and individual, organizational, and system level factors that impact uptake and maintenance (25). By considering these elements, implementation science can be used to support WAT integration in healthcare by learning how to support users and how to fit them into existing workflows. While previous works have looked at factors relating to WATs themselves and broader healthcare system-level considerations (27, 28), understanding implementation factors and planning an appropriate approach to WAT use requires input from the target end-users. Implementation science principles can be used within a co-design methodology by partnering with end-users to gain an understanding of local healthcare settings to develop approaches to WAT implementation that meet the needs and characteristics of target settings. Therefore, the aim of this study was twofold, (1) to co-design an approach for using WATs in a hospital setting, and (2) report on the activities and outcomes of the co-design methodology to develop such an approach. Specifically, the study sought to answer the following questions:

(1) What needs to be included in a protocol for routine use of WATs in the target setting?
(2) Which WAT best suits the needs of the setting? (e.g., based on required metrics, features, software).
(3) Who will need training to implement and use WATs?
(4) What supporting resources and information will be needed? (e.g., patient instructions, hospital protocol for WAT use)

By addressing these aims, this study contributes both to the practical implementation of WATs in healthcare, and advances our understanding of co-design methodologies in health research, potentially improving future efforts to bridge the gap between health innovations and their successful integration into clinical practice.

## Methods

### Study design and research team

A qualitative research methodology using a co-design (participatory) framework was used to address the study objectives (29), which included a series of three iterative workshops. Findings of this study are reported according to the Consolidated Criteria for Reporting Qualitative Research (COREQ) (30). Ethical approval was provided by the University of South Australia's Human Research Ethics Committee and written informed consent was obtained from participants. The research team comprised a PhD candidate, two senior researchers in physical activity and digital health, two design researchers with extensive experience in co-design and health, and two undergraduate physiotherapy students.

### Setting

This co-design study was conducted in collaboration with a rehabilitation department in a South Australian hospital, with the intention to use developed resources in the virtual rehabilitation ward (VRW) within the department. The VRW is an inpatient rehabilitation service in which patients are admitted to the hospital for rehabilitation. However instead of staying in a ward, they receive inpatient rehabilitation care in their homes for the duration of their admission. Thus, hospital care is provided through a combination of home visits and via telehealth. The healthcare team includes medical specialists, nurses, and various allied health disciplines, and patients receive equipment loans for required technology (e.g., tablets) and rehabilitation equipment (e.g., exercise equipment, chairs). The VRW was chosen as a target service for this study due to recommendation from the department director at the time of planning, and its existing technology infrastructure making it a suitable first point.

### Co-design approach

The co-design approach focused on engaging clinicians from the hospital's rehabilitation department in a partnership with researchers. This means the research was conceived as being conducted “with” these clinicians rather than “on”, “for”, or “by” these clinicians. Critically, and in contrast to a focus-group based research approach, the partnership focused on enabling active contributions from both participants and the research team (31). The overarching structure of the workshop series was developed in line with the British Design Council's Double Diamond Design process (32), combining generative divergent activities with analytical convergent activities. This resulted in the three workshops being structured:

- Discover (workshop 1): Understanding the process of implementing technology and identifying and mapping “pain points”.
- Define (workshops 2 and 3): Exploring how WATs can be used in a meaningful way to enhance rehabilitation while considering processes and pain points identified in workshop 1.
- Develop (post- workshop 3, and feedback on resources): Creation of resources following workshops and obtaining feedback on prototypes.
- Deliver (future research): Pilot feasibility study that will evaluate resources developed.

The co-design process was conducted across two quadrants of Davis et al.'s (33) spatiotemporal framework for co-design: same-time, same-space; and different-time, different-space. Due to rostering constraints, the workshops with clinicians needed to be conducted within a 1-h timeframe. This constraint further emphasised the need for the development process to be undertaken as a partnership “with” clinicians, rather than as an enablement “of” clinicians, i.e., for the co-design to be a collaboration between researchers and clinicians, rather than facilitating clinicians to undertake a design process. Workshops were conducted face-to-face in the workplace during work hours. Because of the time constraints in these sessions, between each of the workshops, the research team undertook iterative exploratory development of prototypes and materials, with the workshops used primarily as the starting catalyst for these explorations, and to review, provide feedback, and suggest iterations to these prototypes.

Participation was designed so that the processes being undertaken represented a strong partnership and collaboration with clinicians, with the majority of workshop sessions focused on accessing their insights and ideas. However, for decision making during the process, the limited time available for engagement meant it was not possible, nor appropriate to imbue participants with decision making power (34). This means, if reporting the process on the International Association for Public Participation (IAP)'s Spectrum of Public Participation in research, it would be described as an *involvement* approach, where participant engagement was used to ensure that participants' experiences and concerns were understood and considered in the design process (35).

### Participants and recruitment

Participants were recruited via an email from the department director of physiotherapy and exercise physiology. Eligible participants were invited via email prior to each workshop (August 2022, September 2022, and May 2023). Participants were encouraged to join all workshops, and were able to join the study at any workshop regardless of attendance of earlier workshops. While standard engagement for co-design studies is 10–12 participants, we anticipated variation in the group across the study, so targeted 20 participants in total, and approximately 10–15 participants for each workshop. Informed consent was obtained from all participants prior to their engagement in workshops.

Participants were clinicians working in the rehabilitation department at Flinders Medical Centre. We invited and aimed to include clinicians of varied disciplines (e.g., physiotherapy, exercise physiology, medical and nursing, other allied health), and representing various services within the department (e.g., VRW, inpatient rehabilitation, home rehabilitation). We included clinicians from different services as the planned activities required sharing experiences and expertise from various wards and services. Many clinicians from the hospital frequently rotate between different services, and have experience providing care for the type of patients that would present to the VRW (the intended setting) as well as other services. Because WATs are a relatively new technology in healthcare settings, we did not exclude based on professional or clinical experience using WATs.

### Co-design workshops

**Figure 1:**
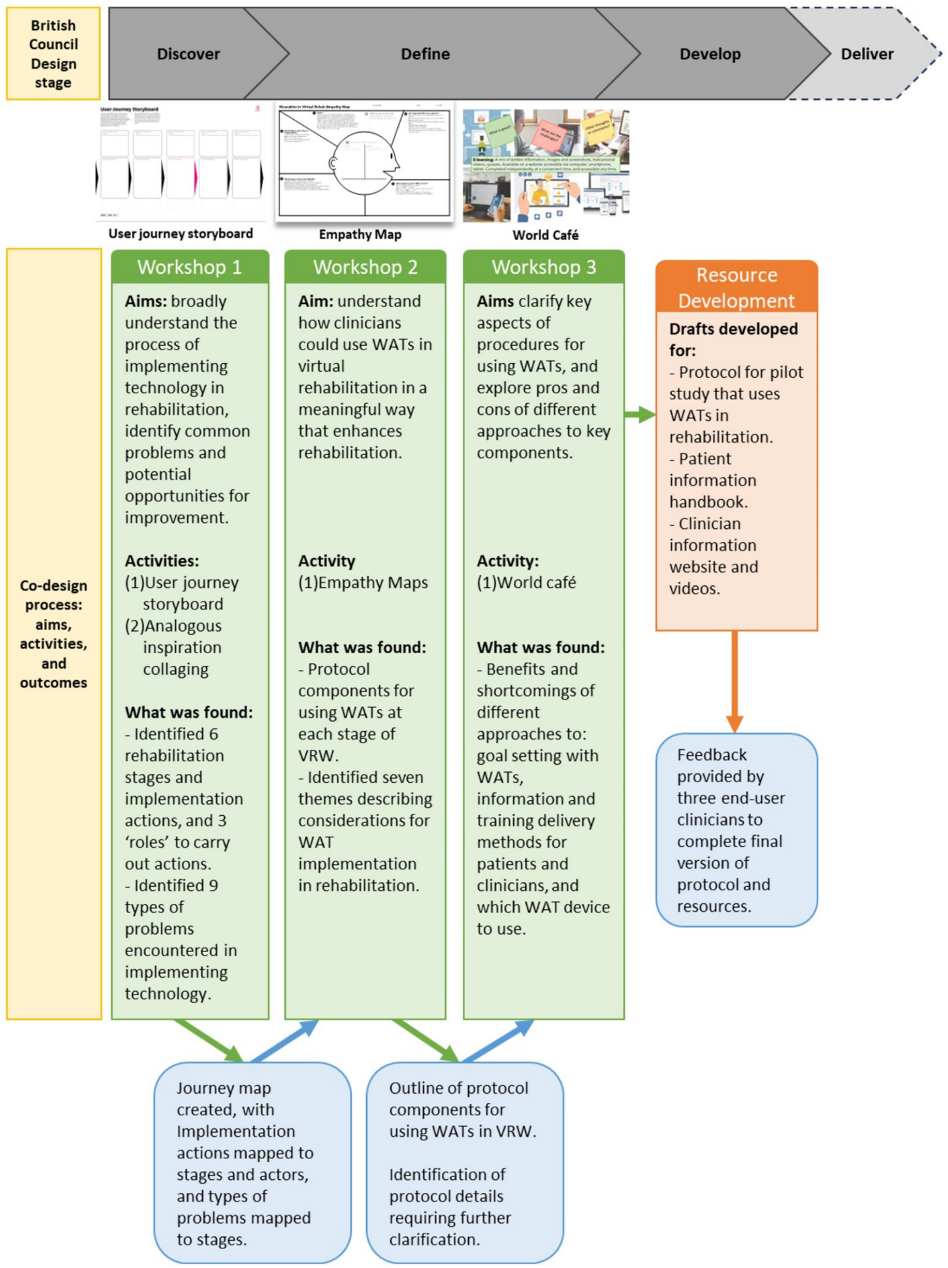
Co-design and workshops overview.

Co-design workshops were conducted in-person in a meeting room at the hospital where participants worked, and were led by AD and KS with other authors acting as facilitators to activities. Workshops were conducted during a scheduled staff meeting, and each ran for 1 h. While not ideal from a co-design perspective, the short duration was decided upon in partnership with participants, acknowledging that the participation would likely only be possible during times already designated for staff meetings. Snacks were provided at all workshops to encourage participation and to promote a friendly and collegial atmosphere. Researchers met with clinician partners from the hospital (authors AH, PDF, and DE) between each workshop. Clinician partners were included in meetings with the research team to review progress and suitability of the completed work and facilitate planning for subsequent sessions. While these meetings were not open to all participants, they played a key role in maintaining momentum and ensuring that clinician insights were incorporated throughout the process. An overview of each workshop and the iterative co-design process is provided in Figure 1, and all activity worksheets are provided in Supplementary Materials S1–S3.

Across all workshops, templates based on the principles of exploratory design games (36) and Liberating Structures (37) were used to increase engagement and maximise the amount of discussion that could take place within the 1-h timeframe. Design games use game-based elements such as chance, constraints, rules, negotiation, objectives, and perspectives to stimulate discussion and creative responses (36). These approaches allow participants to self-facilitate and to take ownership of the direction of their explorations, reducing the power imbalance between researchers and participants. Further, the use of templates enables a participant-led documentation process that allows participants to record their own interpretation of contributions, in addition to audio recorded verbal contributions. This approach further contributes to the empowerment of participants, placing responsibility for documentation in their hands rather than relying on a scribe and *post-hoc* thematic analysis as in a focus group.

Workshop one: The first workshop aimed to broadly understand the process of implementing technology in the department, and to identify common problems and potential opportunities for improvement. A user journey storyboard activity that outlined the process of using tablets (iPads) with patients was used for clinicians to draw on their experience implementing technology, as WATs had not been previously used with patients. Participants outlined the “journey” of implementing tablets into care by defining and identifying what happened at discrete stages. They could start with any action taken to implement tablet use (i.e., tablet given to patient) and worked forward and backward to identify steps that happened before and after. After the tablet “journey” had been outlined, participants identified and prioritised problems encountered at different stages. The second activity was a collaging analogous inspiration exercise (38) that explored the problems identified in the first activity as most important. This required participants to identify the different components of the problem (e.g., why it might have occurred, who is involved), to better understand why they occurred and how they could be addressed. All activities were completed in self-formed groups of 2–3 participants, and facilitated by researchers. Activity worksheets for workshop one are available in Supplementary Material S1.

Workshop two: The second workshop aimed to understand how clinicians could use WATs with patients in the VRW in a meaningful way that enhanced rehabilitation. At the start of workshop two, the results from workshop one were presented as a resulting storyboard to participants, with the opportunity to provide feedback. The main activity for workshop two was a 'story telling' activity using empathy maps (39), which presented each participant with one of six hypothetical patient scenarios. The scenarios included “pain points” identified from workshop one which may have made using WATs more challenging (e.g., patients having low familiarity with technology, or not engaged with therapy). Participants detailed what needed to happen at each stage of the rehabilitation journey for WATs to be used successfully with the hypothetical patient, and identified behaviour change techniques that could be applied to support patients using WATs and achieve therapy goals. This helped identify practical components of protocols for using WATs in rehabilitation. Participants were also encouraged to identify possible thoughts and feelings the patient may have while using WATs, and what challenges and successes they may experience. This helped to identify strategies that could be used to increase the likelihood of success when using WATs with patients. After completing empathy maps, participants were invited to verbally share with the group the three most important points from their patient scenario. Activity worksheets for workshop two are available in Supplementary Material S2.

Workshop three: The third workshop aimed to clarify key aspects of the procedures for using WATs in the VRW, by exploring the benefits and shortcomings of different approaches to: (1) goal setting, (2) providing training and information to clinicians, (3) providing information and instructions to patients, and (4) the device used. Because there had been a large break between workshops two and three, at the start of the workshop, a short summary of workshop two was provided, which presented themes and protocol components identified in previous workshops. To address the aims of workshop three, a World Café (40) activity was undertaken. Four different stations were set up that corresponded to each of the components of interest. An introduction to the activity was provided to the group and the topic for each station was outlined, which included background information on the topic and what we sought to understand about the topic for each station. Participants spent approximately 8 min at each station to discuss the different approaches presented with one another and researchers in groups of 3–5. Following the participant-led documentation process, each participant was invited to provide written responses for the presented approaches on each station, in addition to notes that were being made by the researcher(s). At each station, participants identified benefits, challenges, and opportunities in response to each component of interest that was presented. Activity worksheets for workshop three are available in Supplementary Material S3.

### Data analysis

Participant notes were collated and organised according to the aims for the workshop and activities. Three authors (KS, AF, and IH) summarised data for workshop one, while KS summarised workshops two and three. All activity sheets were scanned to retain digital copies, and handwritten notes were transcribed and organised by activity. Data from mapping activities in workshops one and two were grouped and organised in sequential order to outline the journey of using different technologies (tablets or WATs). Two thematic analysis approaches were used: inductive thematic analysis (41) was used to identify categories and themes from each workshop activity, organised by the activity's aim (i.e., pain points from workshop one, and empathy maps from workshop two); reflexive thematic analysis (42) was used to identify themes from the group share in workshop two, where participants acted as co-researchers, assigning themes to their completed empathy maps. Results from mapping exercises and themes were discussed and finalised in research team group meetings that took place between workshops. Data from the World Café activity in workshop three were summarised and guided the development of the resulting resources along with discussion amongst the research team and end-users.

### Resources development

Following workshop three, a protocol for a pilot study that would use WATs in the VRW and suite of supporting resources were developed. Three participants from the workshops, who would also be clinician end-users for the resources, were involved in this process, and reviewed the protocol and resources as they were completed.

## Results

### Participants

Twenty-six health care professionals participated in the three workshops, with *n* = 14, *n* = 12, and *n* = 10 healthcare professionals attending workshops one, two, and three, respectively. Two healthcare professionals attended all three workshops, five attended two workshops, and 19 attended one workshop. Participating healthcare professionals included physiotherapists (81%) and exercise physiologists (19%); no clinicians representing other disciples participated. Participants worked across a wide variety of services within the hospital, representing experience and expertise from 11 different services, with many (58%) working in multiple services. Participants' ages ranged from 23 to 60 years (median: 28 years). Clinical experience varied from <1 to 39 years, with 77% having less than 10 years of experience. Most participants had no experience using WATs for either clinical (81%) or personal (69%) use. Demographic characteristics of participants are outlined in Table 1.

**Table 1:** Participant characteristics.

Characteristic	*n* = (%)
Gender	
Female	15 (58%)
Male	11 (42%)
Age	
Median (range)	28 (23–60)
Clinical Discipline	
Physiotherapist	21 (81%)
Exercise Physiologist	5 (19%)
Years of clinical experience	
<1	6 (23%)
1–<5	5 (19%)
5–<10	9 (35%)
10–<15	2 (8%)
15–<20	0 (0%)
20–<25	1 (4%)
25+	3 (11%)
Service working in at time of participation*	
Inpatient Rehabilitation	9 (35%)
Home Rehabilitation	5 (19%)
Virtual Rehabilitation	4 (15%)
Outpatient/day Rehabilitation	5 (19%)
Geriatric Evaluation and Management	3 (11%)
Mental Health Unit	1 (4%)
Concussion Clinic	1 (4%)
Management	2 (8%)
Research	1 (4%)
Palliative care	2 (8%)
Pulmonary rehabilitation	3 (11%)
Number of services working in	
1	15 (58%)
>1	11 (42%)
Years of experience in current role	
<1	13 (50%)
1–5 years	8 (31%)
>5	4 (15%)
(No response)	1 (4%)
Experience using WATs	
Clinically (with patients)	5 (19%)
Personal/non-clinical	8 (31%)
Workshop attendance*	
Workshop one	14
Workshop two	12
Workshop three	10

* More than 1 response allowed.

### Workshop one

Six storyboards were generated by six groups. These were collated into a hybrid summary storyboard by the research team following the workshop. Participants identified specific actions taken when using tablets with patients and the corresponding rehabilitation time point. Six discrete stages in the rehabilitation journey where action was taken were identified: (1) pre-admission, (2) admission, (3) during rehabilitation, (4) pre-discharge, (5) discharge, and (6) post-discharge. Three key people to carry out actions were also identified as important: clinicians, patients, IT staff. The actions performed by each person at each stage were organised in a resulting storyboard to provide an overview of how tablets were implemented in rehabilitation (Supplementary Material S4). Among the insights generated through this activity were that tasks carried out by patients were supported closely by clinicians (i.e., clinicians provided tablets to patients and showed them how to use tablets during rehabilitation), and tasks carried out by IT staff were related to managing tablets and providing support (i.e., troubleshooting issues, and resetting after use).

Five Analogous Inspiration collages identifying different components of important pain points were completed by five groups. Subsequent pain points were identified and grouped from storyboards and Analogous Inspiration collages. Nine discrete problem types in 3 problem categories (technology, clinician, patient) were mapped to different stages of the rehabilitation journey. Problems occurred equally across categories (3/9 each), with the problem types documented most frequently being: connectivity issues, clinician inexperience, poor team co-ordination, low patient technology literacy, and unsuitability of patients for rehabilitation. All problem types occurred at the “admission” and “during rehabilitation” stages, and some problem types also occurred at the pre-admission and pre-discharge stage (Supplementary Material S4). Each problem type was used to postulate potential problems that may occur when using WATs, for which possible solutions were described. Education and provision of instructions was commonly listed as a solution, along with procedures placing minimal demands on patients, and selecting technology that is a good fit for the service. All solutions generated are available in Supplementary Material S4.

### Workshop two

Participants provided feedback on the resulting storyboard and pain points presented at the start of the workshop. The feedback included points that participants felt were missed in the first workshop, which mostly included further details on patient suitability (i.e., affected ability of patients to use technology when hearing or visually impaired, or where dexterity is impaired), or patient engagement (i.e., some patients refusing to use technology altogether, and involving family members/carers being an enabler to using technology where engagement is low). All feedback is available in Supplementary Material S4.

**Figure 2:**
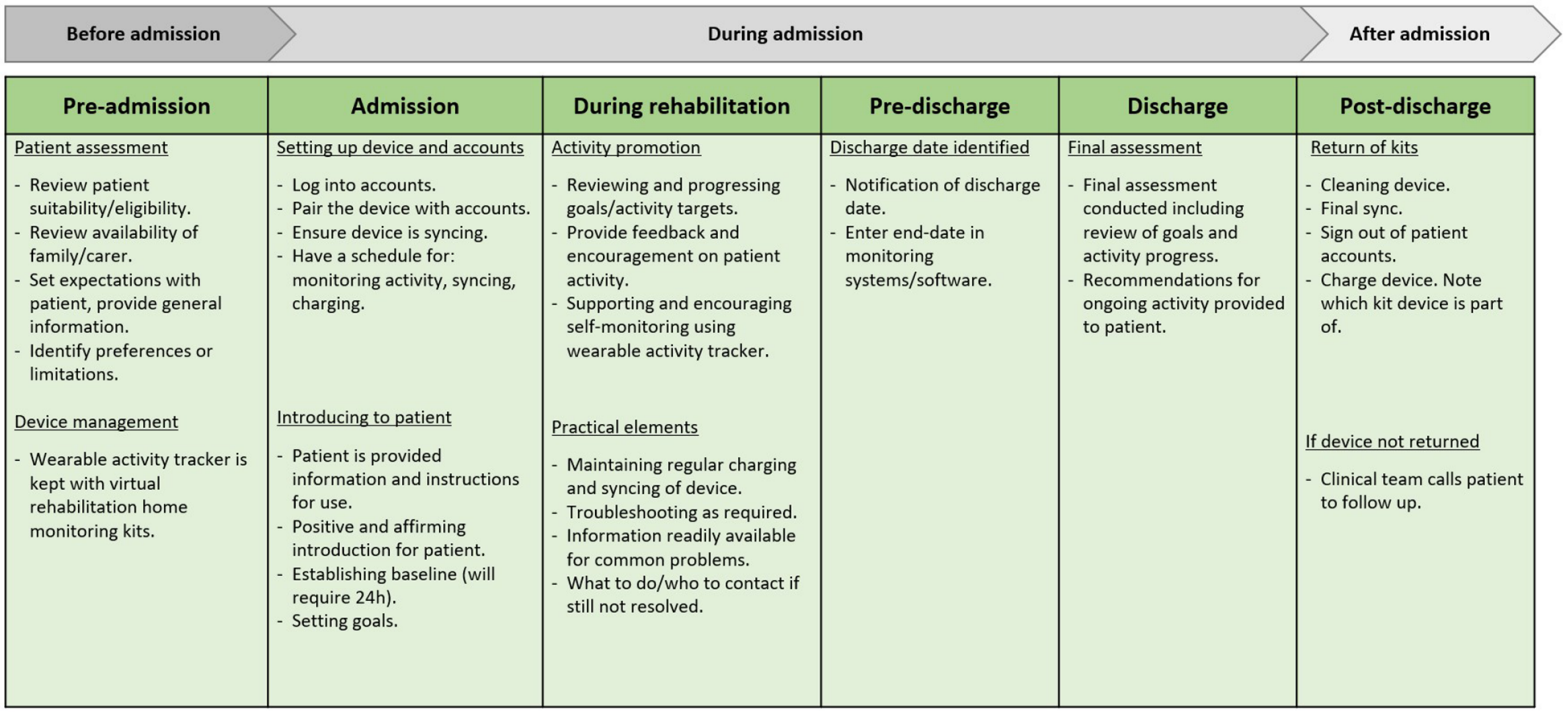
Protocol components.

Nine empathy maps were completed, and participants worked either independently or in groups of two. All participants and groups provided responses for sections on empathy maps that considered the practical elements of introducing WATs. Five of the ten groups also provided responses relating to the patients' thoughts and feelings about using WATs or other points to consider. The content of responses varied across groups, but there were commonalities and themes which aligned with stages of the rehabilitation journey. Responses were collated against each stage of the rehabilitation journey, to identify key actions and items to go into the protocol. Actions taken and protocol components occurring before the patient admission included device management and assessing suitability of new patients being admitted. Actions and components occurring during the admission period related to how WATs were introduced and used with the patient to promote activity, how devices were set up for new patients, and how practical elements of using WATs were handled. Protocol elements occurring following a patient's discharge related to the return of devices and how lost devices were managed. Figure 2 presents a summary of protocol components for using WATs at each stage of rehabilitation.

To prioritise what became a large and comprehensive exploration, participants each shared their three most important insights from completing the empathy maps. These participant-led themes were collated with themes identified from a deeper thematic analysis of the empathy mapping activity, resulting in seven overarching themes being identified. These themes describe key considerations for developing protocols and using WATs in rehabilitation, and were: (1) team and interdisciplinary co-ordination, (2) information and training provided to all involved with using WATs, (3) the WAT being a good fit for the service, (4) the WAT is used to support patients’ rehabilitation, (5) WATs are used in a manner that is positive and meaningful for patients, (6) there is a structured approach to providing WATs to patient's, and (7) there is a structured approach for managing and keeping track of WATs. Table 2 presents the overarching themes, along with their component subthemes and underpinning points from the group share and empathy map.

**Table 2:** Themes from workshop two.

Overarching theme	Themes from group share	Themes from empathy maps
Team and interdisciplinary co-ordination	• Involve family/carer/support • Entire clinical team knows how to use/troubleshoot (not just physio/EP) • Roles identified. Identify who helps with problem solving/trouble shooting • Regular clinician support available for patient	• Review availability of family/carer support • Certain clinicians may be more involved (PT/EP/AHAs/medical/nursing) • IT/admin manages loan devices • Follow-up call from clinical team if not returned • Clear, concise communication in various formats • Regular upskilling of staff
Information and training is required for those involved. This needs to include rationale and instructions for use, and may be provided in different formats	• Patient information to include: purpose (why WATs being used), instruction (how to use), and how to access help • Information provided in various formats (verbal, written handouts, images, practice) • Training for patient to include written info, and practical demonstration with opportunities to practice and succeed. • Visual information (i.e., screenshots) on printed info • Patient is able to handle most responsibilities independently, and understands what's involved • Patient information provided early	• Troubleshooting information provided to patient • Clinician information/training includes why and how • Family/carer information includes why and how • In person/practical training
The WAT needs to be a good fit for the service	• WAT use cohesive and “embedded” with current care • WAT links with other technology (e.g., tablets) • WAT data can be reviewed remotely by clinicians	• Data can be downloaded • Patient can trial/practise using device on ward to screen suitability
The WAT is used in various ways to support the patient rehab journey	• Patient able to review their progress/demonstration of improvements • WAT provides prompts and cues • WAT provides feedback on behaviour	• Establish baseline mobility/activity • Specific goals = better • Review of patient goals
The WATs are used in a way that is positive, and meaningful for patients	• WAT is introduced and explained in a positive and affirming manner. • WAT feedback/data is provided to patient in a meaningful way. • Meaningful, achievable, and progressive behavioural goals set. • Ongoing care/plan set for self-monitoring activity at discharge	• Making note of patient preferences or limitations • WAT use is patient-centred
There is a structured approach to providing the WAT to patients		• Review patient suitability/eligibility • Setting expectations for program • 0Check that WAT is working and charged • WAT provided at first contact • WAT provided before admission (i.e., on ward) • Provided by person who sets goals
There is a structured approach for managing and keeping track of devices		• WAT collected during discharge visit with other devices by last clinician visiting • Stored alongside other devices in kits • Clinician visiting area can collect if not returned • VRW team to store/manage • Patient delivers if not returned • Patient fined if not returned

The overarching themes (along with empathy maps responses) informed the development of the components of the protocol. In workshop three, reported below, further necessary details were identified that informed protocol development based on these overarching themes, including: information and training for those involved, the WAT being a good fit for the service, and the WAT being used to support rehabilitation and in a positive and meaningful way for the patient.

### Workshop three

Participants identified benefits and shortcomings of different approaches to each of the key aspects of WAT use presented in the World Café activity. A summary of how these were presented to participants is provided in Supplementary Material S3.

#### Station one: approaches to goal setting

Four different approaches to goal setting using WATs during rehabilitation were presented, ranging from highly standardised (i.e., standardised daily step goals the same for all patients), to highly individualised (i.e., individual step goal set for each patient). Individual participants had varied perspectives on the optimal approach, though common important principles for goal setting were identified: that goals should be set according to the patients' baseline presentation, clinical reasoning should underpin goals set, and that the same standardized step count targets would not be appropriate for all patients. There was a preference amongst participants for individualised approaches to goal setting.

#### Station two: approaches to clinician training

Four different methods of training and information delivery for clinicians using WATs with patients were presented, including: e-learning, face to face workshops, instruction manual, “go-to” person. Participants emphasised that having a “go-to” person was a useful strategy as it allowed for personal interactions and reassurance, and had previously supported the success of other projects. Many felt that online resources would be flexible and accessible for clinicians, though they preferred not to have quizzes assessing competency as a requirement. The inclusion of screenshots and videos was considered beneficial if the videos were short and didn't require audio to engage with. The interactive and hands-on opportunities of face-to-face training were viewed positively, but there were concerns about co-ordinating times with multiple people. Instruction manuals were considered boring and slow, and participants felt that it may not be that useful if using e-learnings. This confirmed that a combination of training methods, predominantly available online, would be most beneficial.

#### Station three: approaches to patient education

Five different formats and strategies for delivering instructions and information to patients using WATs were presented, including: online information, pamphlets, screenshots and images, practice with clinician, and involvement of family/carers. Physical pamphlets for patients were favoured over electronic information, with comments that the uptake would be better. Having practice with a clinician was noted as being important and relevant for patients with reduced cognition and capacity for following instruction, though the extra workload and resources required was noted. Involving a carer was identified as beneficial for patients with various impairments (e.g., cognition, visual, hearing), or where language was a barrier. A combination of approaches to patient education were identified as being appropriate, with providing a physical pamphlet and having a practice opportunity with a clinician being essential.

#### Station four: WAT device to use

Researchers conducted a desktop review of commercial and research-grade WATs, evaluating criteria for device characteristics, wear locations, accuracy, metrics, data management, and usability. Criteria were rated as green (good fit), yellow (unclear), or red (poor fit), based on earlier workshop results and related healthcare implementation studies (27, 28). The Fitbit Inspire 3 device emerged as most suitable, despite researchers identifying that wrist-worn devices may have reduced sensitivity measuring steps in some patients (i.e., slow walkers). Benefits and shortcomings of different body wear-location (wrist or ankle or both together) were explored by asking participants to identify which wear location they thought was most suitable. Wrist was identified as most suitable (*n* = 8/11). Few chose wrist and ankle together (*n* = 2/11) or ankle only (*n* = 1/11). Most clinicians firmly emphasized their preference for wrist-worn as most suitable, and felt that ankle worn would not be appropriate and sometimes unsafe for patients admitted for rehabilitation. Many benefits of wrist worn devices were noted, such as ease of engaging patients with behaviour change strategies (i.e., self-monitoring and feedback) and providing a preferable appearance. The only shortcoming reported for wrist worn devices was potentially getting in the way of some daily activities. The only benefit reported for ankle worn devices was that it was considered most accurate for use in a research trial. Numerous shortcomings of ankle worn devices were noted, and largely focussed on mobility restrictions and safety precautions of rehabilitation patients limiting self-monitoring activity using ankle-worn devices (i.e., falls risk or post-surgical contraindications related bending forward to reach ankle). The Fitbit Inspire 3 worn on the wrist was determined to be the most suitable WAT device for use in the VRW.

### Resources

The resources developed following the workshops included a patient instructional handbook, a clinician information and instructional website, and instructional videos (accessible on YouTube and via the website). End-user participants reviewed and provided feedback on the components and presentation of the resources before they were finalised.

**Figure 3:**
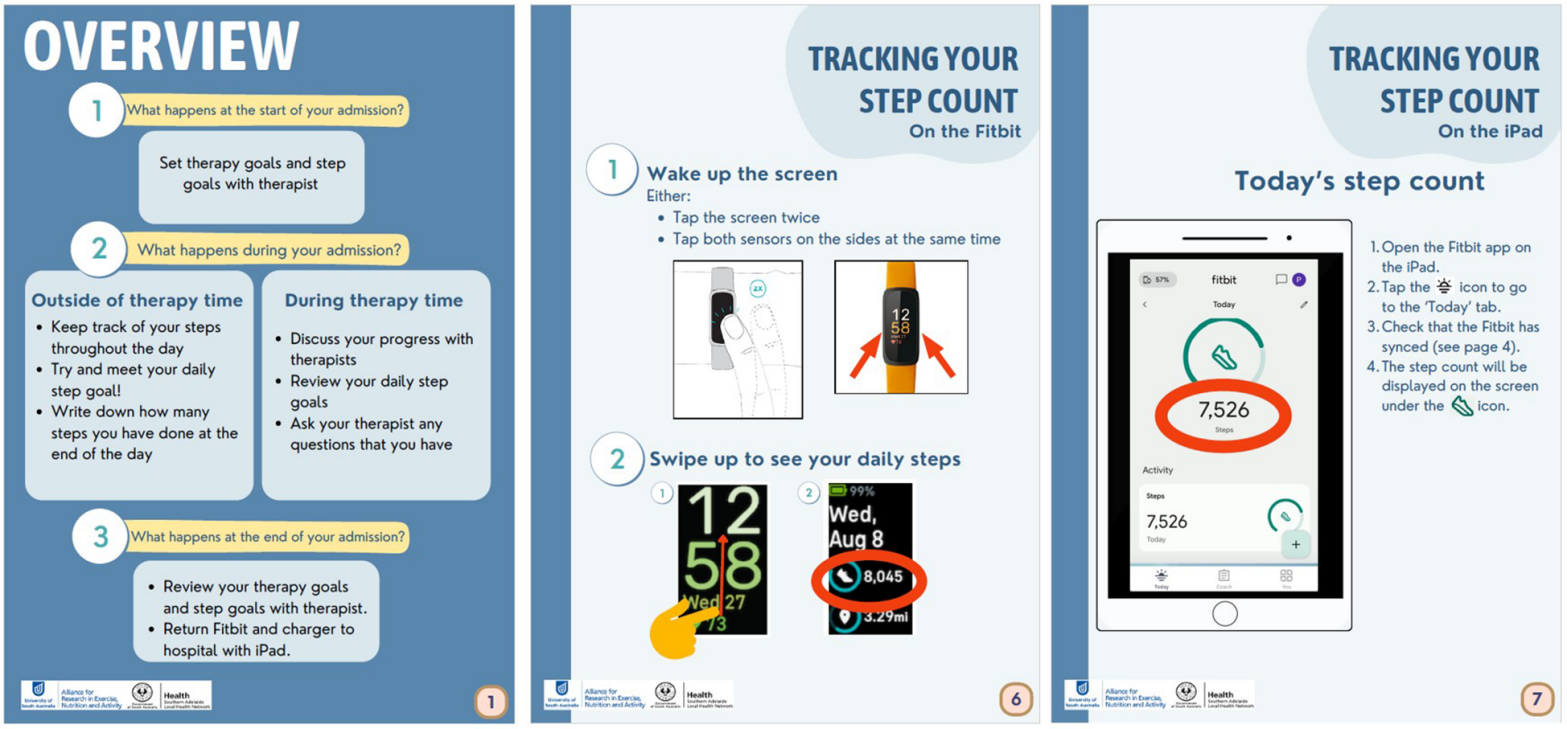
Example of patient materials.

**Figure 4:**
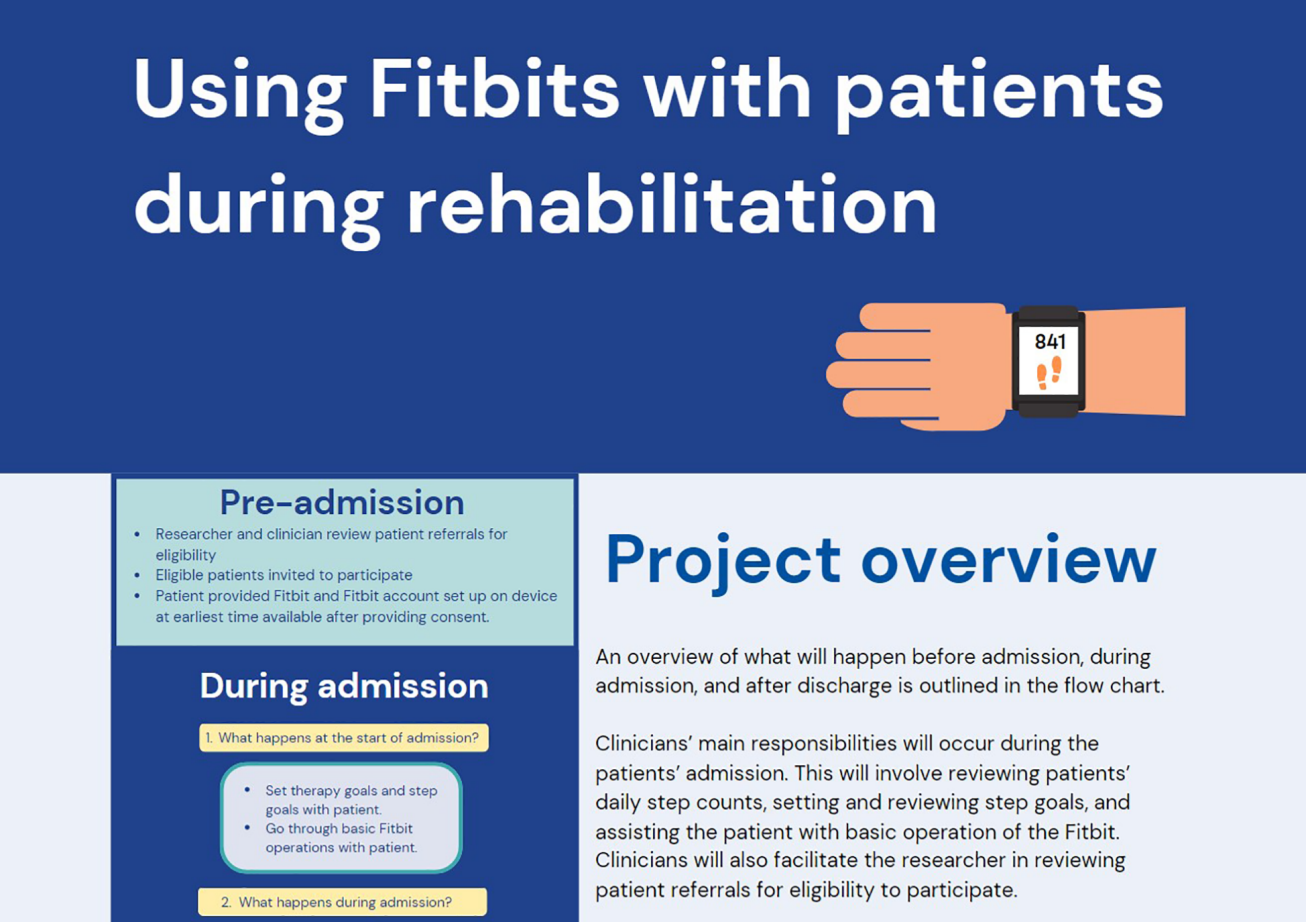
Example of clinician website.

The feedback provided by clinician end-users was that the resources were suitable, and minor suggestions were given for the presentation, which were addressed for the final version of resources. Suggestions included: making tracking of daily steps on a log sheet optional instead of required, simplifying the language in patient handbooks as much as possible, referring to the colours of icons used for the software and devices (i.e., “tap on the blue icon”), and embedding screenshots in a tablet image so it better reflected what patients would see on their own therapy tablet. Draft resources were revised based on this feedback, with examples of final versions presented in Figures 3, 4, and complete resources available in Supplementary Materials S5 and S6.

## Discussion

This co-design study employed a partnership between researchers and clinicians working in a hospital rehabilitation department to co-design a protocol and resources for using WATs in a virtual rehabilitation ward. Across a series of three workshops, we identified important protocol components across different stages of the rehabilitation journey for implementing WATs in the target rehabilitation service, and selected the Fitbit Inspire 3 as the most suitable WAT device for the setting. We also developed a set of resources to support protocol delivery, including a patient handbook and clinician website. Finally, this study also demonstrated the value of using co-design methodologies to address implementation challenges in healthcare settings.

### Using a co-design approach for healthcare applications

This co-design study is the first to our knowledge that develops a protocol and supporting resources for using WATs in a hospital setting. Other co-design studies have been conducted to develop approaches to monitoring and promoting patient PA with WATs and other technology, however these works have focussed heavily on creation of the technology itself, rather than how it will be deployed (43–45). While these works provide innovations in technological approaches to PA promotion in healthcare, they are limited in their broader scalability, as the technologies developed are not widely or commercially available beyond the projects described. Additionally, existing applications of co-design in this area place less emphasis on the implementation of such technologies and innovations in the healthcare settings they are intended for. Furthermore, detailed reporting on the activities undertaken and engagement approaches used in prior co-design studies is limited in this area, as well as in health research broadly (3, 4). Thus, this co-design study expands the evidence base for co-design in health by developing an approach focussed on the *implementation* of an innovation (rather than the innovation itself), and by providing a detailed description of activities undertaken to develop such an approach.

### Resources for WAT implementation developed in this co-design study

The co-design process resulted in the development of a trial protocol and resources to support the implementation of WATs in the VRW. The protocol's components were informed by the identification of discrete stages in the rehabilitation journey and the key stakeholders involved, with clinicians and researchers collaboratively determining the actions required for each stage to establish the protocol components and structure. The seven overarching themes identified during the co-design process were instrumental in informing specific details of the protocol and were determined by both researchers and clinicians. This approach ensures that the protocol is grounded in the reality of clinical workflow and addresses the practical challenges of WAT implementation.

Two key resources were developed including a patient handbook and a clinician website. The patient handbook, designed as a printed physical document, includes simple instructions for WAT use, step count monitoring, and wearing and charging the device, along with an optional log sheet for tracking daily steps. The use of images and straightforward language in the handbook addresses the need for clear, simple, and accessible patient information, which was identified as a key consideration in the co-design workshops. The clinician website contains information on WAT use, patient support, the associated software (to review patient activity data remotely), and guidance for goal setting using the WAT. By including links to patient instructions and demonstrative videos, the website facilitates consistent and informed use of WATs across the clinical team. The format and content of these resources directly reflects the priorities identified by participants in the co-design workshops, underscoring the value of stakeholder input in developing practical, user-centred tools for digital health implementation.

### Strengths and limitations

There are various strengths of this co-design study. First, its collaborative nature ensured that clinicians were engaged as partners throughout the development of procedures and resources. Researchers were guided by clinicians' input through each stage of the design process and shared each iteration of work produced with clinicians for feedback, which resulted in outcomes and products highly relevant to end-users. Second, the study employed innovative methodological approaches, including participant self-documentation (as responses on activities) and self-determination of themes (as shared to group). This allowed participants to provide their own interpretations of the content and ideas covered in workshops, enhancing the authenticity of the findings. A key strength lies in the detailed reporting of the co-design activities in detail, and the inclusion of co-design resources as Supplementary Materials. This comprehensive documentation serves two important purposes—other researchers planning to implement healthcare innovations may use the co-design materials to plan similar activities relevant to their particular innovation, while clinical users wanting to embed WATs into their healthcare setting may use and adapt the patient and clinician resources from this project. By offering this level of detail, our study contributes not only to the specific field of WAT implementation but also to the broader methodology of co-design in healthcare research.

There are also some limitations of this co-design study. The workshops ran for only one hour and most participants only attended one of the three workshops, limiting the amount and detail of clinician input provided. Conducting the workshops with short amounts of time may have limited the depth of detail provided by participants during workshops. There was a lack of diversity in the type of participants. Patients were not included as participants, and data collected about patient considerations when using WATs was posited by clinicians based on hypothetical patients, which may not accurately reflect the thoughts and feelings of actual patients who have completed rehabilitation. Additionally, only physiotherapists and exercise physiologists participated. While we attempted to invite other healthcare professions such as medical, nursing, other allied health, and management, this was unsuccessful and the resulting resources of this study does not include inputs from these stakeholders. There are also limitations for the generalisability of the resources developed. By design, the resulting protocol framework and resources were designed for the VRW in the hospital, and considered the unique contextual factors of this setting (such as admission and discharge pathways, and how other technology is used). To use the resources from this co-design study in different healthcare settings, adaptations that consider the context of the setting may be required (25).

### Implications for research

This article contributes to the evidence base for how a co-design methodology can be used in health research where implementation of innovations in healthcare is the end goal. It provides details and descriptions of how this approach can be applied to address implementation objectives, and can therefore support and guide other researchers who seek to use a co-design approach for protocol and resource development. Subsequent publication of similar co-design projects that provide details on activities involved will contribute to a noteworthy evidence gap broadly (4). Where feasible, other co-design work will likely benefit from researcher-stakeholder interactions of longer durations to allow for more in-depth exploration of topics, and to enable engagement approaches that balance decision-making more equally between researchers and stakeholders.

Future research that pursues WAT implementation will benefit by seeking input from other types of stakeholders who can provide contextual information on real-world WAT use. Specifically, we recognize that clinicians' insights were used to infer patient perspectives, which may not fully reflect patients' lived experiences. To address this in future work, we suggest engaging patients who have used WATs during rehabilitation and using methods that accommodate their needs, such as one-on-one interviews or small group sessions instead of traditional workshops. For nurses, we note that time constraints may have limited participation, and future studies could explore integrating co-design discussions into routine team meetings or conducting shorter, more targeted sessions focused on implementation logistics. Other future research directions are in the evaluation of the protocol and utility of resources developed from this co-design study.

### Implications for clinical practice

The protocol, resources, and overarching themes identified in this study can also inform and guide a range of clinical users and real-world applications of WATs in hospital or other healthcare settings. Specific details may be adapted to suit the application, but the results of this study provide a framework, principles to consider, and examples of protocol components and resources that can be used in a real-world health care setting.

## Conclusions

This co-design study successfully developed a protocol and suite of supporting resources for using WATs in a VRW. Through an involvement partnership between researchers and clinicians, we employed a series of activities to comprehensively understand the rehabilitation journey and the process of integrating technology into patient care. This enabled us to develop a protocol, grounded in clinical realities, identify key themes underpinning its implementation, and create relevant supporting resources. The partnership with clinicians ensured the resulting products were informed by the experiences and expertise of clinicians, making them highly relevant for the target setting. Future work will evaluate the feasibility and utility of the resources in practice. Importantly, the resources may also support clinical users intending to implement WATs in various healthcare settings. Moreover, the study provides a detailed example of how co-design methodologies can be applied in the planning and implementation of innovations in healthcare.

## Data Availability

The raw data supporting the conclusions of this article will be made available by the corresponding author upon reasonable request.
