# Supplementary material for: Stepping toward implementation using co-design: development of hospital protocols and resources for using wearable activity trackers in a hospital service
Source: Front Digit Health. 2025 Mar 18;7:1520991. doi: 10.3389/fdgth.2025.1520991 (PMC11959083; doi:10.3389/fdgth.2025.1520991)
Supplement: Supplementary file 1 [file Datasheet1.pdf]

Analogous Inspiration

the design clinic

We can often look to other sectors, other contexts, or other parts of life for inspiration for how to address our challenges. By interrogating what is at the core of the challenge or 'pain point' that has been identified, we can look for and learn from how these problems have been solved before.

1. Add your identified challenge or 'pain point' to the centre of this sheet
2. Identify key parts of this challenge in the black boxes
3. Add instances where each element has been solved in other industries elsewhere in the blank space.

Part of this challenge is:

Part of this challenge is:

This really needs to be improved:

Part of this challenge is:

Part of this challenge is:

This needs to be improved:

This is ok for now, but could be an opportunity for innovation:

This is ok for now, but could be an opportunity for innovation:

This really needs to be improved:

This is ok for now, but could be an opportunity for innovation:

Example: If the issue identified is that someone gets angry because a person who came into the ED after them is seen before them, the 'parts' or components of this challenge that could be identified might include the opacity of the complex system / process they are a part of, the stress of not knowing how long it will be until they are goign to be seen, or even something as specific as their mobile phone going flat and losing their source of entertainment.

Your analogous inspirations could then look to examples where these same 'parts' or components are present, from public transport systems, to restaurants/coffee shops, or even disneyland.

User-Journey Storyboard

A user-journey storyboard can be a shortcut to understanding the many different ways different people experience a situation or challenge. A storyboard can quickly capture details that would normally only be surfaced by intense and prolonged shadowing processes. There is no 'right' answer to a storyboard, and by completing multiple user-journeys, we can identify where there are differences, as well as similarities.

1. Start with the 'main event' in the middle box, add detail about what is happening, when it is happening, where it is happening, and who (or what) is present at each stage of the journey.
2. Work both backwards and forwards and add extra sheets to each end if you need more frames.

When is it happening?<br>Where?<br>Who or what technology is present?	When is it happening?<br>Where?<br>Who or what technology is present?	When is it happening?<br>Where?<br>Who or what technology is present?	When is it happening?<br>Where?<br>Who or what technology is present?	When is it happening?<br>Where?<br>Who or what technology is present?
What is happening?	What is happening?	What is happening?	What is happening?	What is happening?

Group ___ , part ___ of ___

User-Journey Storyboard

A user-journey storyboard can be a shortcut to understanding the many different ways different people experience a situation or challenge. A storyboard can quickly capture details that would normally only be surfaced by intense and prolonged shadowing processes. There is no 'right' answer to a storyboard, and by completing multiple user-journeys, we can identify where there are differences, as well as similarities.

1. Start with the 'main event' in the middle box, add detail about what is happening, when it is happening, where it is happening, and who (or what) is present at each stage of the journey.
2. Work both backwards and forwards and add extra sheets to each end if you need more frames.

When is it happening? Where? Who or what technology is present?	When is it happening? Where? Who or what technology is present?
What is happening?	What is happening?

Group , part of

Opportunities

the design clinic

This is ok for now, but could be an opportunity for innovation:	This needs to be improved:	This really needs to be improved:
This is ok for now, but could be an opportunity for innovation:	This needs to be improved:	This really needs to be improved:
This is ok for now, but could be an opportunity for innovation:	This needs to be improved:	This really needs to be improved:
This is ok for now, but could be an opportunity for innovation:	This needs to be improved:	This really needs to be improved:
This is ok in the current system but could be an opportunity for innovation:	This needs to be improved:	This really needs to be improved:
This is ok in the current system but could be an opportunity for innovation:	This needs to be improved:	This really needs to be improved:
This is ok for now, but could be an opportunity for innovation:	This needs to be improved:	This really needs to be improved:
This is ok for now, but could be an opportunity for innovation:	This needs to be improved:	This really needs to be improved:
This is ok for now, but could be an opportunity for innovation:	This needs to be improved:	This really needs to be improved:

When is it happening? Where? Who or what technology is present?

What is happening?

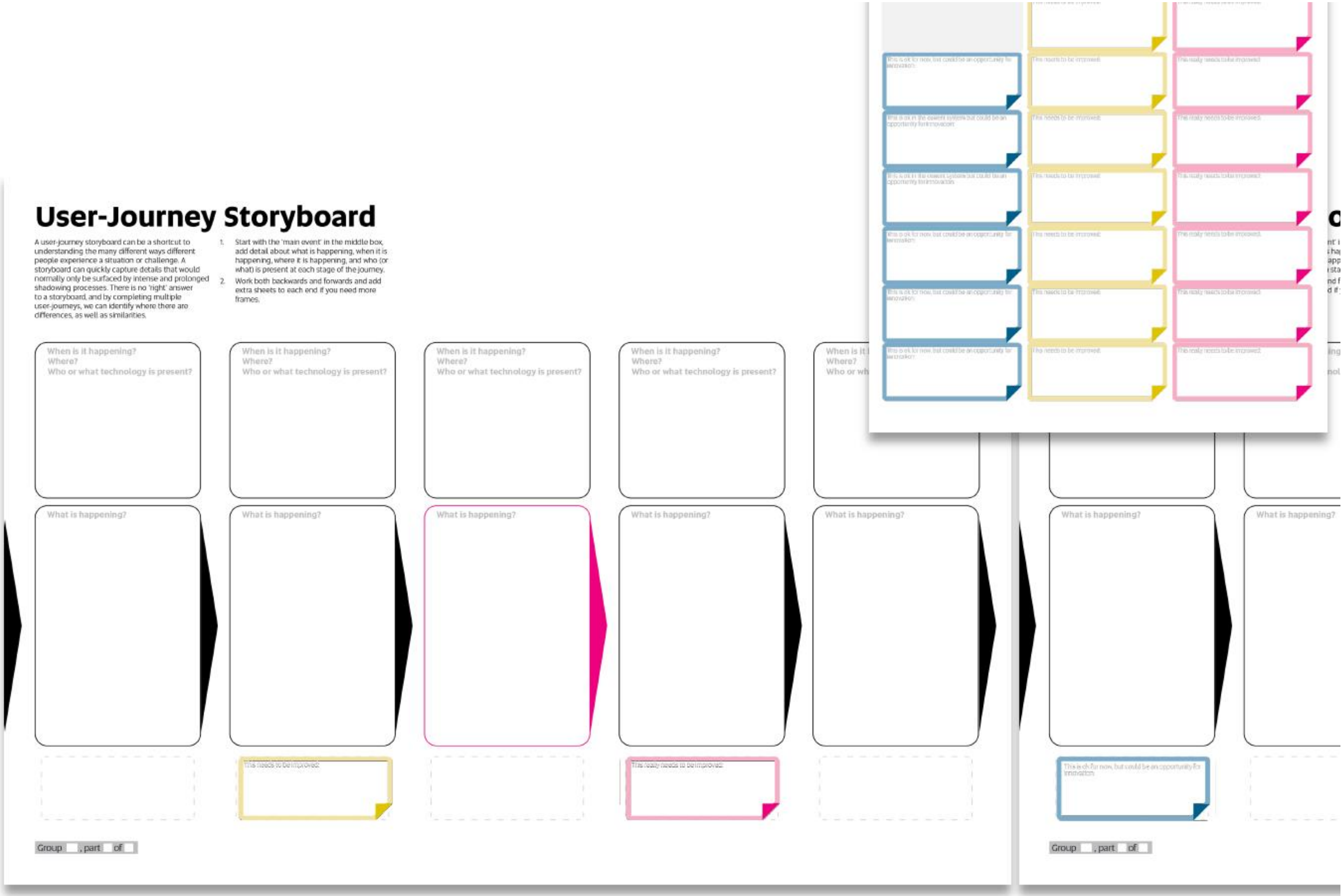

User-Journey Storyboard
A user-journey storyboard can be a shortcut to understanding the many different ways different people experience a situation or challenge. A storyboard can quickly capture details that would normally only be surfaced by intense and prolonged shadowing processes. There is no 'right' answer to a storyboard, and by completing multiple user-journeys, we can identify where there are differences, as well as similarities.
1. Start with the 'main event' in the middle box, add detail about what is happening, when it is happening, where it is happening, and who (or what) is present at each stage of the journey.
2. Work both backwards and forwards and add extra sheets to each end if you need more frames.
When is it happening?
Where?
Who or what technology is present?
What is happening?
This needs to be improved:
This really needs to be improved:
This is ok for now, but could be an opportunity for innovation.
Group , part of
