# Supplementary figures and images for: Stepping toward implementation using co-design: development of hospital protocols and resources for using wearable activity trackers in a hospital service
Source: Front Digit Health. 2025 Mar 18;7:1520991. doi: 10.3389/fdgth.2025.1520991 (PMC11959083; doi:10.3389/fdgth.2025.1520991)

# Wearables in Remote Rehab Empathy Map

Designed by:

Date:

Version:

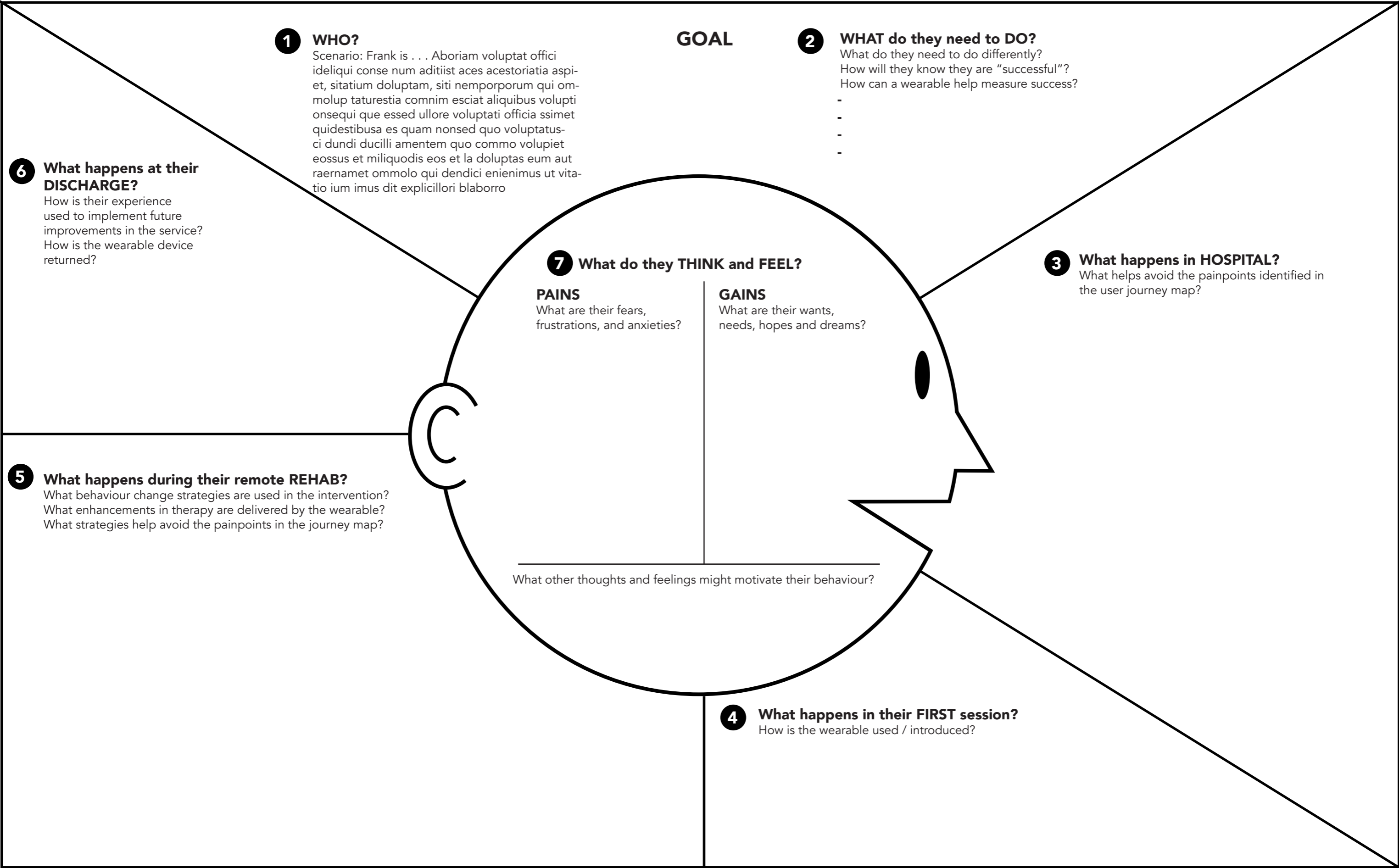

Supplement: Supplementary file 2 [file Datasheet2.pdf]
