# Supplementary material for: Stepping toward implementation using co-design: development of hospital protocols and resources for using wearable activity trackers in a hospital service
Source: Front Digit Health. 2025 Mar 18;7:1520991. doi: 10.3389/fdgth.2025.1520991 (PMC11959083; doi:10.3389/fdgth.2025.1520991)

Station 1: Setting goals and progressing patient activity during admission to the Virtual Rehabilitation Ward using wearable activity trackers

We know: patients benefit from increasing their activity during a hospital admission, and wearable activity trackers can support increases in activity and physical function. Currently there is not a standardized activity target for this setting, but gradual progressions in activity are recommended, and small daily increases in activity (as little as 250 steps) have been linked to better recovery outcomes.

We were told: activity goals set with wearable activity trackers should be meaningful to the patient, achievable, progressive and specific.

We'd like to find out: What is good about, and what are the challenges for, different approaches to setting goals and progressing patient activity using wearable activity trackers during admission to the virtual rehabilitation ward.

Standardized daily targets for every day (or couple of days) of admission that are the <u>same for all patients</u>.

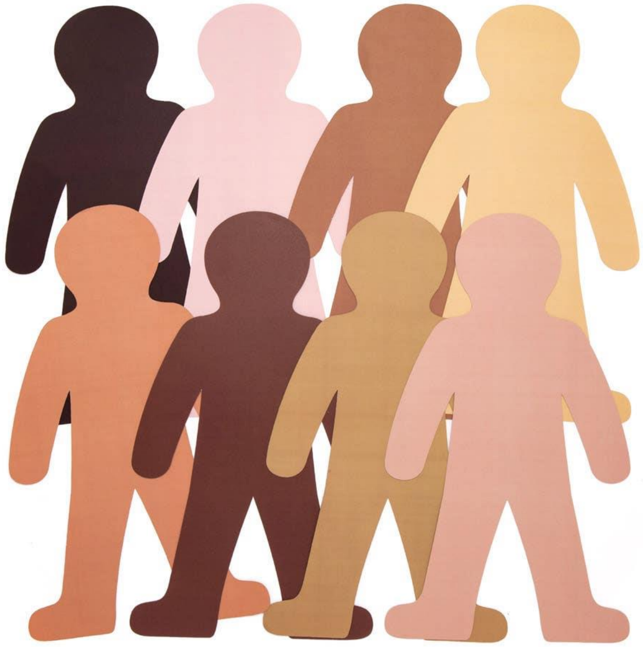

Increase step count by 200 each day	
**Day 2**	200 steps
**Day 3**	400 steps
**Day 4**	600 steps
**Day 5**	800 steps
**Day 6**	1000 steps
**Day 7**	1200 steps
**Day 8**	1400 steps
**Day 9**	1600 steps
**Day 10**	1800 steps

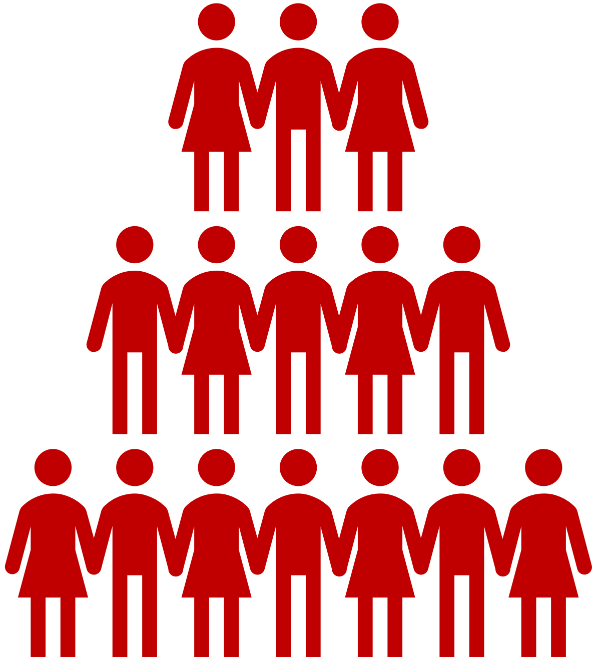

Standardized daily step targets depending on baseline steps

Different activity progression strategy is used based on baseline activity

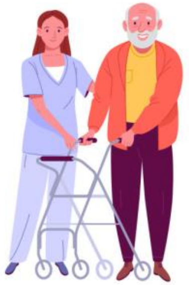

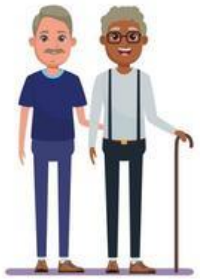

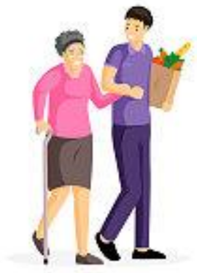

If baseline (day 1) steps <100 (100 step increase daily)	
Day 2	100 steps
Day 3	200 steps
Day 4	300 steps
Day 5	400 steps
Day 6	500 steps
Day 7	600 steps
Day 8	700 steps
Day 9	800 steps
Day 10	900 steps

If baseline (day 1) steps 100-300 (200 step increase daily)	
Day 2	400 steps
Day 3	600 steps
Day 4	800 steps
Day 5	1000 steps
Day 6	1200 steps
Day 7	1400 steps
Day 8	1600 steps
Day 9	1800 steps
Day 10	2000 steps

If baseline (day 1) steps >300 (350 step increase daily	
Day 2	750 steps
Day 3	1100 steps
Day 4	1450 steps
Day 5	1800 steps
Day 6	2150 steps
Day 7	2500 steps
Day 8	2750 steps
Day 9	3100 steps
Day 10	3450 steps

Step goal individualized for each patient, based on a % of previous day

Same % change applies for all patients, specific step target varies based on what they achieve each day

25%

**EXAMPLE 1** With 25% increase each day (assuming each day step goal is met)			
**If baseline (day 1) steps 100**		**If baseline (day 1) steps 250**	
Day 2	125 steps	Day 2	315 steps
Day 3	160 steps	Day 3	395 steps
Day 4	200 steps	Day 4	495 steps
Day 5	250 steps	Day 5	620 steps
Day 6	315 steps	Day 6	775 steps
Day 7	395 steps	Day 7	970 steps
Day 8	495 steps	Day 8	1215 steps
Day 9	620 steps	Day 9	1520 steps
Day 10	775 steps	Day 10	1900 steps

40%

**EXAMPLE 2** With 40% increase each day (assuming each day step goal is met)			
**If baseline (day 1) steps 100**		**If baseline (day 1) steps 250**	
Day 2	140 steps	Day 2	350 steps
Day 3	196 steps	Day 3	490 steps
Day 4	275 steps	Day 4	686 steps
Day 5	384 steps	Day 5	960 steps
Day 6	538 steps	Day 6	1345 steps
Day 7	735 steps	Day 7	1882 steps
Day 8	1054 steps	Day 8	2635 steps
Day 9	1476 steps	Day 9	3689 steps
Day 10	2066 steps	Day 10	5165 steps

50%

**EXAMPLE 3** With 50% increase each day (assuming each day step goal is met)			
**If baseline (day 1) steps 100**		**If baseline (day 1) steps 250**	
Day 2	150 steps	Day 2	375 steps
Day 3	225 steps	Day 3	560 steps
Day 4	340 steps	Day 4	840 steps
Day 5	510 steps	Day 5	1260 steps
Day 6	765 steps	Day 6	1890 steps
Day 7	1150 steps	Day 7	2835 steps
Day 8	1725 steps	Day 8	4250 steps
Day 9	2590 steps	Day 9	6375 steps
Day 10	3885 steps	Day 10	9562 steps

Step goal is completely individualized, based on what the clinician thinks is suitable

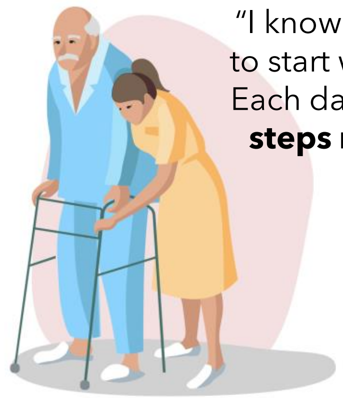

"I know it's been hard to start walking again. Each day, aim for **100 steps more** than the previous day"

**Baseline**	**75 steps**
Day 2	175 steps
Day 3	275 steps
Day 4	375 steps
Day 5	475 steps
Day 6	575 steps
Day 7	675 steps
Day 8	757 steps
Day 9	875 steps
Day 10	975 steps

**Baseline**	**200 steps**
Day 2	400 steps
Day 3	800 steps
Day 4	1600 steps
Day 5	3200 steps
Day 6	6400 steps
Day 7	8000 steps
Day 8	Maintain
Day 9	Maintain
Day 10	Maintain

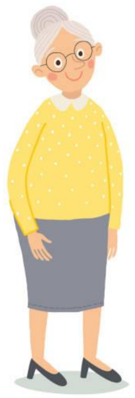

"I'm doing well with my walking and am confident walking alone. Each day I aim to **double my daily step counts** compared to the previous day until I get to 8000"

"You were walking a lot before admission. Let's set a **final goal** of 7000 daily steps at discharge. **Smaller goals until then** can be up to 2000 daily steps in the first week. After that, work toward 3000, then 4000, and then up to 5000."

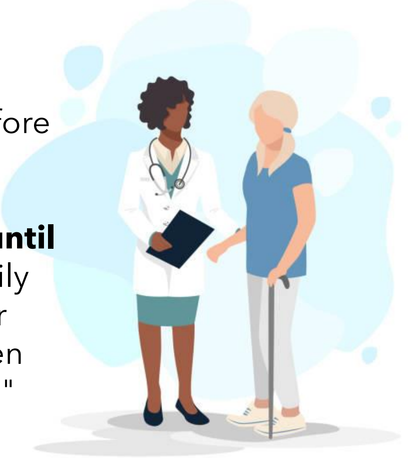

**Baseline**	**300 steps**
Day 2	500 steps
Day 3	800 steps
Day 4	1200 steps
Day 5	1600 steps
Day 6	2000 steps
Day 7	2400 steps
Day 8	2900 steps
Day 9	3400 steps
Day 10	4000 steps

Station 2: How will training and information resources for clinicians be delivered?

We know: It's important that all clinicians know how to use wearable activity trackers with patients in in the Virtual Rehabilitation Ward.

We were told: Training and information should include the rationale, instructions for setting up and using wearable activity trackers, common troubleshooting, and who to contact for further help. We were also told that training and information could take a range of formats.

We'd like to find out: What is good about, and what are the challenges for, different methods of delivering training and information to clinicians

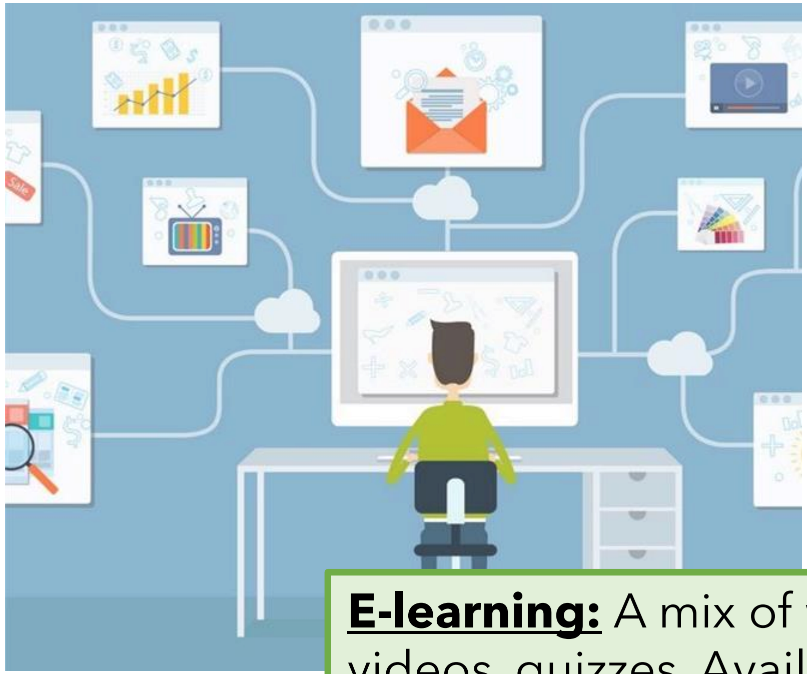

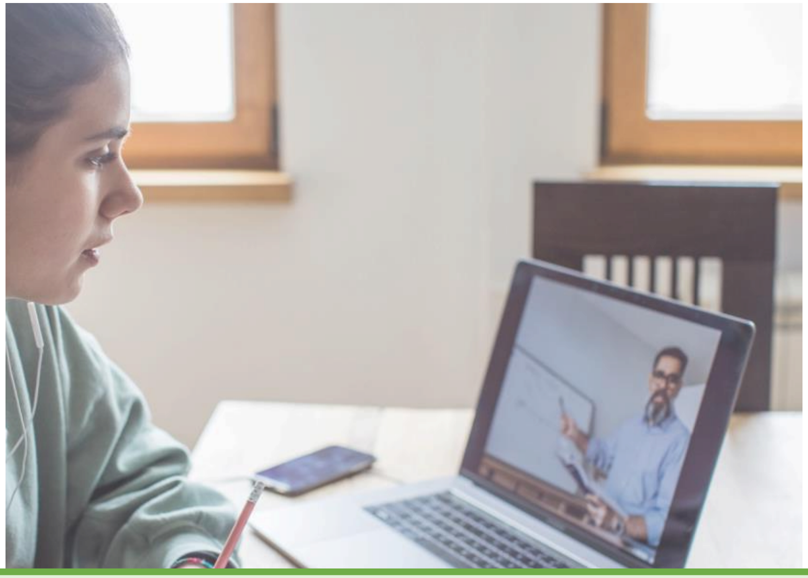

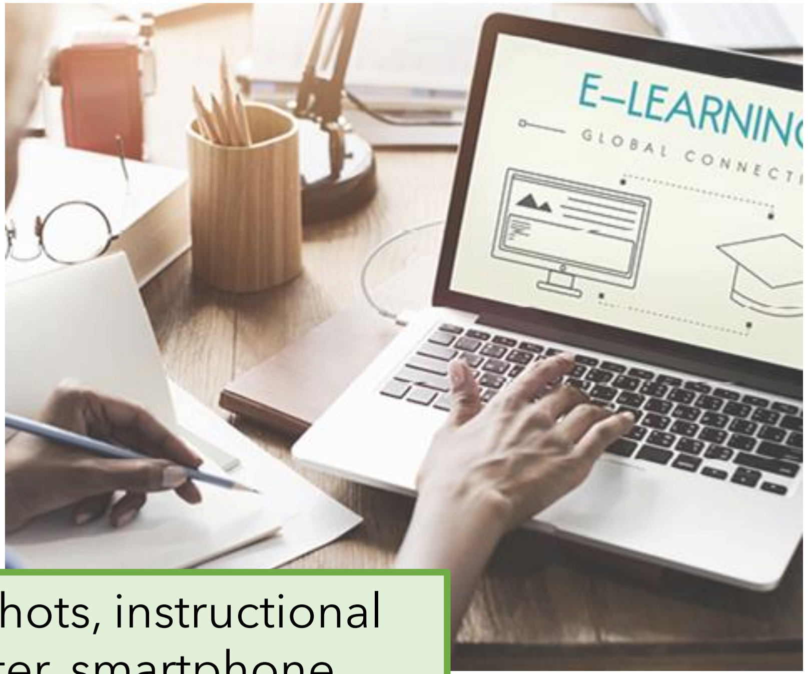

<u>E-learning:</u> A mix of written information, images and screenshots, instructional videos, quizzes. Available on a website accessible via computer, smartphone, tablet. Completed independently at a convenient time, and accessible any time.

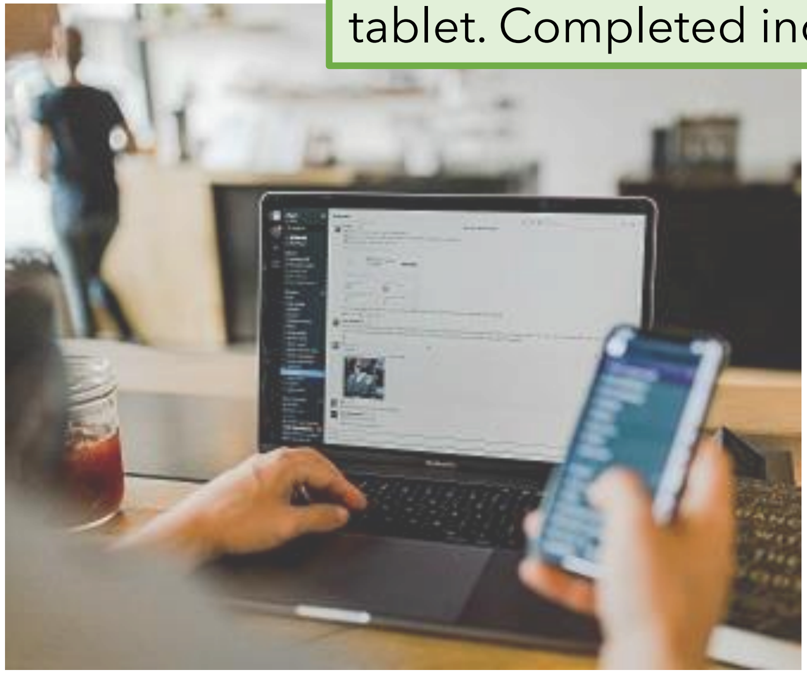

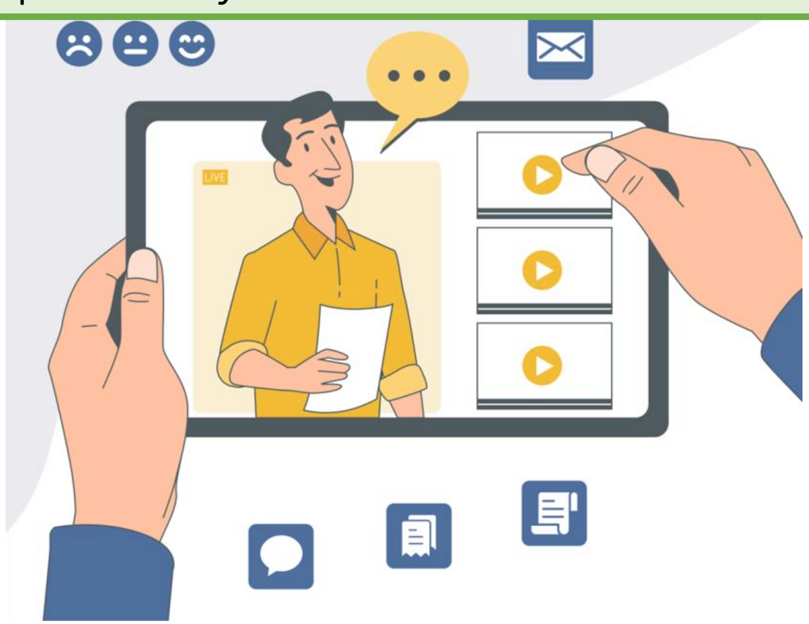

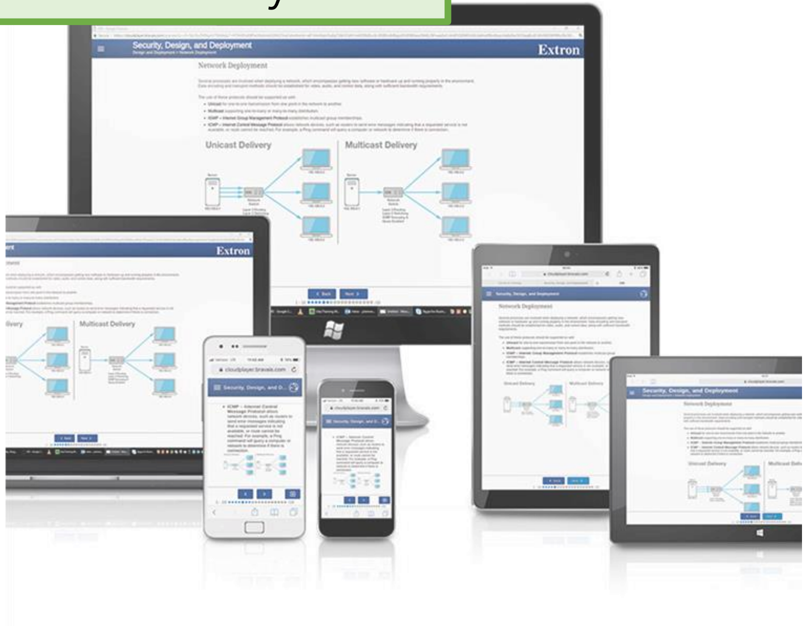

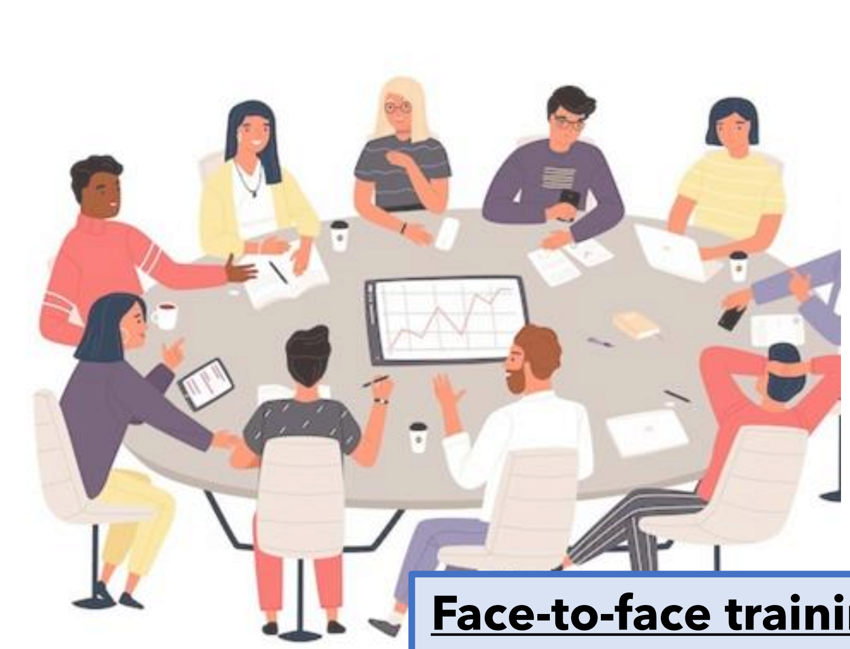

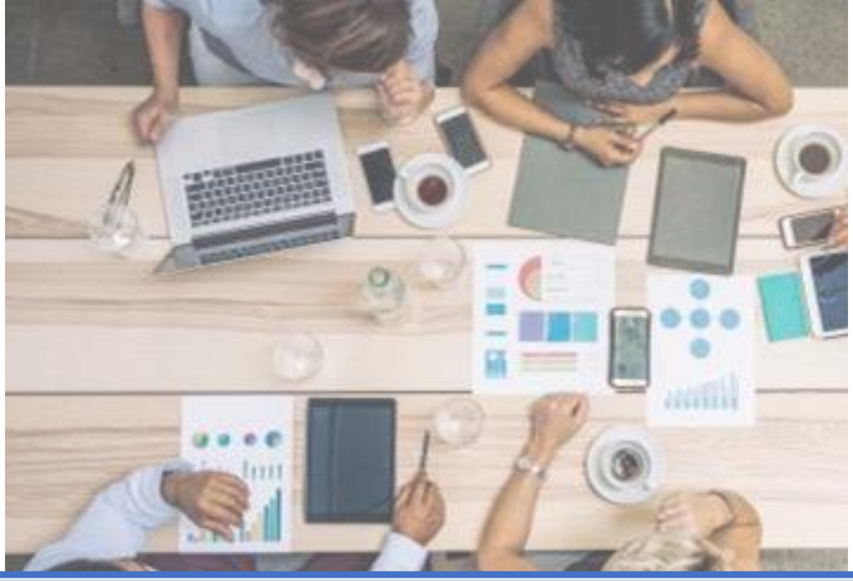

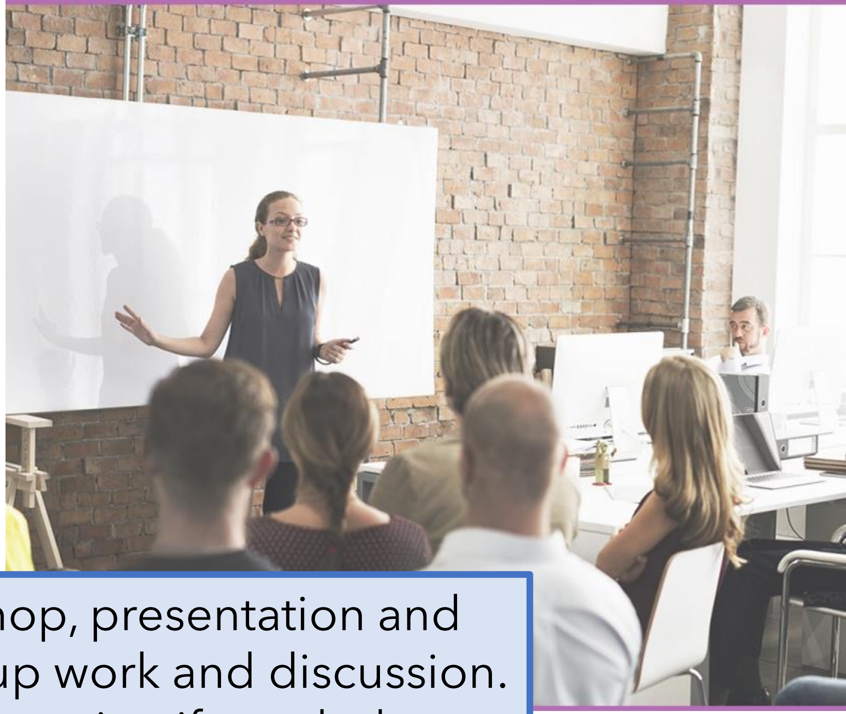

<u>Face-to-face training:</u> instructor-led group training workshop, presentation and practical components, question/answer opportunities, group work and discussion. Competed at a scheduled time before and during implementation if needed.

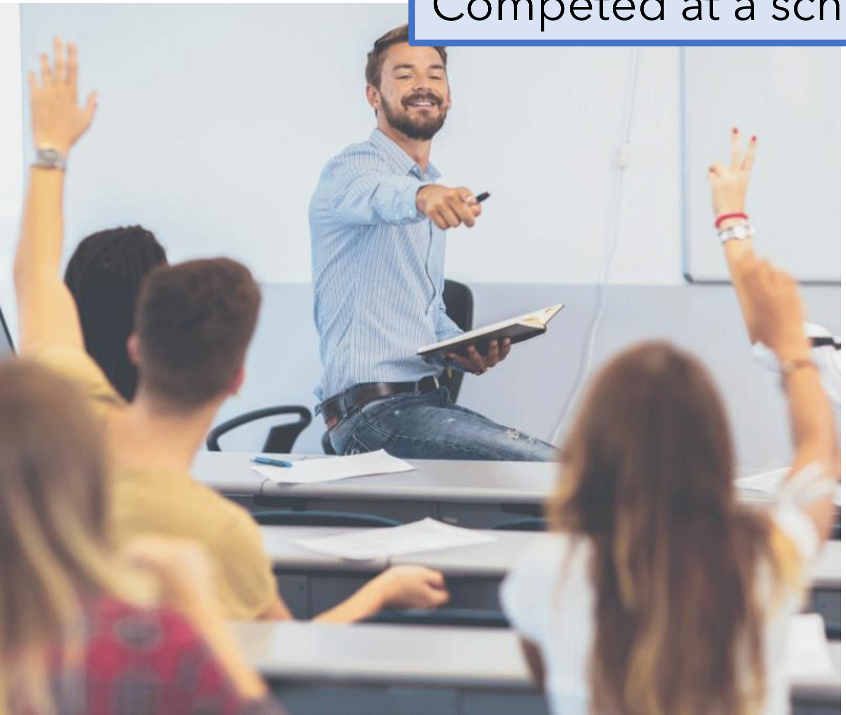

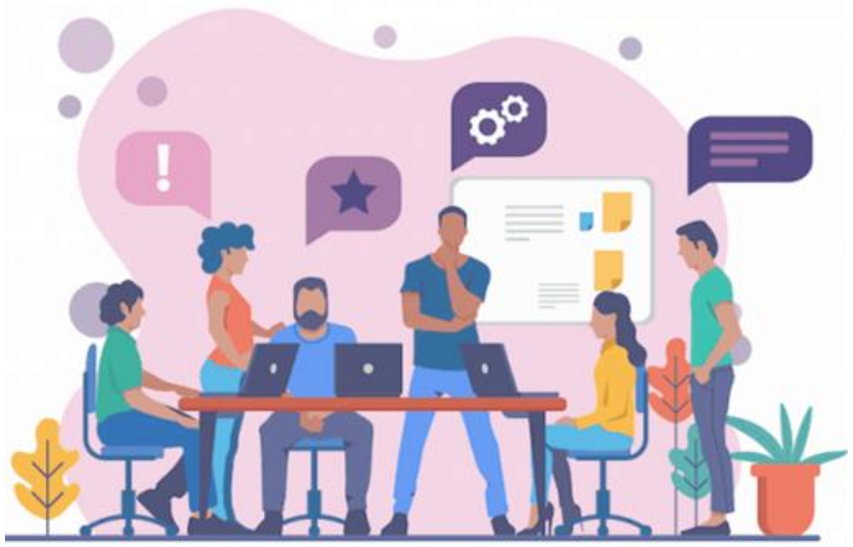

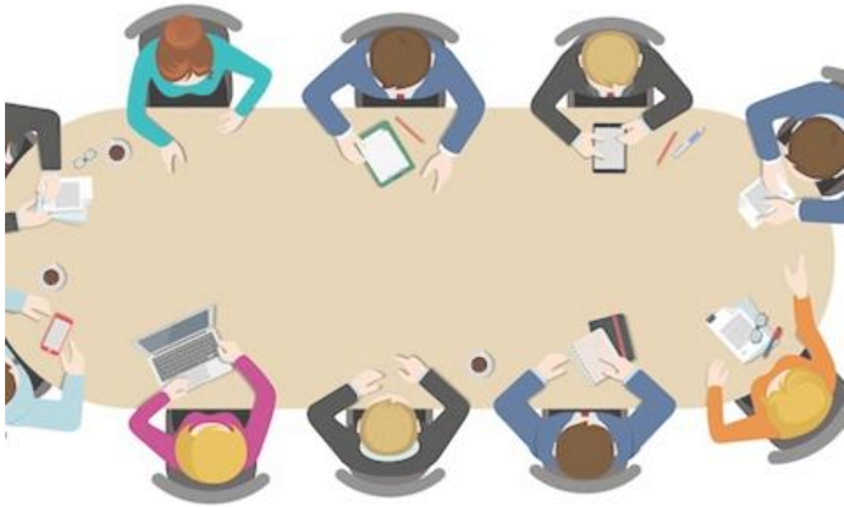

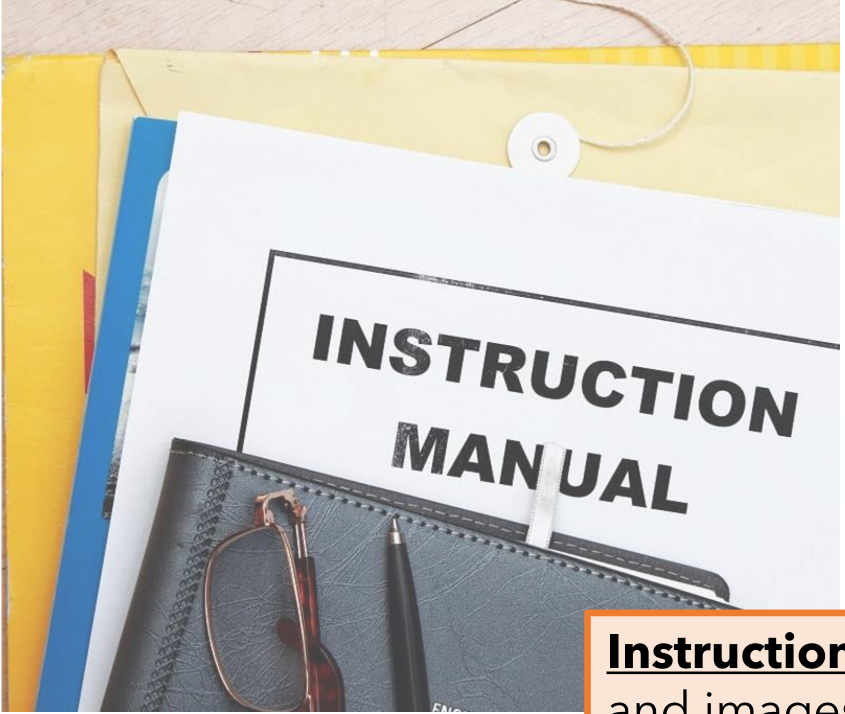

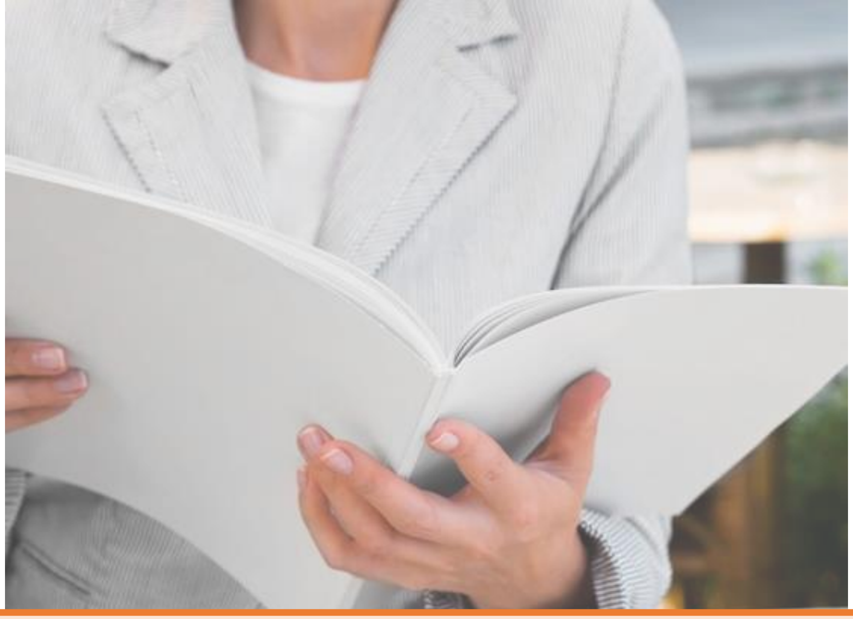

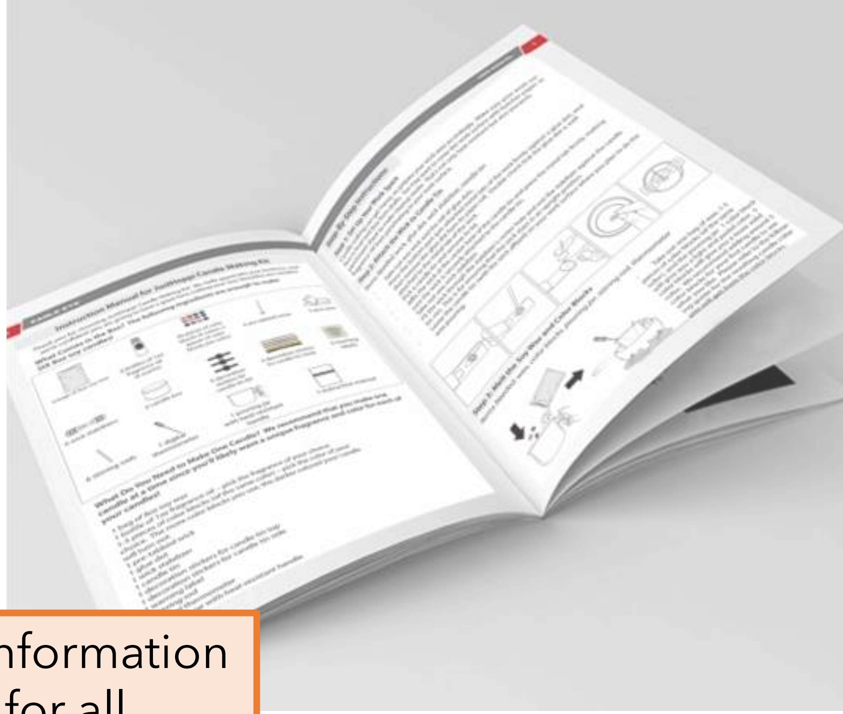

<u>Instruction manual:</u> Physical manual with written information and images. Kept in shared office space accessible for all VRW team members. Clinicians can consult at any time.

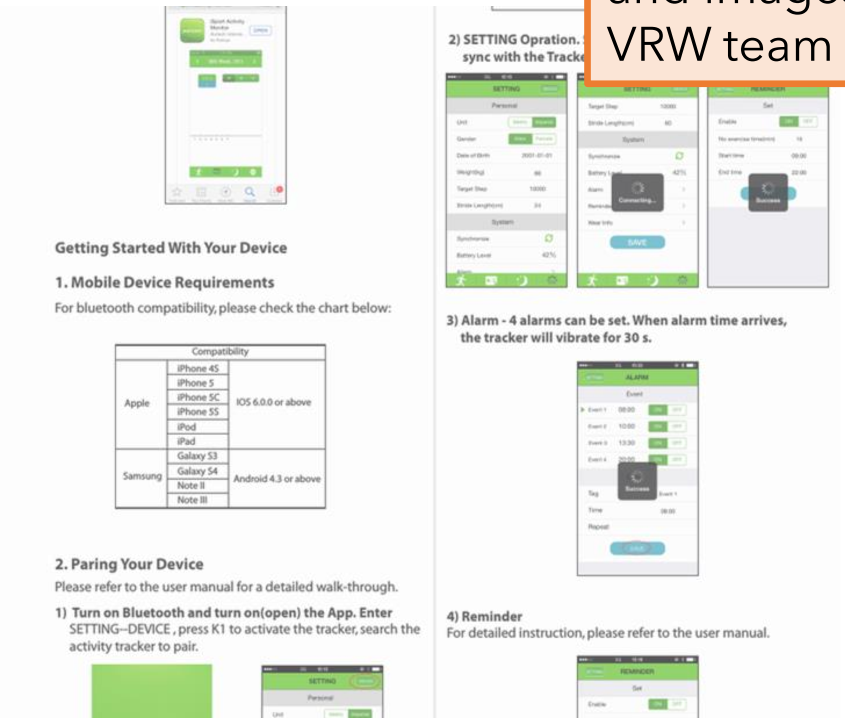

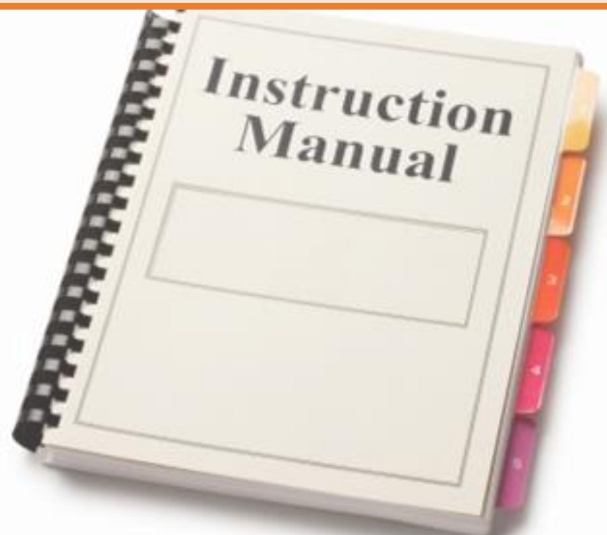

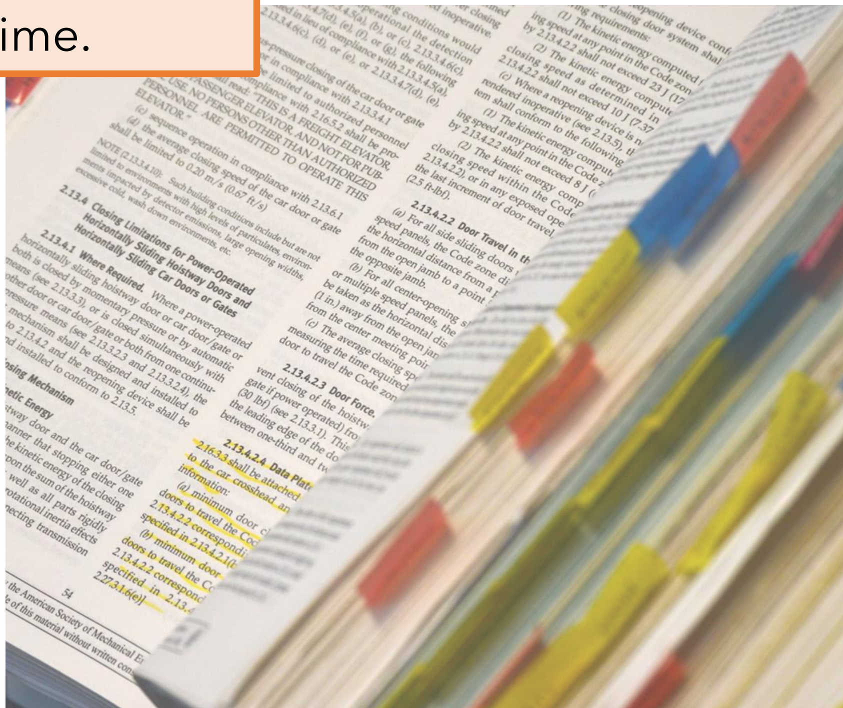

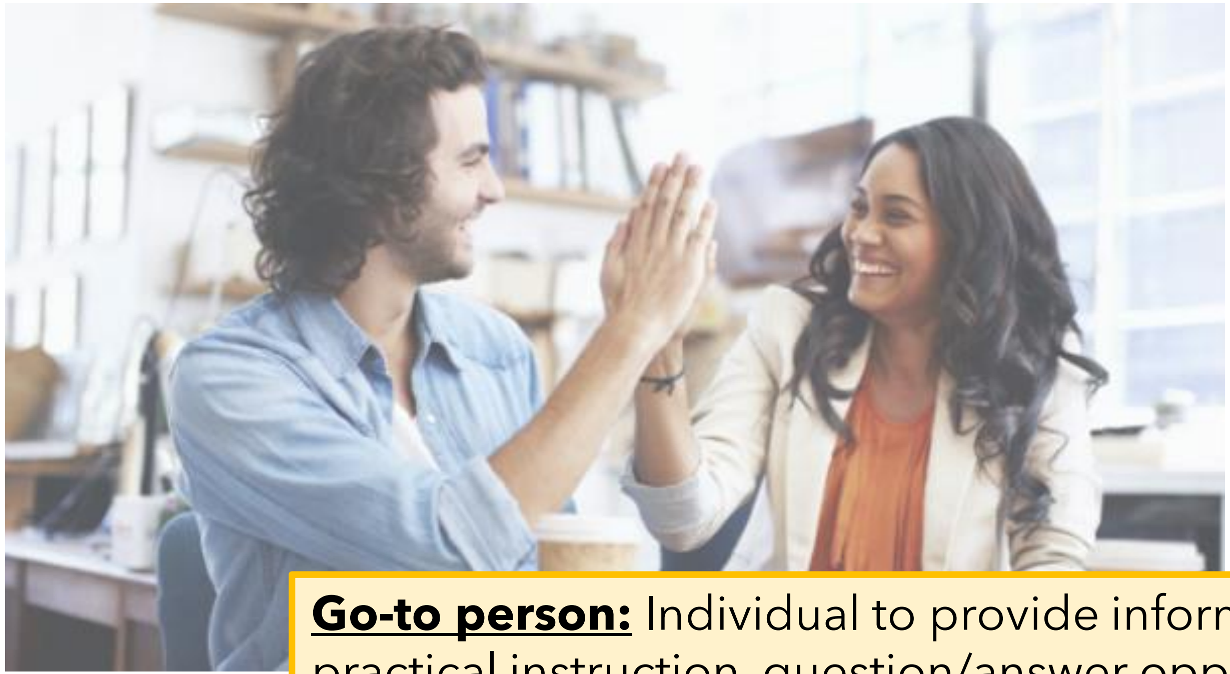
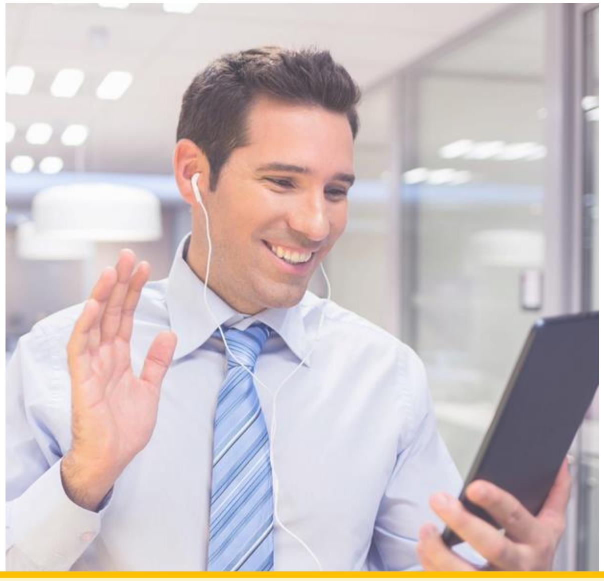
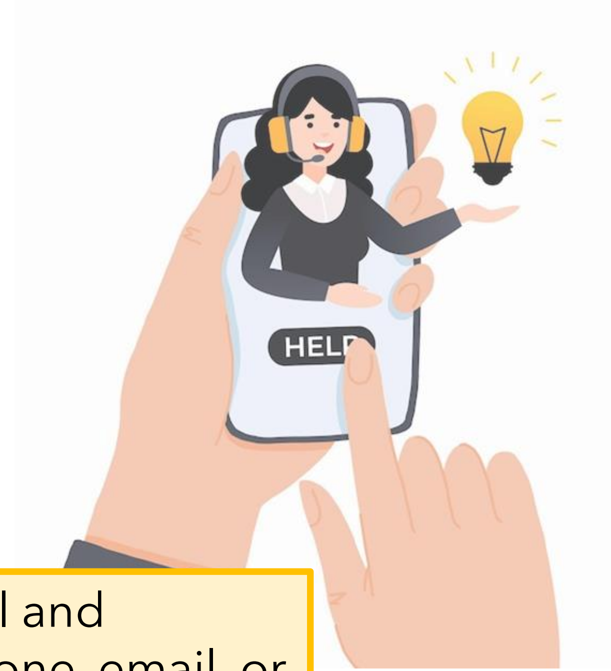

Go-to person: Individual to provide information and 1:1 training. Verbal and practical instruction, question/answer opportunities. Contactable via phone, email, or in-person (by appointment) during work hours before and during implementation.

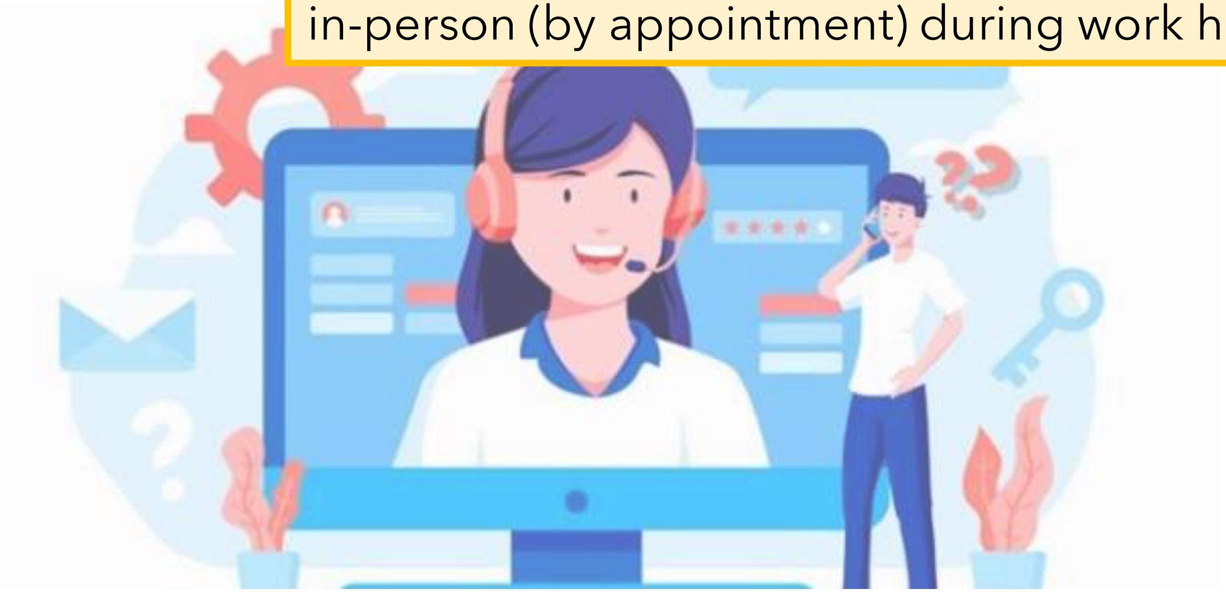
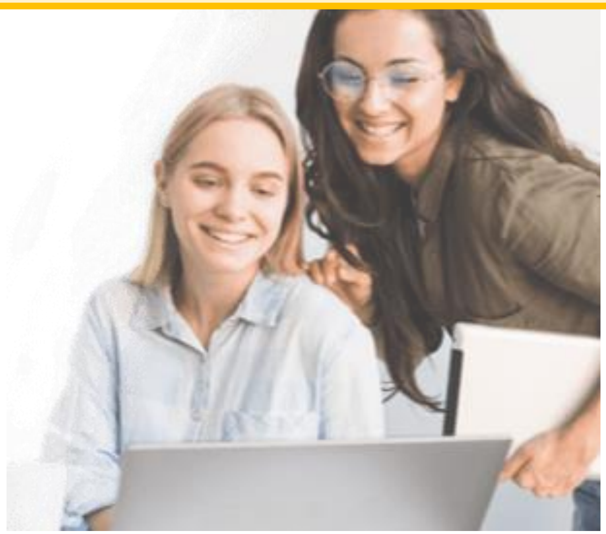
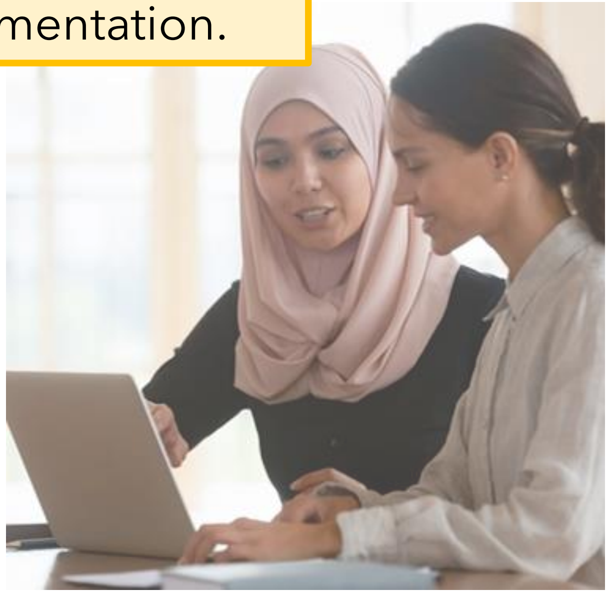

Station 3: How will information and instructions for patients be delivered?

We know: patients need to be provided information and instructions on how to use the wearable activity tracker during their admission.

We heard: patient information should include the rationale, instructions for using wearable activity trackers, and common troubleshooting. We were also told that this information should be presented different formats (including images, written information, verbal instruction, and practical demonstrations), and that involving their family/carers in this can help.

We'd like to find out: What is good about, and what are the challenges for, different formats and strategies of delivering information and instructions to patients.

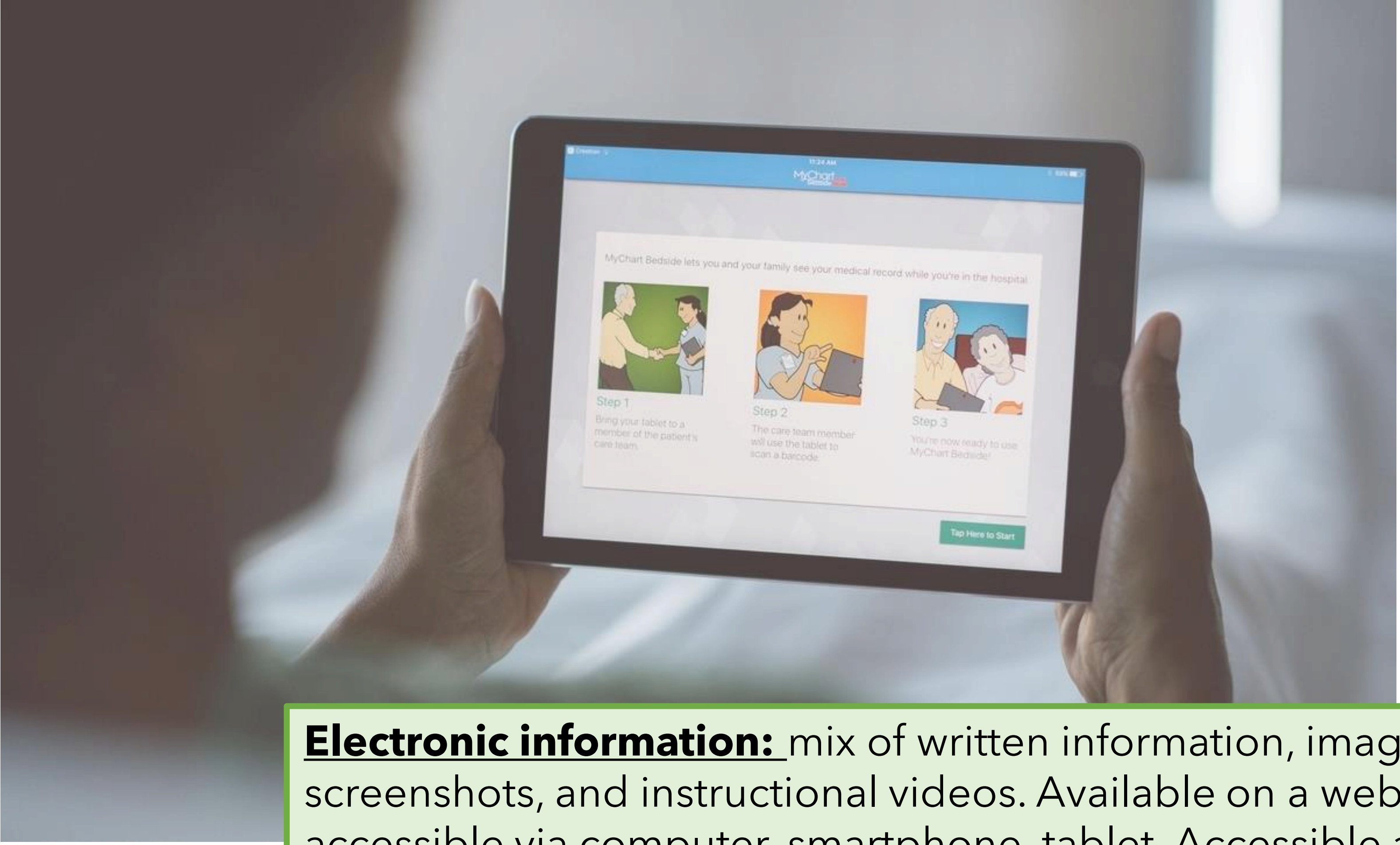

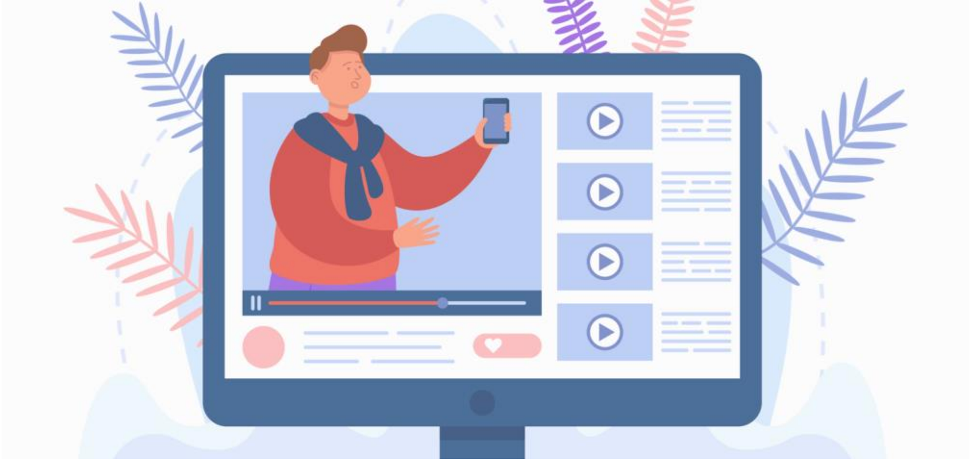

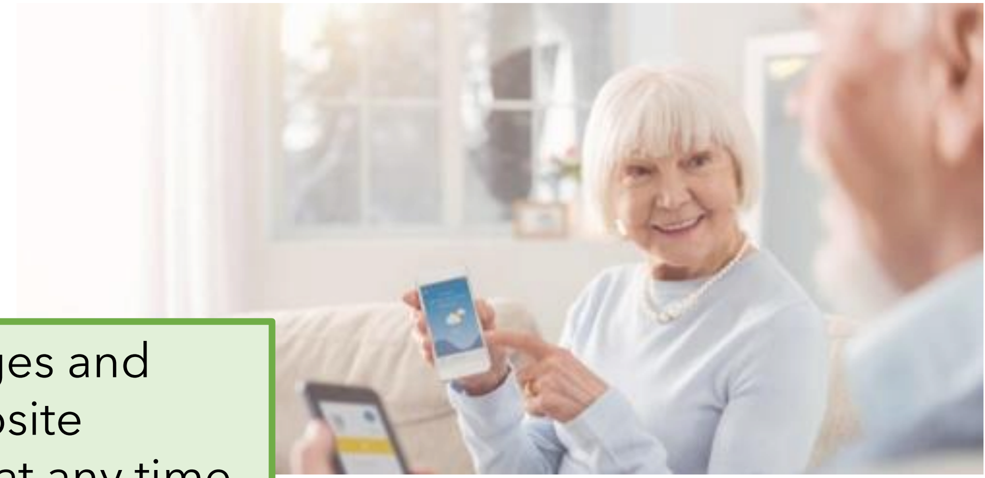

<u>Electronic information:</u> mix of written information, images and screenshots, and instructional videos. Available on a website accessible via computer, smartphone, tablet. Accessible at any time.

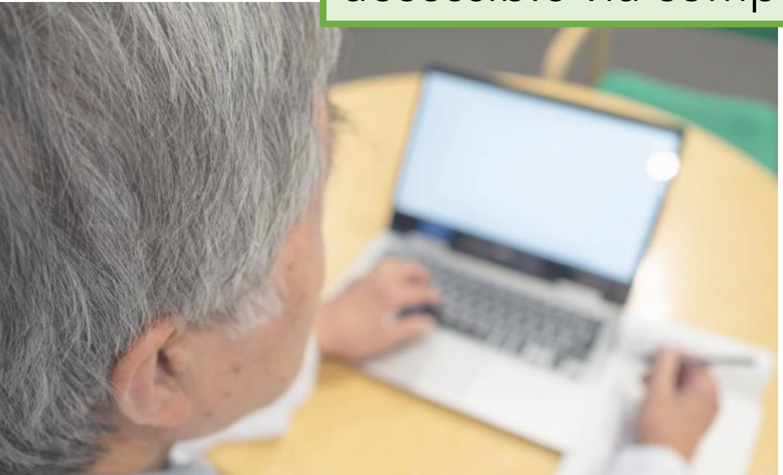

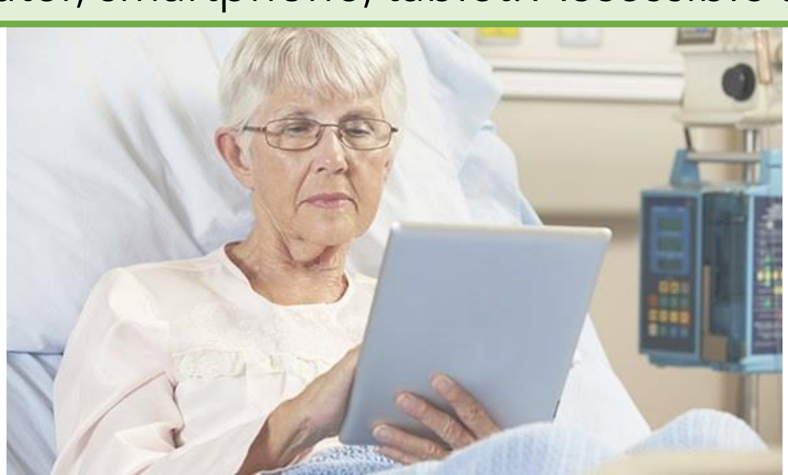

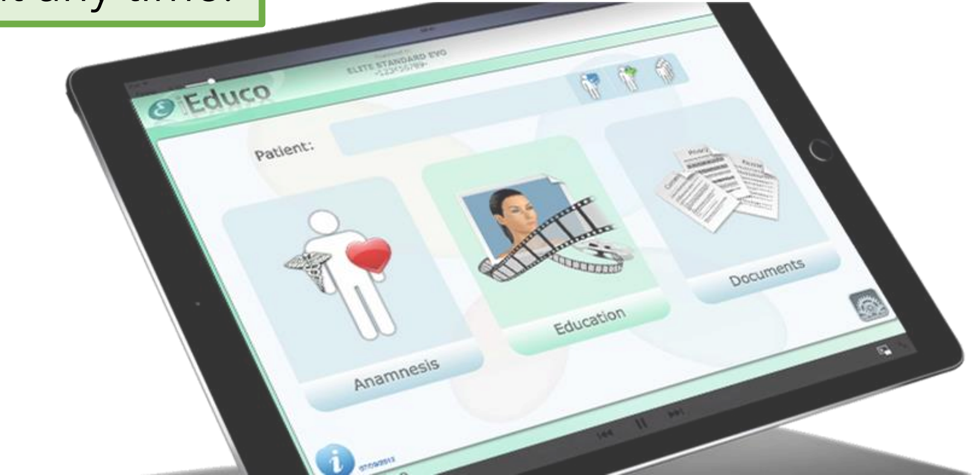

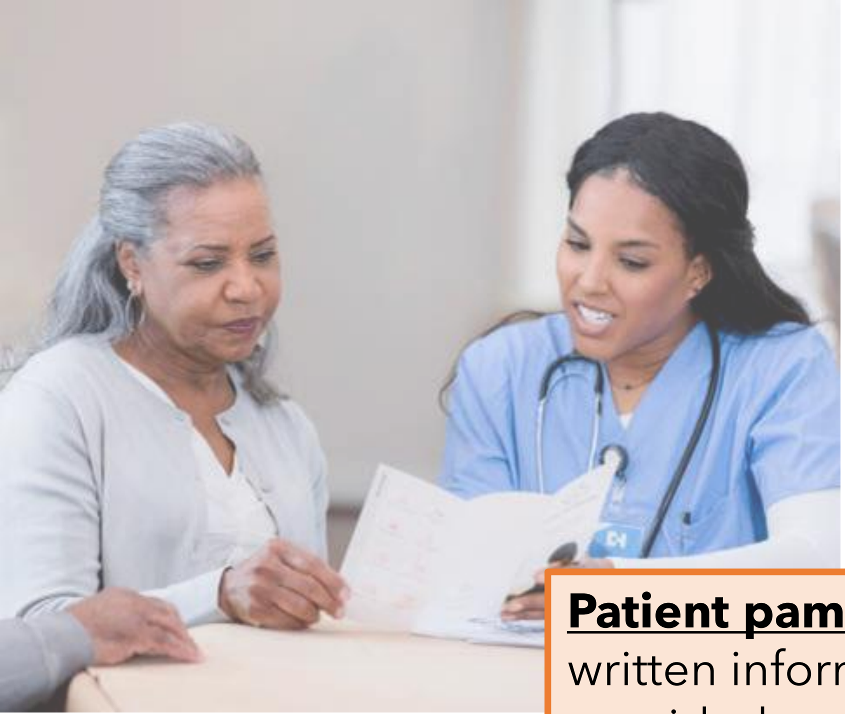

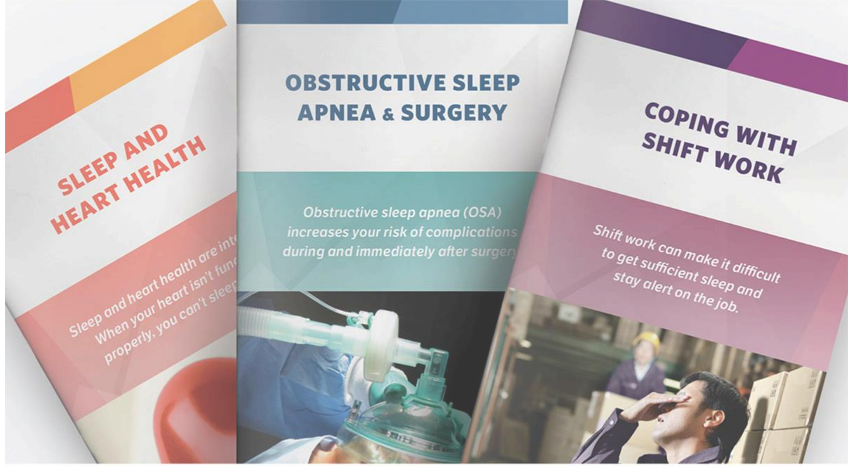

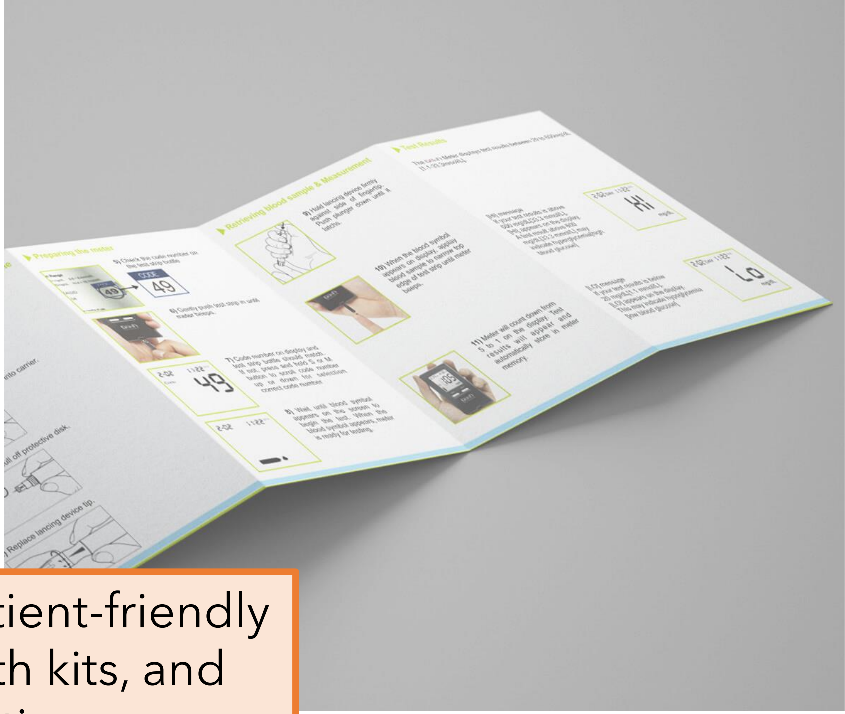

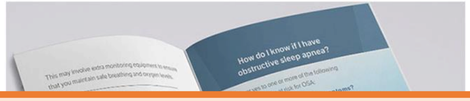

Patient pamphlet: Physical handout with simple, patient-friendly written information/instructions and images. Kept with kits, and provided upon admission. Patient can consult at any time.

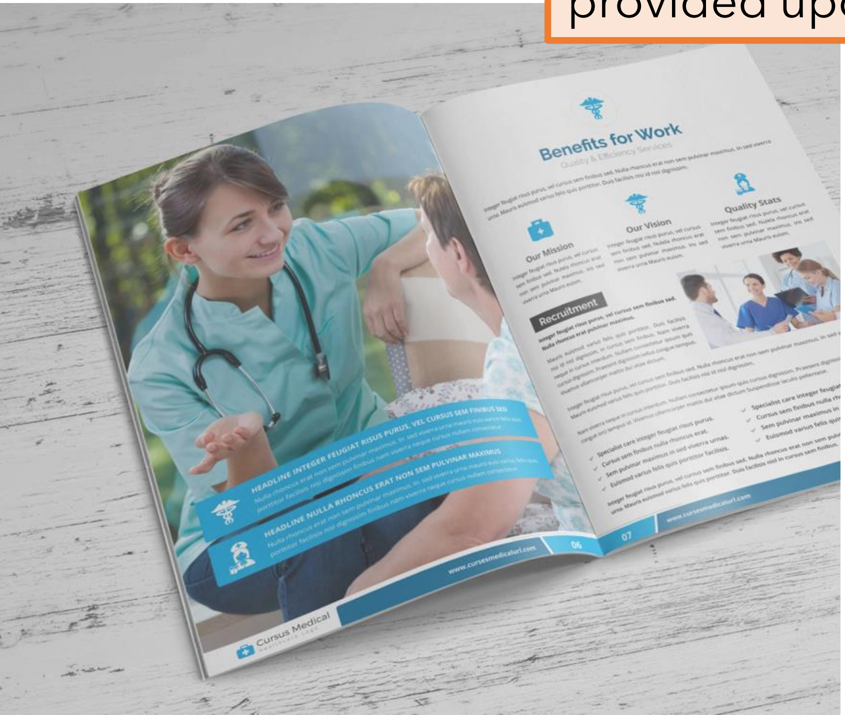

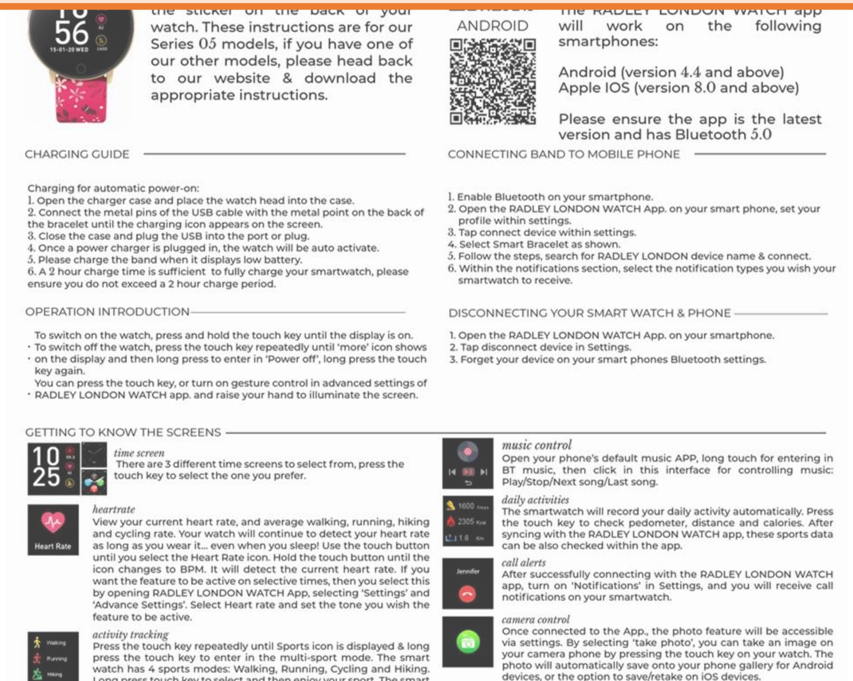
watch. These instructions are for our Series 05 models, if you have one of our other models, please head back to our website & download the appropriate instructions.

ANDROID

will work on the following smartphones:

Android (version 4.4 and above)
Apple IOS (version 8.0 and above)

Please ensure the app is the latest version and has Bluetooth 5.0

CHARGING GUIDE

Charging for automatic power-on:
1. Open the charger case and place the watch head into the case.
2. Connect the metal pins of the USB cable with the metal point on the back of the bracelet until the charging icon appears on the screen.
3. Close the case and plug the USB into the port or plug.
4. Once a power charger is plugged in, the watch will be auto activate.
5. Please charge the band when it displays low battery.
6. A 2 hour charge time is sufficient to fully charge your smartwatch, please ensure you do not exceed a 2 hour charge period.

CONNECTING BAND TO MOBILE PHONE

1. Enable Bluetooth on your smartphone.
2. Open the RADLEY LONDON WATCH App. on your smart phone, set your profile within settings.
3. Tap connect device within settings.
4. Select Smart Bracelet as shown.
5. Follow the steps, search for RADLEY LONDON device name & connect.
6. Within the notifications section, select the notification types you wish your smartwatch to receive.

OPERATION INTRODUCTION

To switch on the watch, press and hold the touch key until the display is on.
- To switch off the watch, press the touch key repeatedly until 'more' icon shows on the display and then long press to enter in 'Power off', long press the touch key again.
- You can press the touch key, or turn on gesture control in advanced settings of RADLEY LONDON WATCH app. and raise your hand to illuminate the screen.

DISCONNECTING YOUR SMART WATCH & PHONE

1. Open the RADLEY LONDON WATCH App. on your smartphone.
2. Tap disconnect device in Settings.
3. Forget your device on your smart phones Bluetooth settings.

GETTING TO KNOW THE SCREENS

time screen
There are 3 different time screens to select from, press the touch key to select the one you prefer.

heartrate
View your current heart rate, and average walking, running, hiking and cycling rate. Your watch will continue to detect your heart rate as long as you wear it... even when you sleep! Use the touch button until you select the Heart Rate icon. Hold the touch button until the icon changes to BPM. It will detect the current heart rate. If you want the feature to be active on selective times, then you select this by opening RADLEY LONDON WATCH App, selecting 'Settings' and 'Advance Settings'. Select Heart rate and set the tone you wish the feature to be active.

activity tracking
Press the touch key repeatedly until Sports icon is displayed & long press the touch key to enter in the multi-sport mode. The smart watch has 4 sports modes: Walking, Running, Cycling and Hiking.

music control
Open your phone's default music APP, long touch for entering in BT music, then click in this interface for controlling music: Play/Stop/Next song/Last song.

daily activities
The smartwatch will record your daily activity automatically. Press the touch key to check pedometer, distance and calories. After syncing with the RADLEY LONDON WATCH app, these sports data can be also checked within the app.

call alerts
After successfully connecting with the RADLEY LONDON WATCH app, turn on 'Notifications' in Settings, and you will receive call notifications on your smartwatch.

camera control
Once connected to the App., the photo feature will be accessible via settings. By selecting 'take photo', you can take an image on your camera phone by pressing the touch key on your watch. The photo will automatically save onto your phone gallery for Android devices, or the option to save/retake on iOS devices.

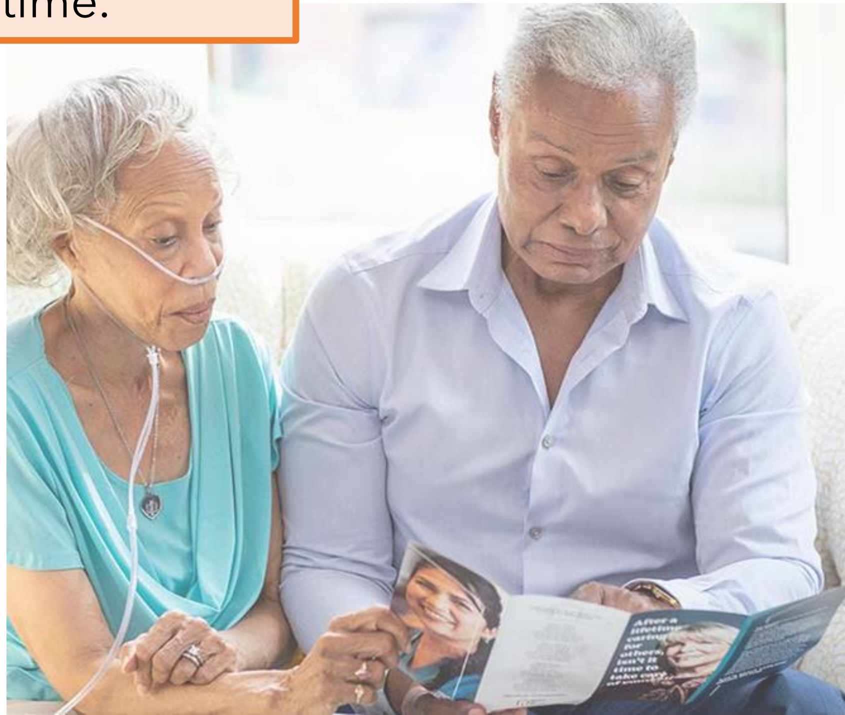

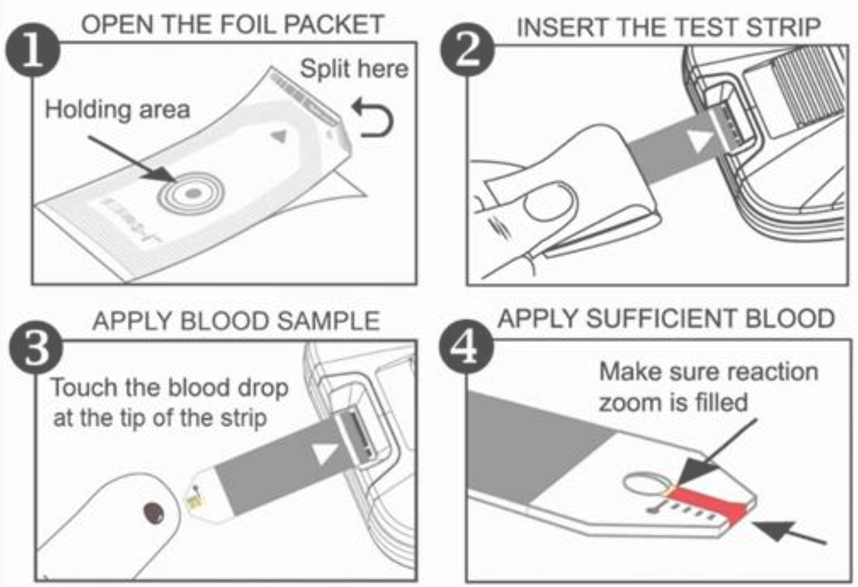

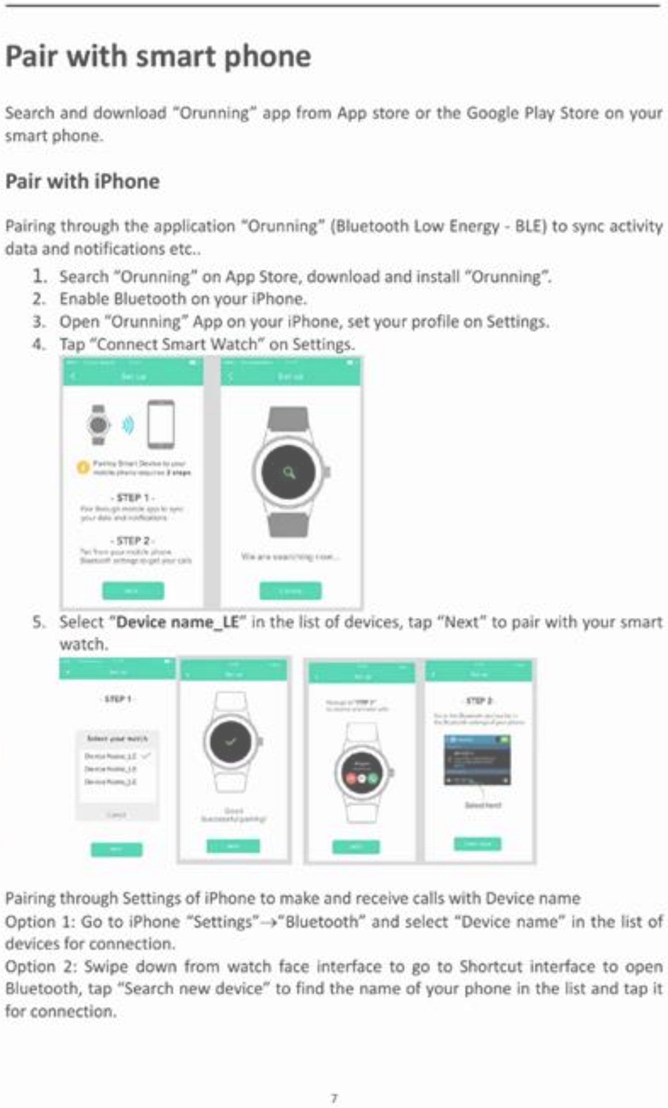

Pair with smart phone

Search and download "Orunning" app from App store or the Google Play Store on your smart phone.

Pair with iPhone

Pairing through the application "Orunning" (Bluetooth Low Energy - BLE) to sync activity data and notifications etc..

1. Search "Orunning" on App Store, download and install "Orunning".
2. Enable Bluetooth on your iPhone.
3. Open "Orunning" App on your iPhone, set your profile on Settings.
4. Tap "Connect Smart Watch" on Settings.
5. Select "**Device name_LE**" in the list of devices, tap "Next" to pair with your smart watch.

Pairing through Settings of iPhone to make and receive calls with Device name

Option 1: Go to iPhone "Settings"→"Bluetooth" and select "Device name" in the list of devices for connection.

Option 2: Swipe down from watch face interface to go to Shortcut interface to open Bluetooth, tap "Search new device" to find the name of your phone in the list and tap it for connection.

7

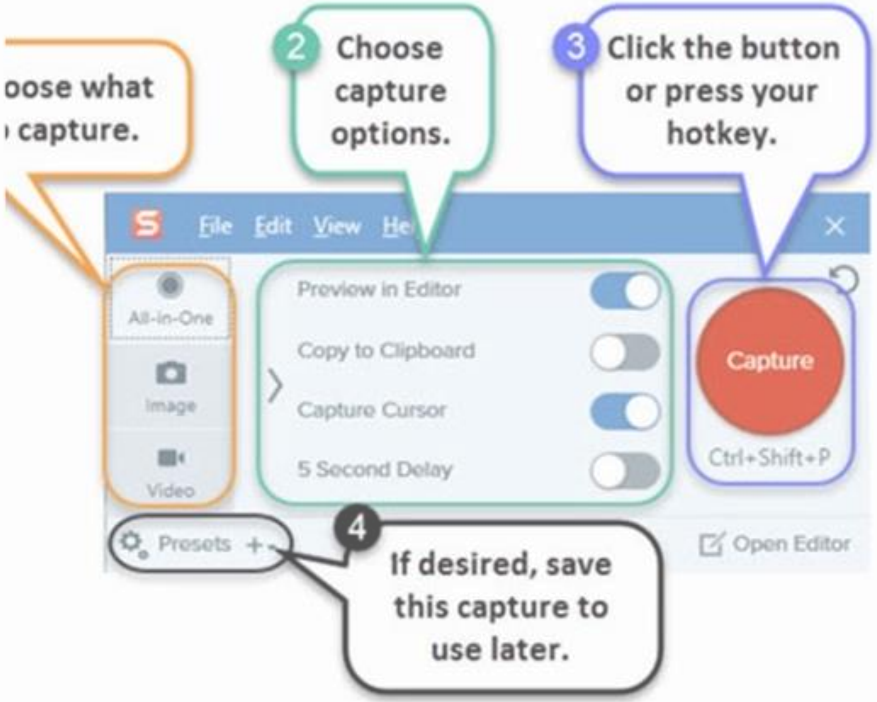

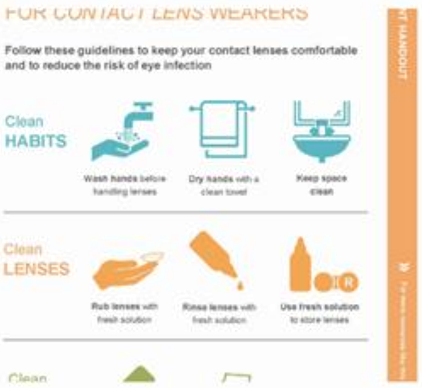

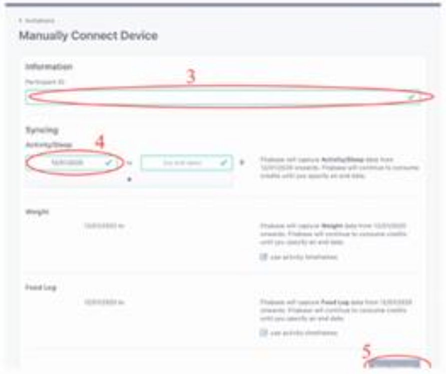

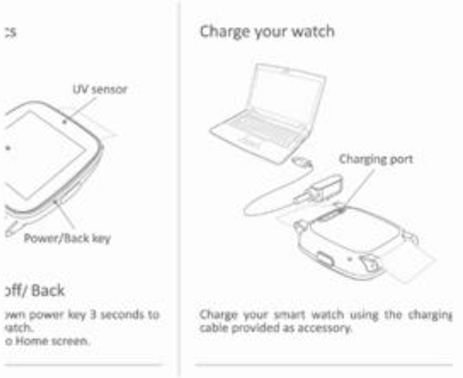

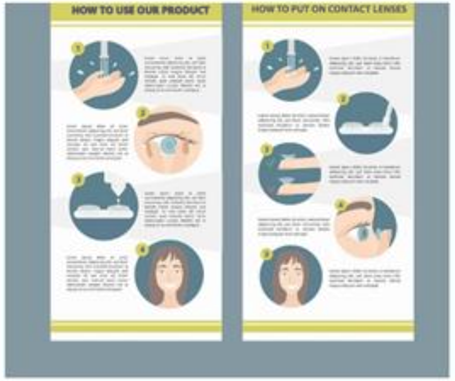

Screenshots and images: Use of images, screenshots and visual information to provide instructions for patients. Colour, shapes, numbers and arrows used to provide direction and differentiate steps. Incorporated into handouts, website etc.

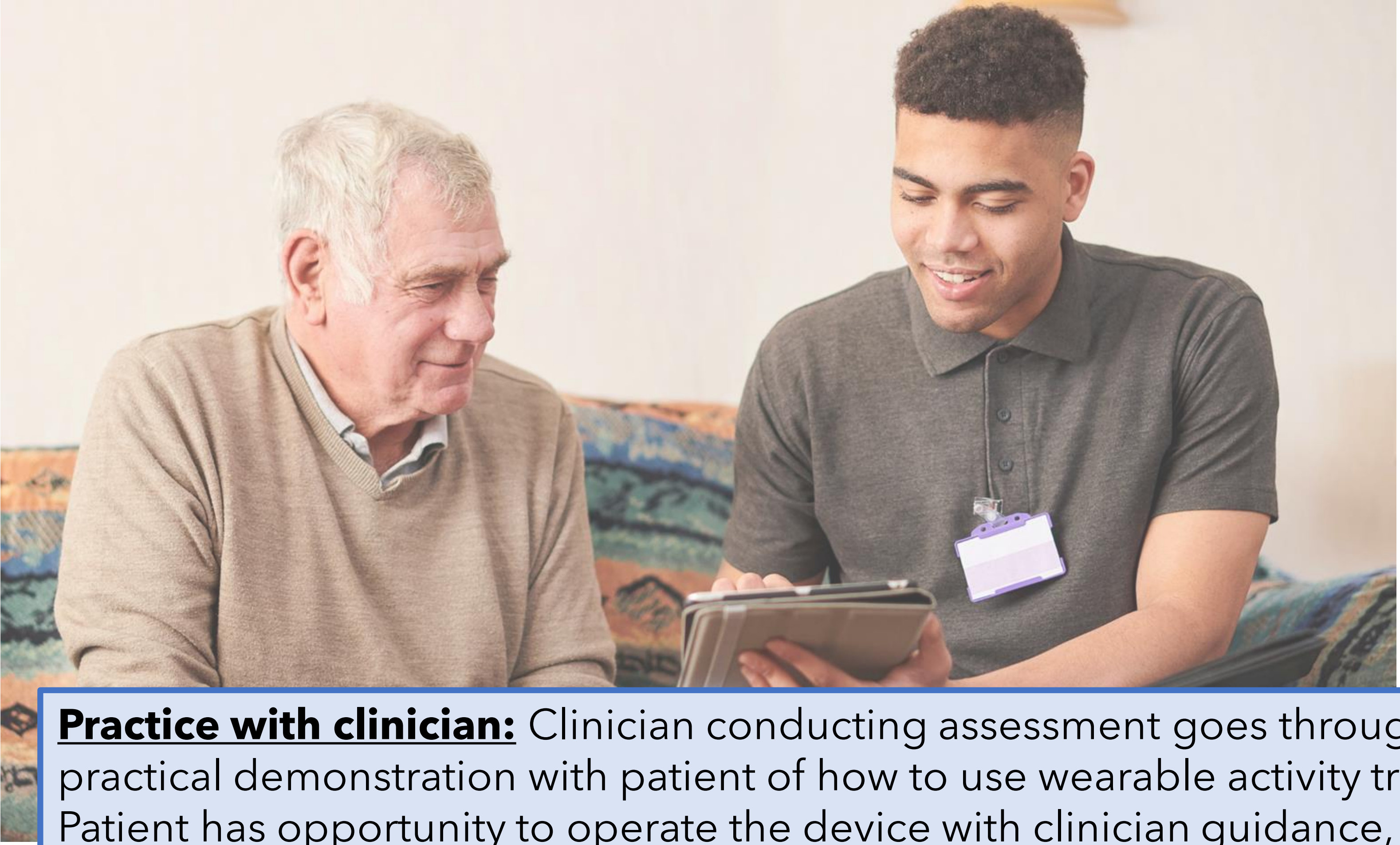
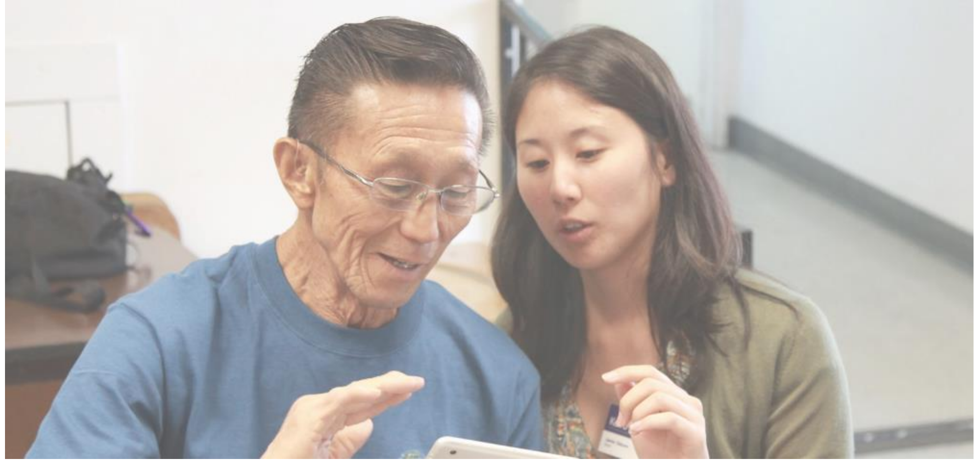
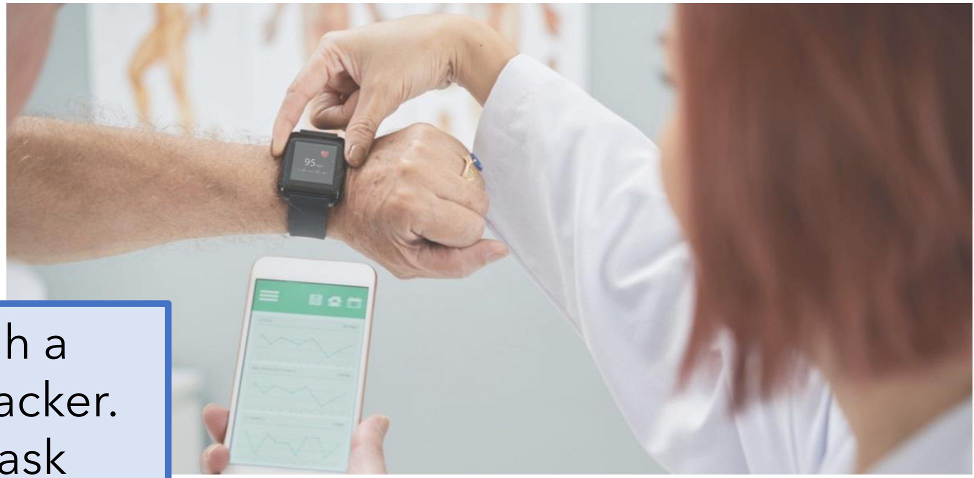

<u>Practice with clinician:</u> Clinician conducting assessment goes through a practical demonstration with patient of how to use wearable activity tracker. Patient has opportunity to operate the device with clinician guidance, ask questions, and receive encouragement and reinforcement from clinician.

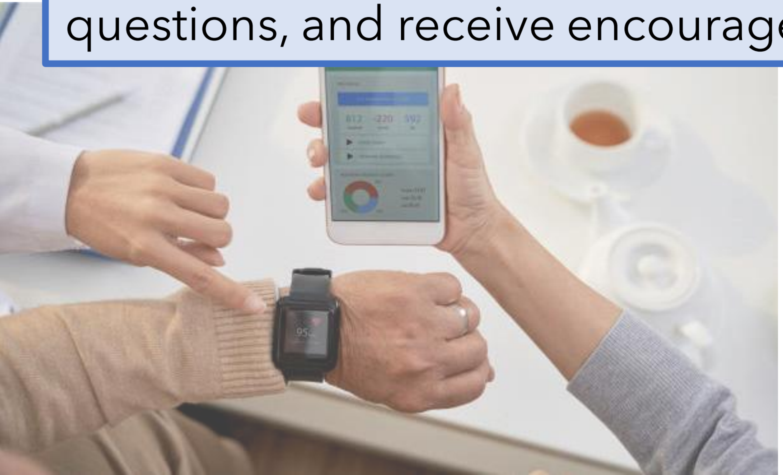
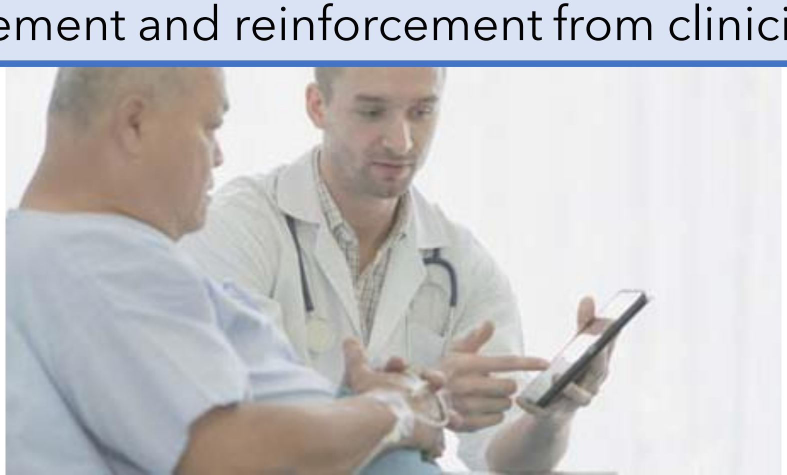
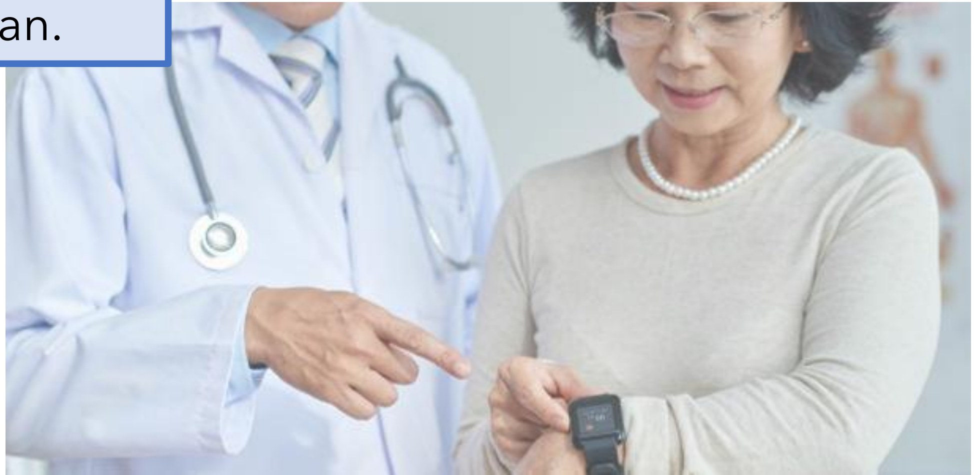

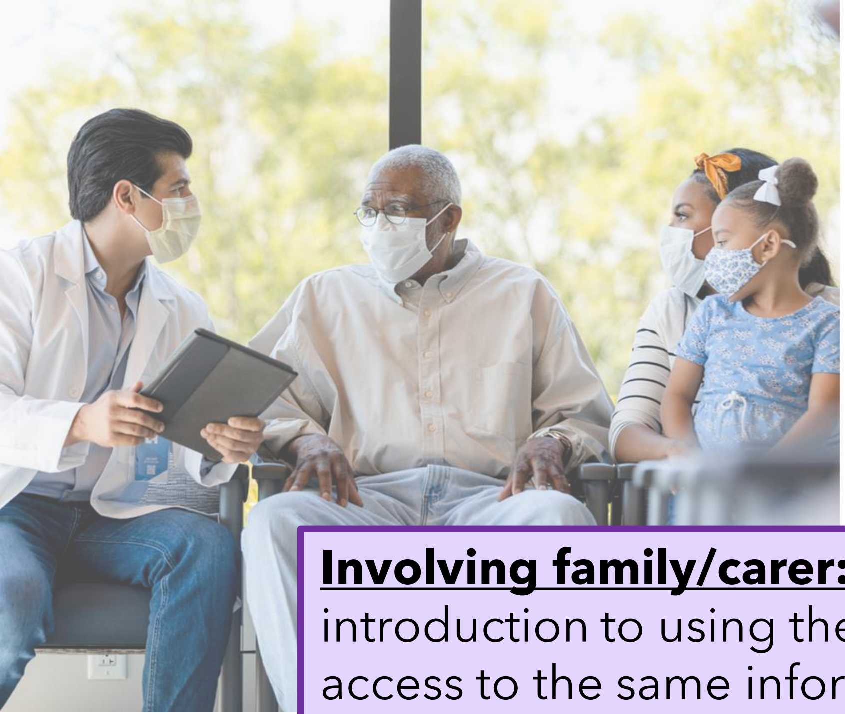

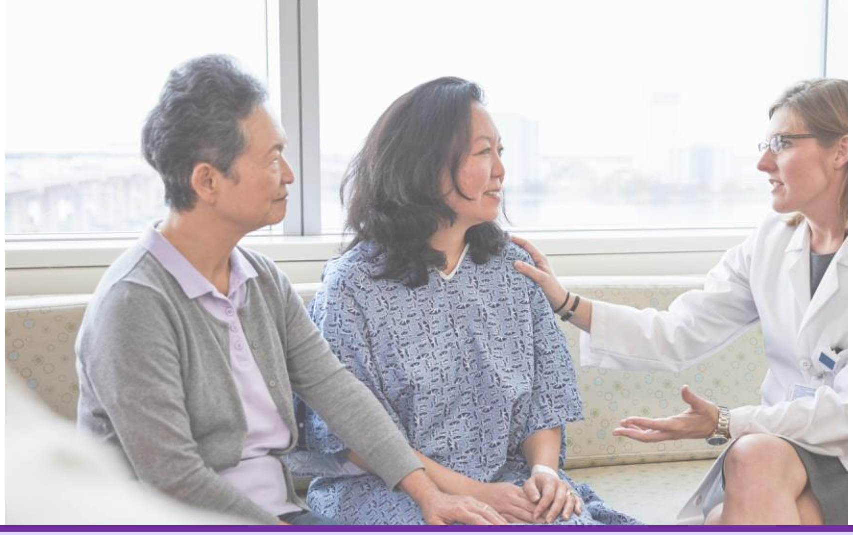

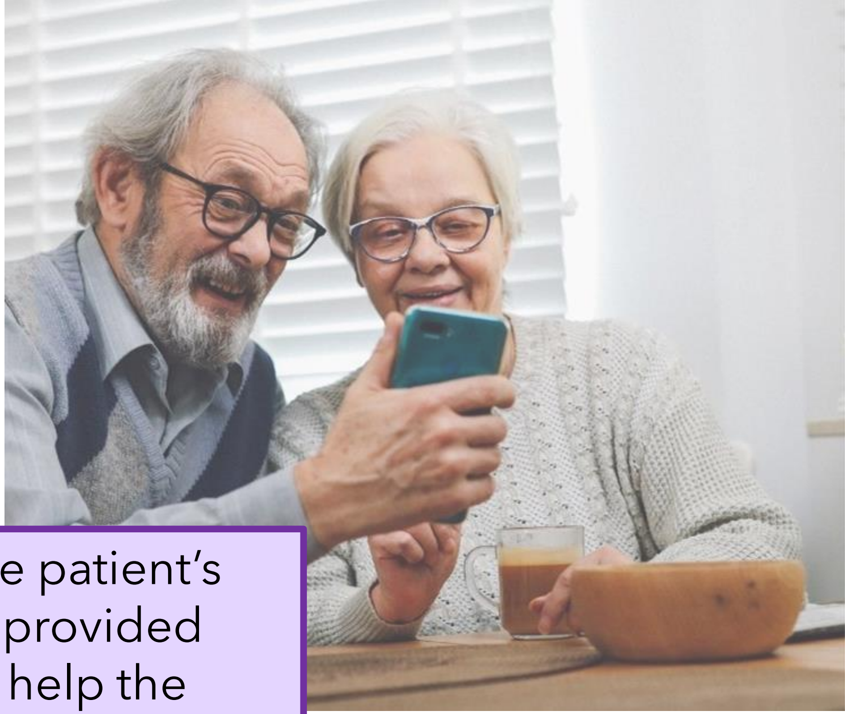

<u>Involving family/carer:</u> A family member/carer is involved in the patient’s introduction to using the wearable activity tracker. They are also provided access to the same information/instructions, and encouraged to help the patient with the device and promote their activity throughout rehabilitation.

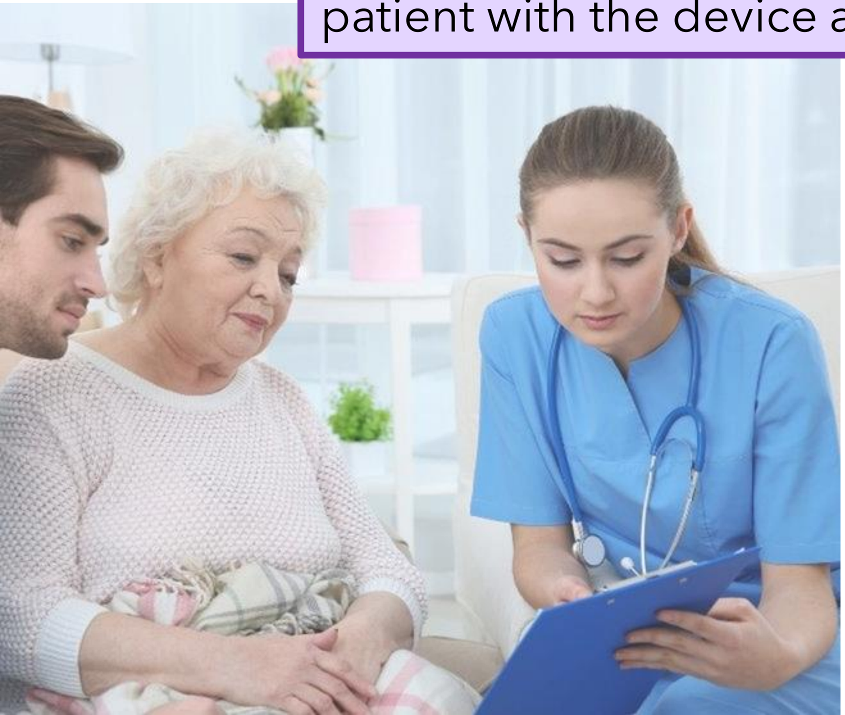

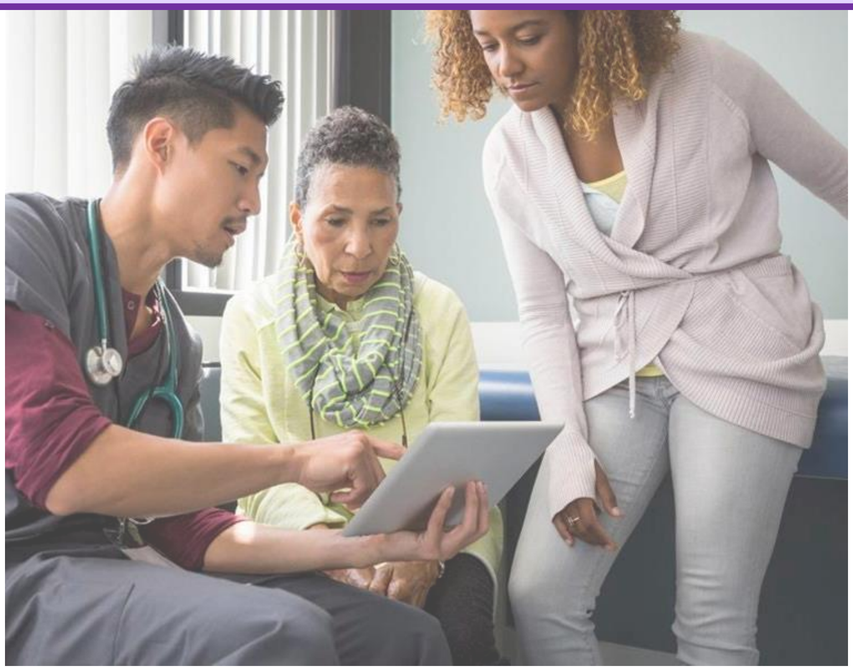

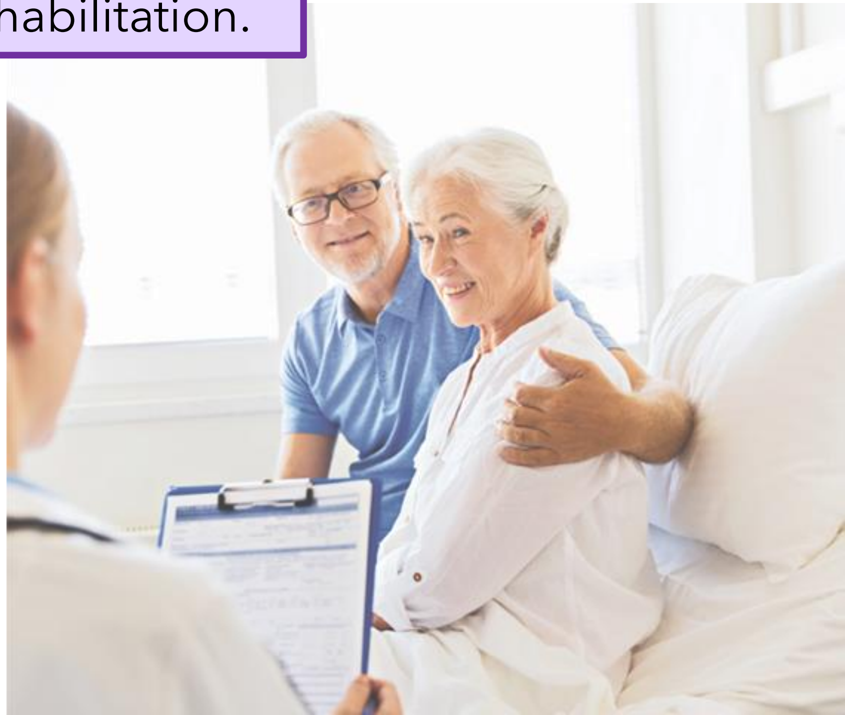

Station 4: Which wearable activity tracker best suits the needs of the Virtual Rehabilitation Ward?

We know: A range of wearable activity trackers with capabilities of measuring and providing feedback on activity metrics are available, and the wearable device should meet the needs of the setting they are being used in. We also know ankle worn devices, despite their challenges, provide much more accurate data.

We were told: The device needs to be patient-friendly (simple), be compatible with the existing technology ecosystem, allow for simple data access, allow remote-viewing of patient data (from hospital), accurately measure activity, be water resistant and easy to clean, and be easy to charge. We were also told there is a strong preference for wrist-worn devices among patients.

What we did: We looked at a range of wearable activity trackers with capabilities of measuring and providing feedback on activity metrics. We rated the different devices based on a range of criteria to identify which specific device was likely most suitable for the Virtual Rehabilitation Ward. The Fitbit Inspire 3 seems to be the most suitable overall, and will best suit the needs of the VRW compared to other devices. But a few details regarding body wear-location need refining.

We'd like to find out: (1) Are there any ratings that you think need to be reviewed? (2) What do you think the benefits and challenges are of using a wrist-worn vs. ankle-worn wearable activity tracker with patients in virtual rehabilitation?

We also want you to vote: What do you think is the best approach to the wear location of the wearable activity tracker for the pilot trial? (1) wrist worn, (2) ankle worn, (3) both (to inform the best wear location for use beyond the pilot trial)

	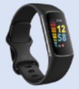 Fitbit Charge 5	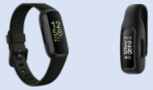 Fitbit Inspire 3	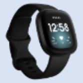 Fitbit Versa 3	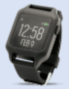 ActiGraph Centre Point Insight Watch	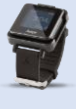 Actigraph GT9X Link	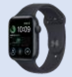 Apple Watch SE series	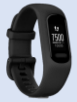 Garmin Vivosmart 5	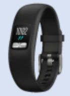 Garmin vivofit 4	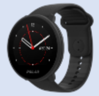 Polar Unite
**Cost**	? AUD$269.95	✓ AUD$179.95	? AUD$299.95	✗ Similar to ActigraphGT9X Link	✗ Approx. AUD$790 (USD$527) Additional cost for charger, straps etc.	✗ AUD$479.00	? AUD$249.00	✓ AUD$139.00	✓ AUD$199.99
**Alternate wear location**	✗ No	✓ Yes. Clip can be purchased for A$29.95	✗ No	✗ No	✓ Wrist, waist, ankle, or thigh with additional equipment	✗ No	✗ No	✗ No	✗ No
**Wrist worn accuracy**	✗ No	? Steps: moderate correlation but **not accurate** in post-cardiac surgery population	? Unable to find	? Unable to find	? Unable to find	? Unable to find	? Unable to find	✗ No	? Unable to find
**Ankle worn accuracy**	✗ Can't be worn on ankle	✓ Step count accurate in inpatient rehab populations, slow walkers, older adults	✗ Can't be worn on ankle	✗ Can't be worn on ankle	✓ Step count accurate in hospitalised critically ill patients	✗ Can't be worn on ankle	✗ Can't be worn on ankle	✗ Can't be worn on ankle	✗ Can't be worn on ankle
**Compatibility with iPad**	✓ Yes. Fitbit app is compatible with only an iPad	✓ Yes. Fitbit app is compatible with only an iPad	✓ Yes. Fitbit app is compatible with only an iPad	✓ Yes, with CentrePoint software	✓ Yes, with CentrePoint software	✗ Only if using an iPhone too.	? Unclear if compatible without smartphone	? Unclear if compatible without smartphone	✓ Yes. Polar Unit app is compatible with iPad
**Battery life**	? 7 days	? 10 days	✗ 6 days	✓ 30 days	✓ 14 days	✗ Up to 18 hours	? 7 days	✓ >1 year	✗ 4 days
**Charging**	? 2 hours	? 2 hours	✓ Up to 1 hour	✗ 2-3 hours	✗ Approx. 3 hours	? 1.5 hours	? 60-90min	✗ 60-90min	✓ Approx.. 1 hour
**Water resistance**	✓ Yes (50m)	✓ Yes (50m)	✓ Yes (50m)	✓ 1m for 30 min	✓ 1m for 30 min	✓ Yes (50m)	✓ Yes (for swimming and showering)	✓ Yes (or swimming and showering)	✓ Yes (30m)
**Materials and ease of cleaning**	✓ Silicone band, aluminium buckle	✓ Silicone band, Plastic, metal and silicone clip	✓ Silicone band	✓ Plastic/silicone strap	? Fabric ankle strap	✓ Fluorocarbon rubber (FKM)	✓ Silicone band	✓ Thermo polyurethane or silicone band	✓ Silicone band
**Usability (Amazon user reviews)**	✓ 4.3/5 stars	✓ 4.4/5 stars	✓ 4.4/5 stars	✗ Unable to find review	✗ Unable to find review	✓ 4.6/5 stars	✓ 4.2/5 stars	✓ 4.2/5 stars	? 3.9/5 stars
**Data access (clinicians and patients)**	✓ Fitbit app (iPad) and Fitabase software (remote view)	✓ Fitbit app (iPad) and Fitabase software (remote view)	✓ Fitbit app (iPad) and Fitabase software (remote view)	✓ CentrePoint software dashboard (iPad and remote viewing)	✓ CentrePoint software dashboard (iPad and remote viewing)	? CareKit software (iPad and remote) Requires iPhone	? Garmin Connect app (may require smartphone). Fitabase (remote view)	? Garmin Connect app (may require smartphone). Fitabase (remote view)	? Polar Flow app (may require smartphone). Unable to find remote view software
**Data access (export for research)**	✓ Fitabase	✓ Fitabase	✓ Fitabase	✓ CentrePoint software compatible.	✓ Actilife and CentrePoint software compatible.	? ResearchKit (requires iPhone to use)	✓ Fitabase	✓ Fitabase	? Unable to find a software platform to access
**Data storage life**	? Minute by minute motion data: 7 days Daily totals: 30 days	? Minute by minute motion data: 7 days Daily totals: 30 days	? Minute by minute motion data: 7 days Daily totals: 30 days	✓ 30 days/512 MB	✓ 180 days/4 GB	? 32GB capacity. Not described in terms of activity data storage	? Timed activities: 7 days Activity tracking: 14 days	? 24/7 activity data: >1 month	? 32MB storage. Not provided details on syncing etc.
**Available metrics**	Steps, distance, calories, floors climbed, active minutes, heart rate, sleep tracking, sleep stages	Steps, distance, calories, floors climbed, active minutes, heart rate, sleep tracking, sleep stages	Steps, distance, calories, floors climbed, active minutes, heart rate, sleep tracking, sleep stages	Raw acceleration, Activity intensity, moderate-vigorous physical activity, steps, calories, MET rates, locomotion, total movement, total sleep time, sleep efficiency, wake after sleep onset, sleep fragmentation	Raw acceleration, Activity intensity, moderate-vigorous physical activity, steps, calories, MET rates, locomotion, total movement, total sleep time, sleep efficiency, wake after sleep onset, sleep fragmentation	Steps, active calories, exercise minutes, stand hours, stand minutes, walk distance, cardio fitness (V02 max), walking pace	Steps, distance, calories, active minutes, Sp02, heart rate, sleep tracking, VO2 max, respiration	Steps, distance, calories, active minutes, Sp02, sleep tracking, respiration	Step count, active time (low, medium, high), calories, distance, heart rate, sleep amount, sleep 'solidity' (quality)

**Cost**	≤$200	$200-400	>$400
**Alternate wear location**	Yes	N/A	No
**Wrist worn accuracy**	Yes	Unable to find, or details unclear	No
**Ankle worn accuracy**	Yes	Unable to find, or details unclear	No, or can't be worn on ankle
**Compatibility with iPad**	Yes	Unclear	No, or only if using smartphone also
**Battery life**	≥2 weeks	1-2 weeks	<1 week
**Charging**	≤1 hour	1-2 hours	≥2 hours
**Water resistance**	Yes	Unclear/unable to find	No
**Materials and ease of cleaning**	All materials that can be sterilised with anti-bacterial wipes	Most materials that can be sterilised with anti-bacterial wipes	No materials that can be sterilised with anti-bacterial wipes
**Usability (Amazon user reviews)**	4+ stars	<4 stars	Unable to find
**Data access (clinicians and patients)**	Simple software to enable patient view, and remote access for clinician	Software available for patient and clinician access, but additional equipment required.	Unable to find, or no solution
**Data access (export for research)**	Software available for access to complete and detailed datasets	Software available for researcher access, but additional equipment required.	Unable to find, or no solution
**Data storage life**	>28 days storage of detailed data	Limited days storage of detailed activity data, or unclear level of detail of data stored without sync	Daily syncing required for data storage

What do you think is the best approach to wear location of the wearable activity tracker for the pilot trial?

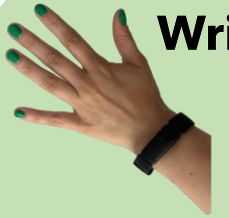

Wrist-worn only

Ankle-worn only

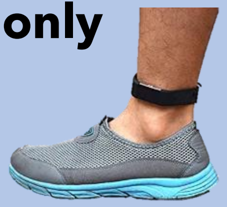

Wrist AND ankle worn (each patient)

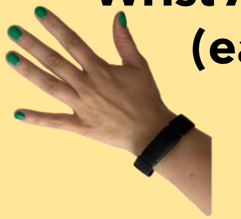

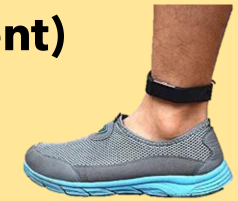

Supplement: Supplementary file 3 [file Datasheet3.pdf]
