# Supplementary material for: Stepping toward implementation using co-design: development of hospital protocols and resources for using wearable activity trackers in a hospital service
Source: Front Digit Health. 2025 Mar 18;7:1520991. doi: 10.3389/fdgth.2025.1520991 (PMC11959083; doi:10.3389/fdgth.2025.1520991)
Supplement: Supplementary file 4 [file Datasheet4.pdf]

	Pre-journey (Pre-admission)	Admission	During rehab	Pre-discharge	Post-journey (Discharge)	Post-discharge
Patient journey	Referred to VRW (receive information and education on what VRW is)	Admitted to VRW & receive iPad, receive education and information regarding iPad use	Use iPad to rehab, receive continuous support and information regarding iPad use	Complete final assessment	Complete therapy OR readmitted to hospital, iPad returned to service (post OR drop off OR clinician collects)	iPad not returned/damaged
Clinician journey	Receive patient referral, assess patient eligibility, provide information and education on what VRW is	Receive programmed iPad & give to patient, provide education and information regarding iPad use	Provide virtual care/home visits, provide continuous support and solve problems, contact tech team for additional support as needed	Complete final assessment	Receive iPad & clean, give to tech team	
Tech journey	Set up iPad for patient	Give iPad to VRW	Higher-level troubleshooting	Notified re PT ready to D/C	Receive and reset iPad	Follow up with patient, initiate insurance protocol

		Pre-journey (Pre-admission)	Admission	During rehab	Pre-discharge	Post-journey (Discharge)	Post-discharge
Technology	User interface		X	X			
	Connection and syncing issues			X			
	Limited scope and function of iPad		X	X			
Clinician	Clinician inexperience with iPads	X	X	X	X		
	Poor coordination between services		X	X	X		
	Practitioner reluctance to change		X	X			
Patient	Patient tech literacy and trust	X	X	X			
	Patient engagement		X	X			
	Patient suitability for rehabilitation		X	X	X		

	Pain points	Examples	Possible solutions
Technology problems	**User interface**	Difficulty navigating apps, device is too complex for patients with poor cognition (icons, language, navigating between apps).	Provide information on how to navigate each.<br>Use of colour and words in a guided process.<br>Minimise patient demands and responsibilities.
	**Connection and syncing issues**	Videoconferencing not working at patient and/or clinician end. Issues with internet connection. Monitoring devices (e.g. blood pressure cuff, pulse oximeter) not connecting to iPad, iPad not syncing to electronic health records.	Type of device and software use are a fit for the service.<br>Include instructions for connection and syncing.
	**Limited scope and function of iPad**	Can't conduct comprehensive assessment or therapy via iPad/video conferencing (e.g. camera angles, safety concerns, poor audio/video, remote clinical practice).	Type of device and software use are a fit for the service.
Clinician problems	**Clinician inexperience with iPads**	Practitioners may default to home visits if having challenges with iPad, poor tech literacy (possibly due to limited training time), concerned about safety and effectiveness of remote care.	Training, education and information provided.
	**Poor coordination within team**	Miscommunication regarding care provided (e.g. exercises, education, home visits), miscommunication through excel booking system .	Make roles clear.<br>Daily communication about WAT us amongst team (e.g. checking if charged).
	**Practitioner reluctance to change**	Limited ability to provide therapy as per usual standard (e.g. objective tests, equipment, performing certain exercises). Concerned about patient safety (e.g. falls risk). Don't know how to use iPad, don't want to change routines. May default to in-person Ax and Rx.	Education regarding importance, provided to improve confidence.<br>Top-down approach to influence and encourage use.
Patient problems	**Patient tech literacy + trust**	Patient unsure of data security/privacy, inexperience with iPad and low tech-literacy. May reduce use of iPad and engagement with therapy.	Education and information provided.<br>Procedures designed to place minimal demand on patients and simplify responsibilities.<br>Clinicians are enthusiastic.<br>Patient-centred language is used.
	**Patient engagement**	Not engaged with therapy in general: lack of trust, poor understanding of why, low self-efficacy, motivation, and stage of change, influence from family and other support systems, patient is bored or not interested.	Clinicians are enthusiastic and encouraging.<br>Limit demands and responsibilities for patients.<br>Link WAT use to personal goals and situation.<br>Rewards provided for reaching goals/milestones.<br>Use behaviour change strategies (i.e. goal setting).
	**Patient suitability**	Not cognitively able to manage iPad. Inappropriate referral: not safe or medically stable for rehabilitation. Not ready to go home/be discharged and readmitted to higher levels of care.	Clinicians assess suitability of patients, and if adaptations are needed.

Journey map feedback

- Activity monitoring relied on old technology - diaries, patient report, patient memory
- Assumption that patient can't use technology (they could be selected out)
- Patient actually wants visitors in person as it breaks their day/weeks up
- Patient dexterity – physically unable to press apps
- Involvement of family members/carers! Quite often there are many barriers to using technology (especially for elderly people) however when they have carers or family to assist, things run much smoother usually
- Patient not willing to try technology from the get go (*Patient engagement category*)
- Patients refusing to use technology
- Visually impaired patient, hearing impaired patients (*Patient suitability category*)
