# Supplementary material for: Stepping toward implementation using co-design: development of hospital protocols and resources for using wearable activity trackers in a hospital service
Source: Front Digit Health. 2025 Mar 18;7:1520991. doi: 10.3389/fdgth.2025.1520991 (PMC11959083; doi:10.3389/fdgth.2025.1520991)
Supplement: Supplementary file 5 [file Datasheet5.pdf]

USING YOUR FITBIT DURING REHABILITATION

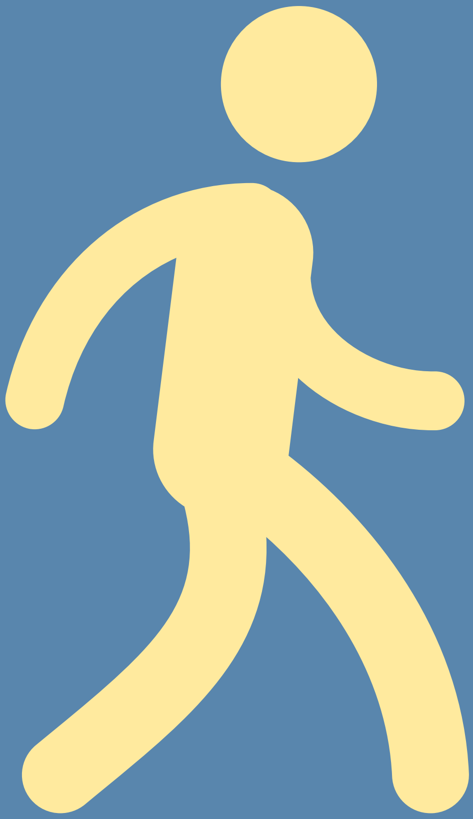

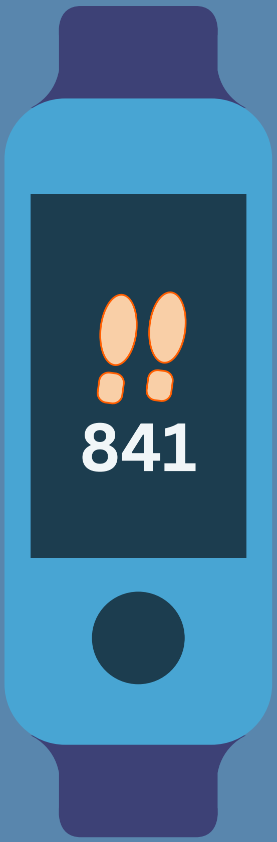

University of South Australia
Alliance for Research in Exercise, Nutrition and Activity
Government of South Australia
Health
Southern Adelaide Local Health Network

CONTENTS

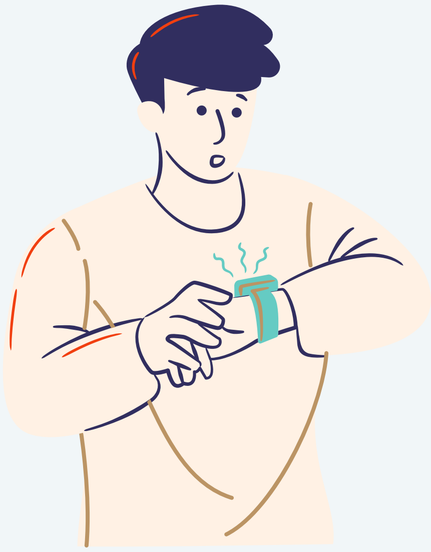

Overview 1

Using your Fitbit 2

Wearing your Fitbit 3

Opening the Fitbit app 4

Syncing 5

Tracking your step count 6

Charging 9

Daily log 11

Daily checklist 15

OVERVIEW

1 What happens at the start of your admission?

Set therapy goals and step goals with therapist

2 What happens during your admission?

Outside of therapy time

- Keep track of your steps throughout the day
- Try and meet your daily step goal!
- Write down how many steps you have done at the end of the day

During therapy time

- Discuss your progress with therapists
- Review your daily step goals
- Ask your therapist any questions that you have

3 What happens at the end of your admission?

- Review your therapy goals and step goals with therapist.
- Return Fitbit and charger to hospital with iPad.

University of South Australia
Alliance for Research in Exercise, Nutrition and Activity
Government of South Australia
Health
Southern Adelaide Local Health Network

USING YOUR FITBIT

WHAT WILL YOU HAVE TO DO?

1. **Wear your Fitbit**
   This will make sure that it records your activity

2. **Keep track of step counts throughout the day**
   So you can see how far you are from your daily goal

3. **'Sync' Fitbit with your iPad**
   This makes sure your activity is saved each day, and will let you track your step counts on the iPad. This should happen automatically, but can also be done manually.

4. **Charge your Fitbit**
   This makes sure it measures all of the activity you do while wearing it, and you can keep track of your steps

OPTIONAL

Record your steps each day using the log sheet

You can keep track of your daily steps on the log sheet provided, and see if you're meeting your goals regularly. You can also use this to discuss your progress with your therapist.

University of South Australia
Alliance for Research in Exercise, Nutrition and Activity
Government of South Australia
Health Southern Adelaide Local Health Network

WEARING YOUR FITBIT

Where to wear it

Wear your Fitbit on your wrist, about a finger width above the wrist bone.

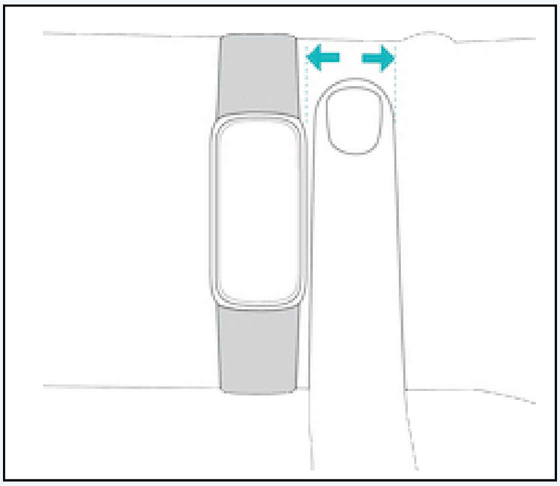

How to put it on

Place your device around your wrist, and fasten the clasp

When to wear it

Wear it continuously and as often as you can! Even while you sleep or bathe. The more you wear it, the more step counts it will measure. A minimum of 12 hours of wearing every day is needed, but more is better!

Can I get it wet?

The Fitbit is water resistant, so it's safe to wear while bathing or during water-based activity like hydrotherapy.

Can I take it off?

If you find the Fitbit uncomfortable while you sleep or bathe, you can take it off, but remember to put it back on afterward. Keeping it somewhere visible will help you remember to put it back on.

OPENING THE FITBIT APP ON THE IPAD

Tap on the aqua 'Fitbit' icon it looks like this:

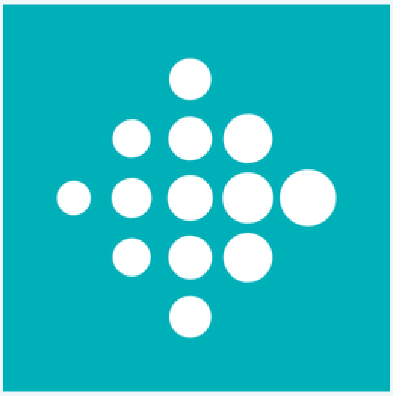

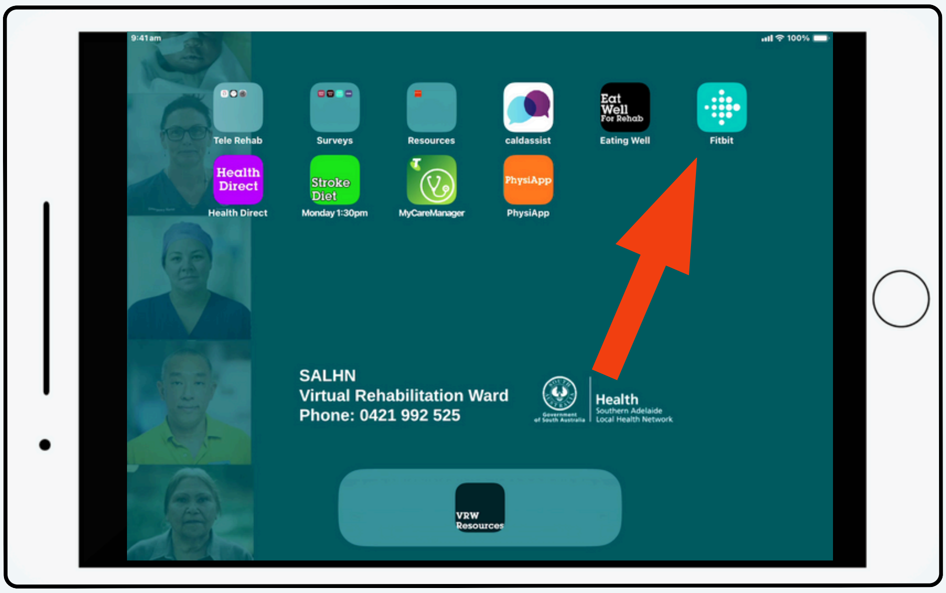

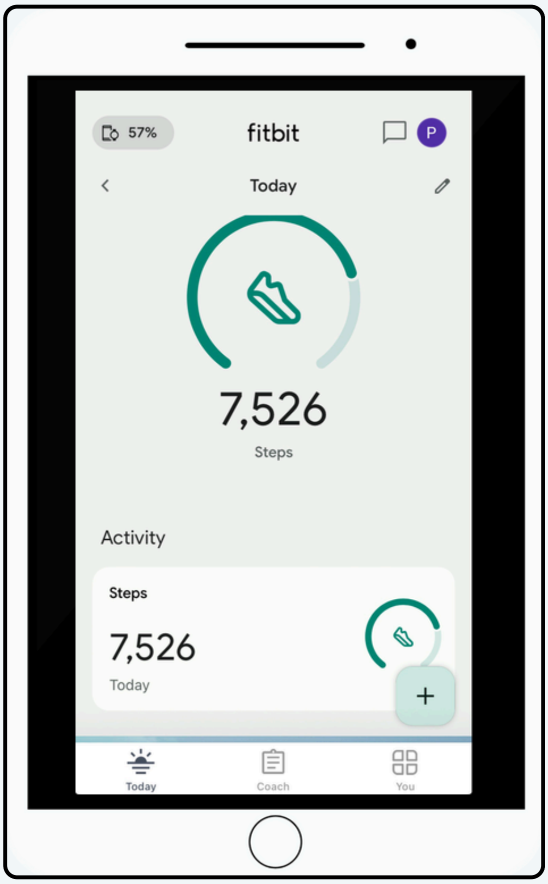

This will open the Fitbit app, which will look a bit like the picture to the left.

Turning the iPad to portrait mode will make it easier to read.

Syncing the Fitbit with the iPad

The Fitbit should sync with the iPad automatically. If it is not, this can be done easily by following the instructions below.

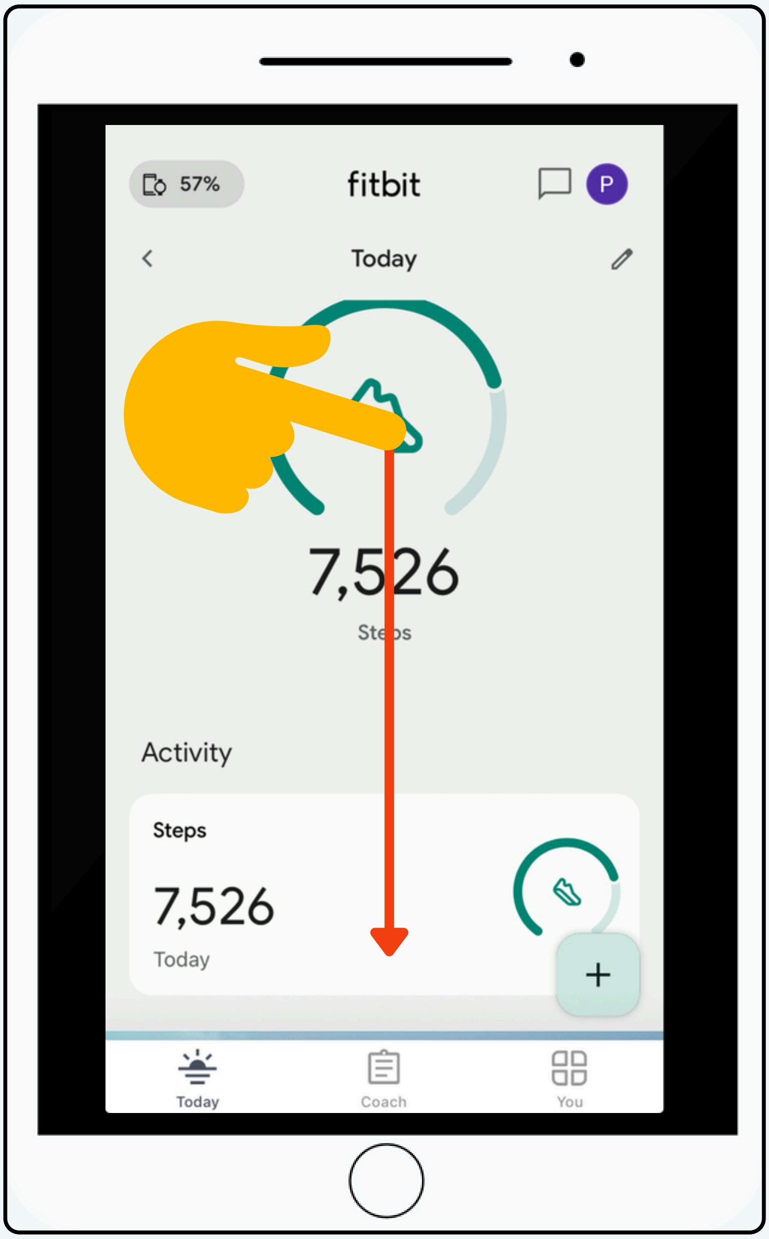

1. Open the Fitbit app on the iPad
2. Tap the icon to go to the 'Today' tab.
3. Swipe down on the screen.
4. Wait 30-60 seconds for the Fitbit and iPad to sync.

TRACKING YOUR STEP COUNT

On the Fitbit

1

Wake up the screen

Either:

- Tap the screen twice
- Tap both sensors on the sides at the same time

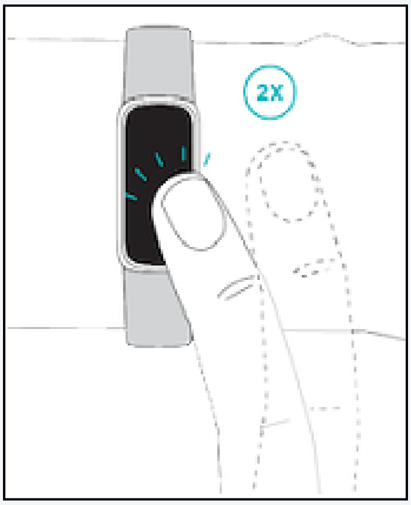

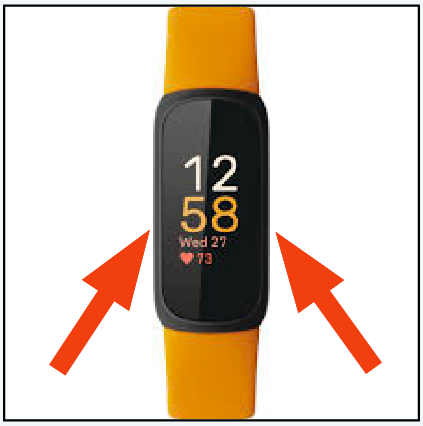

2

Swipe up to see your daily steps

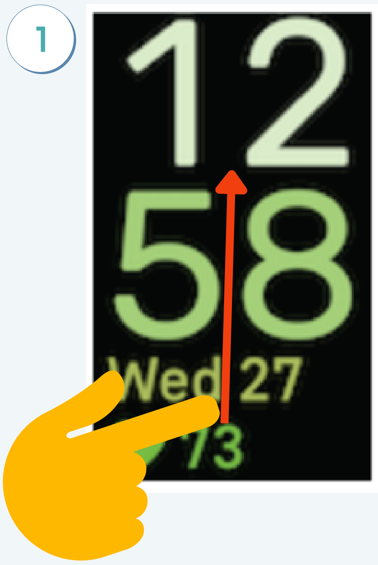

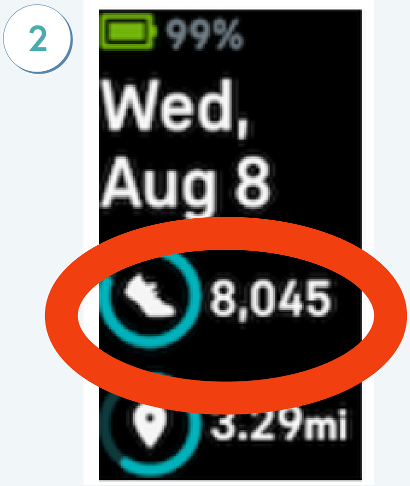

TRACKING YOUR STEP COUNT

On the iPad

Today's step count

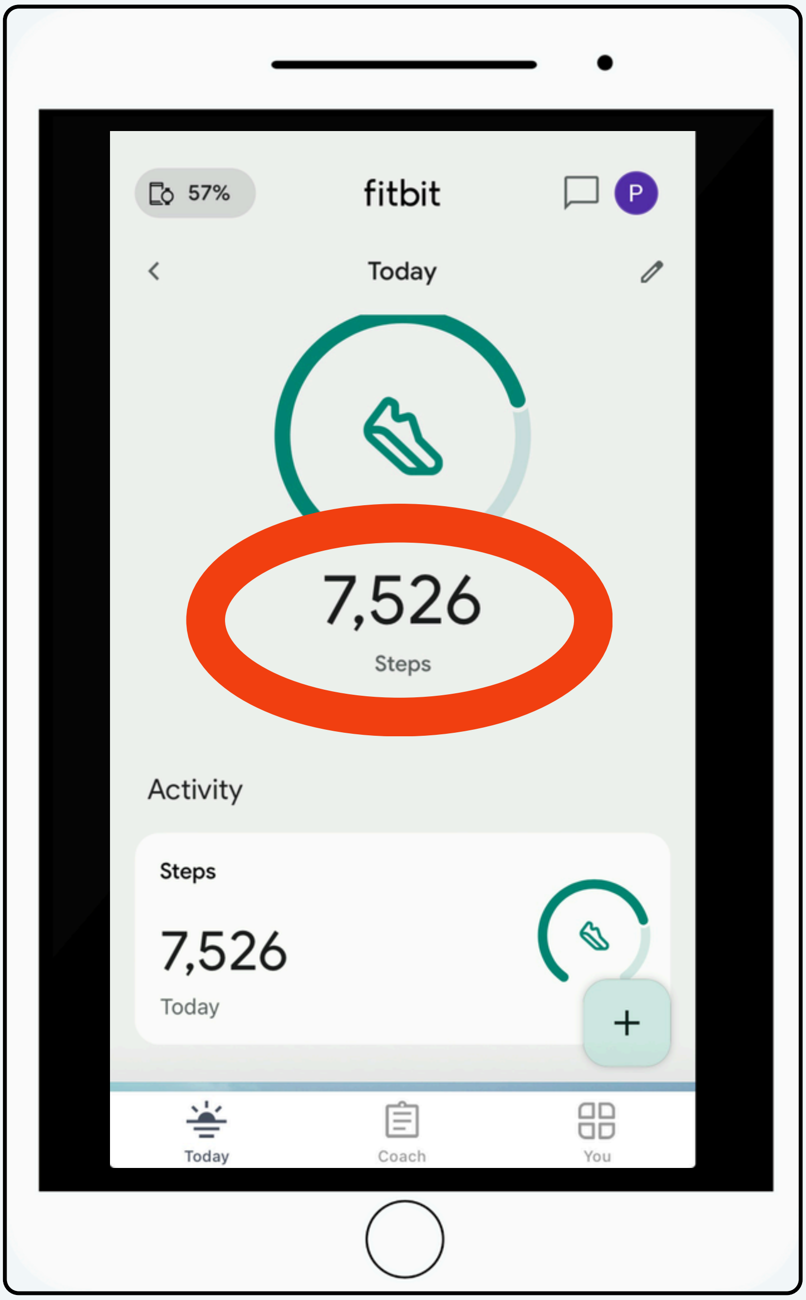

1. Open the Fitbit app on the iPad.
2. Tap the icon to go to the 'Today' tab.
3. Check that the Fitbit has synced (see page 4).
4. The step count will be displayed on the screen under the icon.

University of South Australia | Alliance for Research in Exercise, Nutrition and Activity | Government of South Australia | Health Southern Adelaide Local Health Network

TRACKING YOUR STEP COUNT

On the iPad

Previous days' step counts

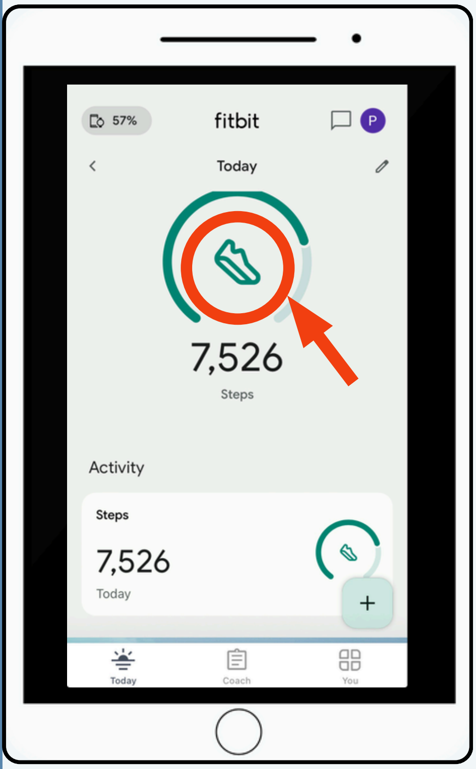

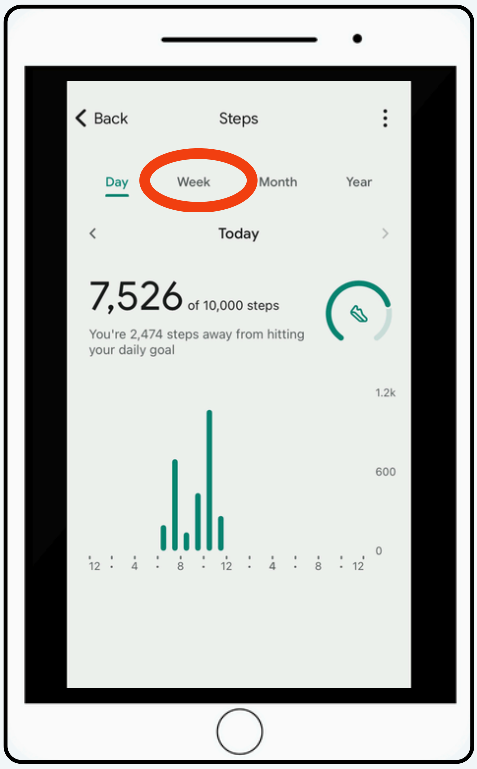

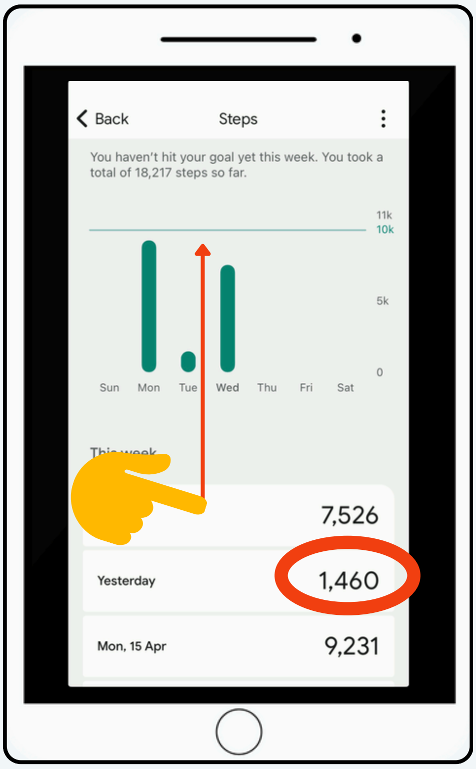

1. From the 'Today' tab, tap on the 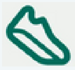 icon

2. Tap the 'Week' tab at the top of the screen to see previous days' step counts.

3. Scroll down to view all previous days recorded step counts.

CHARGING

When to charge?

- Charge your Fitbit **every Sunday for 1-2 hours** until it is fully charged.
- OR if your Fitbit has **less than 30%** of battery life.

Checking if the Fitbit is charged (on the Fitbit)

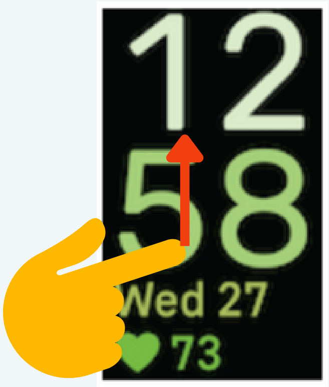

1. Swipe up from the clock face.

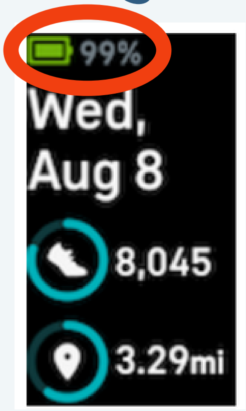

2. The battery level is in the top left.

Checking if the Fitbit is charged (on the iPad)

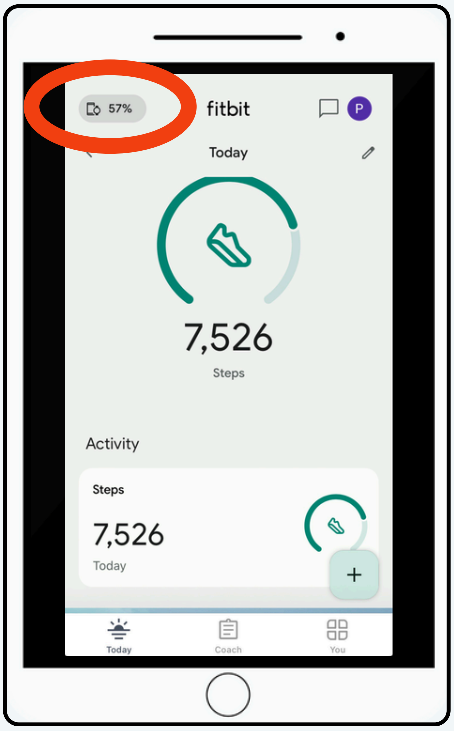

1. Open the Fitbit app on the iPad
2. Tap the icon to go to the 'Today' tab.
3. The battery level is displayed in the top left corner, next to the icon.

CHARGING

How to charge

1. Check that the charging cable is plugged in and the power is on.
2. Line up the pins on the end of the charging cable with the gold points on the back of your Fitbit.
3. Gently press your Fitbit into the charger until it clicks into place.
4. Let your Fitbit charge for 1-2 hours.

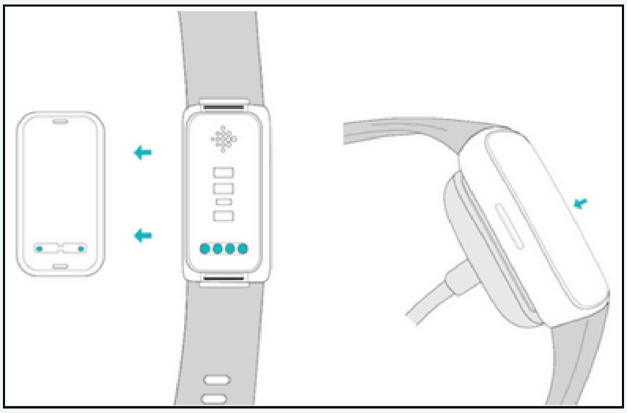

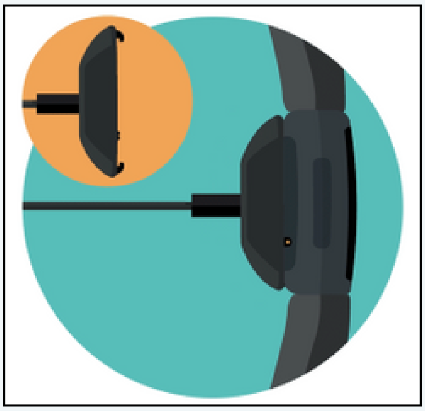

How to remove the charger

1. Hold the Fitbit and slide your thumbnail between the charger and the back of the Fitbit on one of the corners.
2. Gently press down on the charger until it separates from your device.

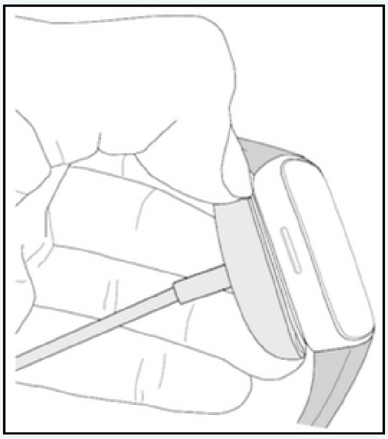

MY STEP TRACKING LOG

Day	Step goal	Steps taken
Mon		
Tues		
Wed		
Thurs		
Fri		
Sat		
Sun		
Mon		
Tues		
Wed		
Thurs		
Fri		
Sat		
Sun		

MY STEP TRACKING LOG

Day	Step goal	Steps taken
Mon		
Tues		
Wed		
Thurs		
Fri		
Sat		
Sun		
Mon		
Tues		
Wed		
Thurs		
Fri		
Sat		
Sun		

MY STEP TRACKING LOG

Day	Step goal	Steps taken
Mon		
Tues		
Wed		
Thurs		
Fri		
Sat		
Sun		
Mon		
Tues		
Wed		
Thurs		
Fri		
Sat		
Sun		

MY STEP TRACKING LOG

Day	Step goal	Steps taken
Mon		
Tues		
Wed		
Thurs		
Fri		
Sat		
Sun		
Mon		
Tues		
Wed		
Thurs		
Fri		
Sat		
Sun		

DAILY CHECKLIST

Check these throughout the day

- [ ] I'm wearing my Fitbit as much as possible
- [ ] I'm on track to meeting my step goal for today!

Check these at the end of the day

- [ ] My Fitbit is charged above 30%
- [ ] My Fitbit has synced with the iPad

Optional

- [ ] I have recorded my daily step count

This can be done the following day. See page 6 for instructions on checking previous days' step count.
