# Supplementary material for: Stepping toward implementation using co-design: development of hospital protocols and resources for using wearable activity trackers in a hospital service
Source: Front Digit Health. 2025 Mar 18;7:1520991. doi: 10.3389/fdgth.2025.1520991 (PMC11959083; doi:10.3389/fdgth.2025.1520991)
Supplement: Supplementary file 6 [file Datasheet6.pdf]

Using Fitbits with patients during rehabilitation

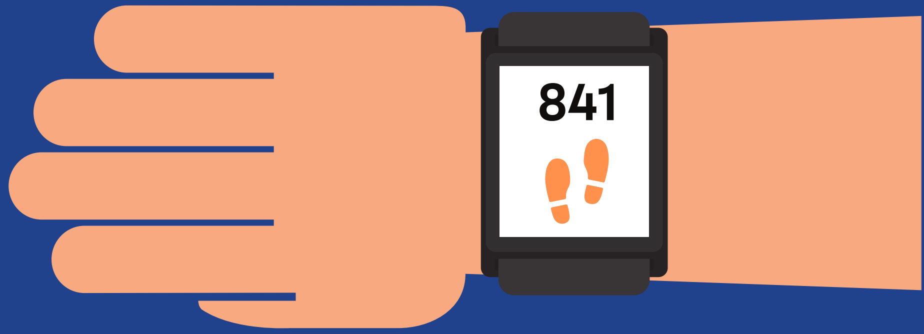

Project overview

An overview of what will happen before admission, during admission, and after discharge is outlined in the flow chart.

Clinicians' main responsibilities will occur during the patients' admission. This will involve reviewing patients' daily step counts, setting and reviewing step goals, and assisting the patient with basic operation of the Fitbit. Clinicians will also facilitate the researcher in reviewing patient referrals for eligibility to participate.

Click the link below for a goal setting guide for the service you are working in:

- Virtual Rehabilitation Ward
- FIT Program

The researcher will be responsible for setting up Fitbit devices and accounts for patients, will facilitate patient introductory sessions to using Fitbits, and provide support throughout the patient's admission with using Fitbits and the associated Fitabase software

Pre-admission

- Researcher and clinician review patient referrals for eligibility
- Eligible patients invited to participate
- Patient provided Fitbit and Fitbit account set up on device at earliest time available after providing consent.

During admission

1. What happens at the start of admission?

- Set therapy goals and step goals with patient.
- Go through basic Fitbit operations with patient.

2. What happens during admission?

Outside of therapy

- Clinician monitors patient activity (step counts) and use of Fitbit using Fitabase software.
- Patient uses Fitbit to self-monitor steps, and tries to meet daily step goals.

During therapy

- Clinician discusses progress toward step goals with patient.
- Daily step goals adjusted if needed (i.e. too easy or too hard)
- Patient ask clinicians any questions that they have.

3. What happens at the end of admission?

- Final review of patient's therapy goals and step goals.
- Return Fitbit to hospital.

Post-discharge

- Researcher to check final sync of Fitbit, disconnect and clean Fitbit ready for next participant to use.

Using Fitbits with patients

You will need to help the patient with basic operation of the Fitbit. The tasks that the patient will need to perform are displayed in the next section. The patient will manage most of these task at home, but you will need to step them through how to do these when they are admitted, and help them if they encounter any difficulties during admission.

Instructions for how to perform each of these tasks can be found in the patient handbook. Click the link below to access the patient handbook

Patient handbook

Wearing the Fitbit
This will ensure that it records patient activity

Keep track of step counts throughout the day
To monitor progress toward goals and motivation for increasing activity levels.

'Sync' Fitbit with the iPad
This ensures that tracked activity is saved each day, and will let enable step tracking on the iPad

Charge the Fitbit
This ensures it captures all of the activity the patient does while wearing it, and enables the patient to keep track of their steps

Optional: Record steps each day
This enables the patient to track if they're meeting their goals over time, can provide motivation, and can be used during therapy to discuss progress with the patient and review goals.

Using Fitabase software

Click here to log in to Fitabase

You will be able to review the patients' daily step counts and management of their Fitbit using Fitabase. You will be able to view this information remotely from the hospital, and you'll be able to navigate between different patients without having to log in/out of separate accounts. Fitabase can be accessed using a web browser, and is intuitive to navigate. You will receive login details for an account linked to the service that you work in. The functions you will need and links to instructions are in the listed in the next section.

Video instructions for how to use Fitabase can be found on YouTube, and print instructions can be found in a clinician handbook.

Clinician Fitabase handbook

Youtube Fitabase playlist

Logging in and accessing the project
Fitabase is accessed using the web browser, and you can log in from any computer.
For instructions, click here.

Finding a patient
All participating patients can be found from the project profile.
For instructions on finding a patient, click here.
For instructions on navigating between patients, click here.

Reviewing a patient's activity metrics
You can review patients' step counts and other activity data on Fitabase.
For instructions on viewing activity, click here.
For instructions on adjusting the time frame displayed, click here.

Checking device management
You can monitor if patients are syncing, charging, and wearing their Fitbit on Fitabase.
For instructions on monitoring syncing and charging, click here.
For instructions on monitoring wearing, click here.

Need more help?

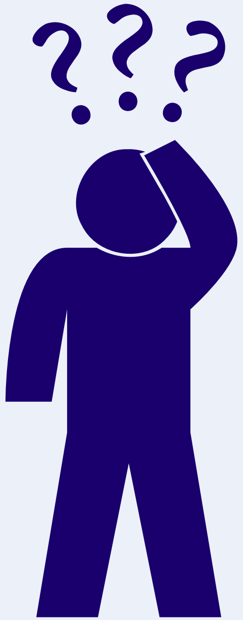

If you're having difficulty using Fitbits or Fitabase, contact Kimberley Szeto (research project manager)

Email: kimberley.szeto@mymail.unisa.edu.au
